# CeDAR: incorporating cell type hierarchy improves cell type-specific differential analyses in bulk omics data

**DOI:** 10.1186/s13059-023-02857-5

**Published:** 2023-02-28

**Authors:** Luxiao Chen, Ziyi Li, Hao Wu

**Affiliations:** 1 Department of Biostatistics and Bioinformatics, Emory University, GA 30322 Atlanta, USA; 2 Department of Biostatistics, The University of MD Anderson Cancer Center, 77030 Houston, TX, USA; 3 Faculty of Computer Science and Control Engineering, Shenzhen Institute of Advanced Technology, Chinese Academy of Sciences, 1068 Xueyuan Avenue, Shenzhen University Town, Shenzhen, 518055 P.R. China

**Keywords:** Cell type-specific differential analysis, Cell type hierarchy, Hierarchical Bayesian model, Microarray data analysis

## Abstract

Bulk high-throughput omics data contain signals from a mixture of cell types. Recent developments of deconvolution methods facilitate cell type-specific inferences from bulk data. Our real data exploration suggests that differential expression or methylation status is often correlated among cell types. Based on this observation, we develop a novel statistical method named CeDAR to incorporate the cell type hierarchy in cell type-specific differential analyses of bulk data. Extensive simulation and real data analyses demonstrate that this approach significantly improves the accuracy and power in detecting cell type-specific differential signals compared with existing methods, especially in low-abundance cell types.

**Supplementary Information:**

The online version contains supplementary material available at 10.1186/s13059-023-02857-5.

## Background

The bulk high-throughput omics experiments are often performed on tissue samples, which are mixtures of different cell types. Traditional bulk data analyses for differential expression (DE) and differential methylation (DM) compare the average signals among different groups. However, it has been reported that certain biological and clinical conditions can alter the DNA methylation or gene expression profile in specific cell types. For example, Grubman et al. reported that Alzheimer’s disease (AD) risk gene APOE shows cell type-specific different expression patterns: it is upregulated for AD in microglial cells, but downregulated in both oligodendrocyte progenitor cells and astrocytes [1]. Gu et al. reported that neuron and glia cells show different DNA methylation pattern within SNCA intron 1 in two synucleinopathies—Parkinson’s disease (PD) and dementia with Lewy body (DLB) [2]. In PD, decreased DNA methylation within SNCA intron 1 only appears in neuron cells, while in DLB, it only appears in glia cells. These cell type-specific changes are important for understanding biological and clinical mechanisms and potentially provide diagnostic biomarkers and therapeutic targets. Thus, researchers often have great interest in identifying cell type-specific alterations under various conditions.

Experiment procedures such as cell sorting or single-cell approaches can directly measure the cell type-specific behaviors. However, the two technologies are laborious and expensive, which limits their large-scale application. While the traditional DE/DM methods for bulk data only compare the average signals, recent development of computational methods makes it possible to perform cell type specific analysis from the bulk data. The cell type-specific analysis on bulk omics data has been an active research field recently. There are several methods developed for signal deconvolution and cell type-specific inference. For example, csSAM [3] adopts a two-step approach: it first estimates pure cell type profiles based on known cell type proportions and then conducts permutation tests to identify cell type-specific DE (csDE). Both CellDMC [4] and TOAST [5] use interaction terms between covariates and cell type proportions in a linear model to test csDE/csDM. This statistical framework has been shown as a generalization of several previous works [6–8]. TCA [9] models the cell type-specific methylation levels of each individual and derives a procedure for cell type-specific inference. While CellDMC, TOAST, and TCA mainly focus on continuous methylation or gene expression data measured in microarray, CARseq [10] is designed for cell type-specific inference for count data from RNA-sequencing by using a negative binomial (NB) distribution. Different from previous mentioned methods that require known cell type composition as input, HIRE [11] jointly perform composition estimation and csDM inference. Even though these methods generally achieve satisfactory performance in detecting differential signals from abundant cell types, their accuracy and power could be low, especially in cell types with small proportions. Using the existing methods, the only way to improve the results for those minor cell types is to increase sample size, which could be infeasible in many settings.

It is known that different cell types in a tissue form a hierarchical structure [12, 13]. For example, the major groups of lymphocytes include natural killer cells (NK), T cells, and B cells. The T cells can be further divided into many subtypes including CD4+ T cells (CD4) and CD8+ T cells (CD8). Due to the similarity among cell types, it is conceivable that similar cell types could exhibit similar DE or DM patterns, e.g., if a gene is DE in CD4, it is more likely to be also DE in CD8. Correlations of DE/DM states among cell types have been reported in many published works. Mathys et al. [14] reported that in the late stage of AD, genes upregulated were common across cell types and primarily involved in global stress response. Tserel et al. [15] reported that age-related methylation changes (measured by fold change) in CD4+ T cell and CD8+ T cell have a strong correlation and that all top sites with the highest methylation differences between younger and older individuals are shared by the two cell types. In a Graves’ disease (GD) study, Limbach et al. [16] reported that a majority of the most significant CpG sites associated with GD had differential methylation in both CD4+ and CD8+ T cells. Conceptually, the similarity of DE/DM status among cell types can be exploited to improve the csDE/csDM results. In this work, we develop a novel and rigorous statistical method to incorporate the cell type hierarchy into the cell type-specific differential analysis in high-throughput bulk omics data. Our proposed method borrows information across cell types through a Bayesian hierarchical model. A key intuition of the proposed method is that the prior probability of one gene being DE in a cell type is impacted by the DE status of this gene in other cell types, for example, if gene A shows strong DE in CD4, its prior probability of being DE in CD8 will be higher due to the similarity between CD4 and CD8. We name the proposed method “Cell type-specific Differential Analysis with tRee” (CeDAR) and implement it in Bioconductor package TOAST (https://www.bioconductor.org/packages/release/bioc/html/TOAST.html). In the sections below, we first motivate the proposed method by illustrating the DE/DM correlation among cell types in real data exploration. We then provide an overview of the proposed method. Following that, we comprehensively evaluate the proposed method with both simulated and real data. The results demonstrate that incorporating the cell type hierarchy in the csDE/csDM framework greatly improves the detection performance, especially in cell types with low proportions.

## Results

### Strong correlations of DE/DM states among cell types are observed in real data

We performed real data analyses to explore whether the DE/DM states are correlated among cell types in real data. We obtained two datasets from Gene Expression Omnibus (GEO) database, one DNA methylation [17] and one gene expression [18]. Both datasets contain samples of purified cells from individuals under different conditions; thus, the gold standard results are available. We first called DM and DE for each cell type in these two datasets using existing tools. We called DM between males and females in the DNA methylation data and called DE for sclerosis patients before versus after first IFN-beta treatment. Detailed description for the data and analysis procedures is in the “Methods” section. Then, we evaluated the pairwise correlation among cell types in terms of their DE/DM status, using both Pearson correlation coefficient (PCC) of log-transformed *p*-values from the DE/DM tests for all features, and the odds ratio (OR) of being DE/DM from the cell types. The first metric (PCC) evaluates the correlations at the quantitative level that consider the DE/DM strength, while the second metric (OR) evaluates the correlation at the qualitative level since it quantifies the concordance of the binary DE/DM status. Higher PCC and OR indicate stronger correlation among cell types.

**Fig. 1:**
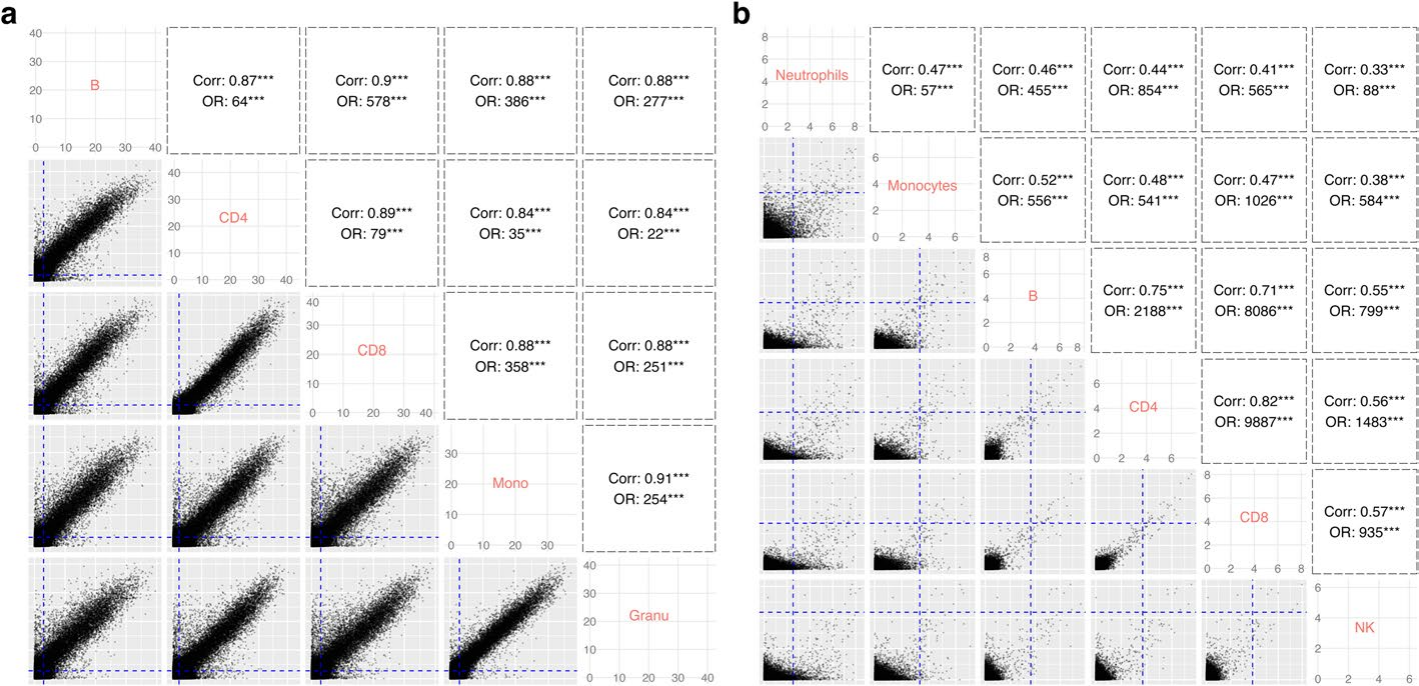
Correlations among cell types from cell type-specific differential analysis. **a** Cell type-specific differential methylation analysis and **b** cell type-specific differential expression analysis. DE/DM tests were applied for each feature in each cell type. *X*-axis and *Y*-axis represent -log10 transformed *p*-value from DE/DM tests in corresponding cell types. Each point represents a gene or CpG site. Dashed blue lines represent the thresholds used to define DEG/DMC in each cell type. Pearson correlation coefficients (PCC) of transformed *p*-values and odds ratio (OR) of differential state are tested for their significance. *** represents *p*-value < 0.01

The pairwise scatterplots for the comparisons are shown in Fig. 1. In the DNA methylation data (Fig. 1a), the *p*-values from all cell types are highly correlated (all PCCs > 0.83). Besides, the ORs for being DM between any two cell types are all very large. These results indicate very strong correlation among cell types in their methylation difference between males and females. In gene expression data (Fig. 1b), all PCCs are also significantly positive and all ORs are significantly greater than 1. The correlation strength appears to be weaker in the gene expression example than in the methylation data since the molecular differences between sexes (as considered in the methylation data) are likely to be much stronger than the treatment effects (as considered in the gene expression data). Additionally, the gene expression dataset shows different levels of correlation among cell types. For example, B cells, CD4, and CD8 are more correlated with each other compared to others (PCCs > 0.7), suggesting a cell type hierarchy. Similar results are observed by performing the same analyses on three additional real datasets (Additional file 1 Section S5, Figure S1). Overall, these results demonstrate that there are strong correlations among cell types in terms of their DE/DM status.

### Method overview

CeDAR incorporates the cell type hierarchy in cell type-specific differential analysis in bulk data. Briefly speaking, for each feature, we define binary random variables to represent its underlying DE/DM states in all cell types, each with a prior probability. Given a realization of the DE/DM states for all cell types, we model the observed bulk data using a linear model framework similar to TOAST and CellDMC, in which the interaction terms between the cell type proportions and the covariate of interest capture the cell type-specific effects. The unique feature in CeDAR distinguishing it from the existing methods is that the interaction terms are only included for cell types deemed DE/DM. In contrast, TOAST/CellDMC is the full model which implicitly assumes the feature is DE/DM in all cell types, since the interactions are included for all cell types. The marginal likelihood of the observed data can be calculated by summing over all the underlying DE/DM states. Then the posterior probability of a feature being DE/DM in each cell type given observed data can be calculated and used to detect csDE/csDM.

**Fig. 2:**
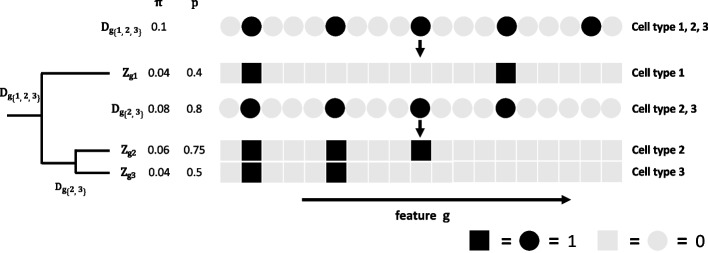
Illustration of the specification of the prior probabilities for DE/DM under a cell type hierarchy. The cell type hierarchy is represented by three cell types and a few features (genes or CpG sites). The three cell types form a simple tree (shown in the left). In the array of squares and circles, each column represents a feature. Circles represent root or internal nodes, and the squares represent leaf nodes. Colors represent the differential states of the node (black: 1; gray: 0). The root node *D*_*g*{1, 2, 3}_, internal node *D*_*g*{2, 3}_, and leaf nodes *Z*_*g*1_, *Z*_*g*2_ and *Z*_*g*3_ are binary random variables representing the *g-*th feature differential states. *π* represents the marginal probability for a node to be in state 1. *p* represents the conditional probability of a node to be in state 1 when its parent node is in state 1

The most important part of the proposed method is the specification of the prior probabilities for the DE/DM status for each cell type. If one only considers the marginal probabilities of DE/DM and assumes independence among cell types, the similarities among cell types cannot be incorporated. In order to take advantage of the correlations among cell types, we make the prior probabilities dependent on the cell type hierarchy. Given a hierarchical tree of cell types, we assign priors for the root and all internal nodes, then compute the priors for the leaf nodes based on the cell type hierarchy. The specification of the prior is graphically illustrated by a toy example in Fig. 2. Assuming there are three cell types forming a simple tree with one root node, one internal node, and three leaf nodes. All nodes have underlying binary states of being DE/DM (state 1) or not (state 0). Here we define a non-leaf node as DE/DM if any of its direct children’s node is DE/DM. Conversely, a child node can be DE/DM only when its direct parent node is DE/DM. The prior probabilities on the non-leaf nodes will implicitly account for the correlations among cell types. For example, even though the marginal probabilities of DE/DM for cell types 2 and 3 are small (0.06, 0.04), their conditional probabilities when the parent node is in state 1 become very high (0.75, 0.5). If a gene shows strong DE in cell type 2, it will increase the probability for its parent node (an internal node) to be DE, which subsequently increases the prior probability for this gene to be also DE in cell type 3. Thus, the correlation between cell types 2 and 3 is passed through their parent node. On the other hand, the distance between cell types 1 and 3 is larger, so their influences on each other must pass through the root and internal nodes, which is weaker. The details of the data model and estimation procedure are in the “Methods” section. It is important to mention that the proposed method allows the cell type hierarchy to be any rooted tree, i.e., it does not have to be a bifurcating hierarchical tree. In the sections below, we show results from different types of tree structures.

### Simulation results

#### CeDAR method improves accuracy in cell type-specific differential signal detection

We conducted simulation studies to compare the performance of CeDAR with TOAST, TCA, csSAM, and CellDMC in a two-group comparison. Although TCA was originally designed for bulk methylation data, the method is also applicable to gene expression data [19]. We incorporated two types of tree structures in the CeDAR test: the first is the simplest tree structure with only one layer (referred to as “CeDAR-S”), where root node is the parent of all leaf nodes. The second is a bifurcating hierarchical tree with multiple layers (referred to as “CeDAR-M”). While CeDAR-M captures a more complex correlation structure among cell types, CeDAR-S avoids the potential negative impacts of the biases in the specified prior tree structure.

**Fig. 3:**
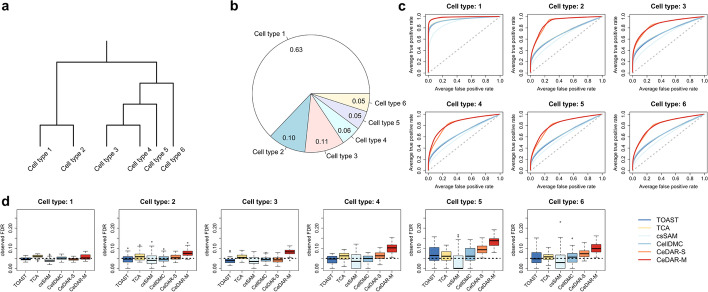
Simulation results for comparing different methods in cell type-specific differential expression. The simulation is based on a two-group comparison, with 100 samples in each group. Data were generated as a mixture of six common blood immune cell types (1: neutrophils, 2: monocytes, 3: CD4, 4: CD8, 5: B, 6: NK cells). **a** Cell type hierarchy used in simulation. **b** Mean proportion of each cell type. **c** ROC curves for csDE detection in six cell types for six methods (TOAST, TCA, csSAM, CellDMC, CeDAR-S, and CeDAR-M). Reported ROC curves are averaged from 50 simulations. **d** Observed FDR for csDE detection from different methods. DE genes are defined with rules: estimated FDR < 0.05 (TOAST, TCA, csSAM, and CellDMC); posterior probability of DE > 0.95 (CeDAR-S, CeDAR-M). Observed FDR from 50 simulations are summarized by box plot

The simulation is constructed based on a dataset (GEO accession number GSE22886 [20]) from whole blood samples with six cell types: neutrophils, monocytes, CD4, CD8, NK, and B cells. We simulated gene expression for six cell types based on parameters estimated from the real data to ensure the simulated data has characteristics (pure profiles and cell type composition) matching the real data. Note that we conducted simulation based on gene expression microarray data, but the proposed method can also be applied to DNA methylation microarray data. We made the six cell types have different levels DE state correlation following a hierarchical tree (Fig. 3a). To be specific, we simulated the strongest correlation between cell types 1 and 2 as well as between cell types 3 and 4, both having ~80% DE genes overlapped. Cell types 5 and 6 are made to have slightly weaker correlations with cell type 3 with ~62*.*5% and ~50% overlapped DE genes, respectively. We simulated the weakest correlation between cell types 1/2 and cell types 3/4/5/6. Between any two of them, only about 12*.*5% DE genes in one cell type overlap with the other. We used the true proportion to conduct data analyses for the results presented in this subsection and will evaluate the impact of proportion estimation in later sections. The accuracy of detecting csDE genes was measured by ROC curve, the area under the ROC curve (AUC-ROC), area under the precision-recall curve (AUC-PR), and Matthews correlation coefficient (MCC). We also evaluated the type I error controls from different methods by examining their false discovery rates (FDR). All methods were evaluated at different sample sizes (50, 100, 200 per group). The results were summarized from fifty simulations. Detailed simulation procedure is in the “Methods” section.

The simulation result shows that by considering correlation of DE states among the cell types, both CeDAR methods improve the accuracy of csDE genes detection in all six cell types compared to the other methods (Fig. 3c and Additional file 1: Table S1). However, the amounts of improvement vary with respect to different factors, such as cell type proportion and sample size. The improvement in cell types with smaller proportions is greater than in cell types with larger proportions. For example, the improvement in cell type 1 (mean proportion 0.63) is much smaller than the other five cell types (largest mean proportion 0.11). Meanwhile, improvement in cell types with similar proportion could be different. Among the six cell types, cell type 2 and cell type 3 have similar mean proportion (0.10 vs. 0.11), but the accuracy improvement in cell type 2 is greater. A potential explanation is that cell type 2 is clustered with cell type 1 (with large proportion), while cell type 3 is clustered with cell types 4–6 (with smaller proportions). Intuitively, the cell type with small proportion could “borrow” more information from cell types with larger proportion, since larger proportion often leads to more accurate result.

Sample size is another important factor affecting the performance of various methods in detecting csDE genes, especially in cell types with small proportion [4, 5, 10]. When sample size is small (e.g., 50), both TOAST and TCA have poor performances in cell types of small proportions. However, the improvement of CeDAR methods is more significant compared to scenarios with larger sample size (Additional file 1: Table S1). For example, in cell type 2, the AUC-ROC difference between CeDAR-S and TOAST is 0.145 when sample size is 200, while it is 0.235 when the sample size is 50. Additionally, when sample size becomes large (e.g., 200), CeDAR-M has higher AUC-ROC than CeDAR-S in cell types with smaller proportions, such as cell type 2 (AUC-ROC: 0.940 vs. 0.916). This is because larger sample size would lead to more accurate multiple layer tree structure estimation, which helps cell types with smaller proportions to correctly “borrow” information from their closely correlated cell types with larger proportions.

We also investigated the FDR control of the four methods at a given cutoff. While TOAST, TCA, csSAM, and CellDMC use estimated FDR [21] 0.05 as cutoff, CeDAR methods use posterior probability of DE 0.95 as cutoff [22]. In general, all methods have better FDR control for cell types with larger proportions (Fig. 3d). For example, the median of observed FDR in cell type 1 is much closer to 0.05 and the interquartile range (IQR) is much smaller than cell type 6 for all four methods. In cell types with smaller proportion, TOAST, TCA, csSAM, and CellDMC have slightly better performance in controlling type I error than CeDAR. This indicates that the information borrowing across rare cell types tends to mildly inflate the false positives. But overall, all methods do not work well for cell types with small proportions, and the only solution for that is to increase the sample size. Such problem will be alleviated with larger sample size. For example, the observed FDR in cell type 6 from CeDAR-M decreases from 0.247 to 0.065 when sample size increases from 50 to 200 (Additional file 1: Table S1).

#### Evaluating the robustness of CeDAR

##### Robustness to different cell type correlation patterns

**Fig. 4:**
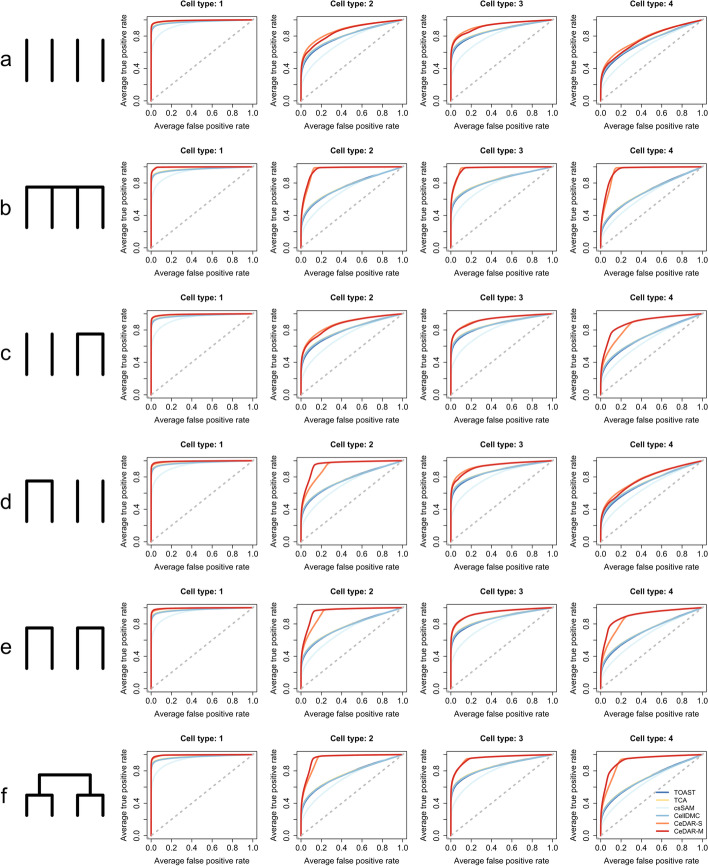
ROC curves under different DE patterns (with strong correlation). The simulation is conducted for a two-group comparison with four cell types (1: neutrophils, 2: monocytes, 3: CD4, 4: CD8 cells) under six different DE patterns (**a** all cell types are independent; **b** cell types are correlated under the root, but independent conditional on the root (a single layer tree structure); **c** only cell types 3 and 4 are correlated; **d** only cell types 1 and 2 are correlated; **e** cell types 1 and 2 are correlated, and cell types 3 and 4 are correlated, but cell types 1/2 and 3/4 are independent; **f** all cell types are correlated under a multiple-layer tree structure). Methods under comparison include TOAST, TCA, csSAM, CellDMC, CeDAR-S, and CeDAR-M. Reported ROC curves are average over 50 simulations

Due to the complexity of biological system, cell types may show different correlation patterns in their DE/DM status under different conditions. For example, some cell types may not show correlation with each other at all. To evaluate the robustness of CeDAR, we evaluated its performance under different cell type hierarchies. To simplify the simulation but still capture the influences of cell type hierarchy, we simulated data for four cell types (neutrophils, monocytes, CD4, and CD8) with different mean proportions (0.6, 0.1, 0.25, 0.05). We evaluated CeDAR methods with six different cell type hierarchies representing various correlation relationships (Fig. 4a–f). For hierarchies showing cell type correlation, we evaluated the performance of six methods under two different correlation levels (strong: ~90% DE genes overlapped between two cell types; weak: ~ 50%). Sample size was set as 200 per group.

The simulation results indicate that when all cell types are independent, CeDAR methods have similar accuracy as TOAST, TCA, and CellDMC and greater accuracy than csSAM in all four cell types (Fig. 4a). When cell types are strongly correlated, both CeDAR methods have greater improvements over the other methods in cell types with smaller proportions (e.g., cell type 2 in Fig. 4b, d, e; cell type 4 in Fig. 4b, c, e). However, such improvement is not as significant in cell type 1 under all scenarios. This is because cell type 1 has large proportion (mean 0.63) so the data likelihood plays a greater role than prior information; thus, borrowing information from other cell types does not much impact on the result. Additionally, CeDAR-M provides greater performance improvement than CeDAR-S when the cell type hierarchy is more complex than a one-layer tree structure (e.g., cell type 2 in Fig. 4d, e; cell type 4 in Fig. 4c, e). When correlation is weaker, CeDAR-M has similar performance as CeDAR-S, but the improvement over existing methods (TOAST, TCA, csSAM, and CellDMC) is smaller (Additional file 1: Figure S3, Table S3). The FDR control result is similar to the simulation result with six cell types in previous section regardless of different cell type hierarchies (Additional file 1: Figure S2, Figure S4, Table S2, Table S3).

##### Robustness to cell type hierarchy estimation

In many cases, the cell type hierarchy and/or the prior probabilities of nodes are unknown and need to be estimated from data. We conducted additional simulations to evaluate the impacts of potential estimation biases on CeDAR. We used the same simulation setting as the first simulation result section (six cell types, 100 samples per group) and compared the performance of csDE detection with different combinations of inputs: true tree and true prior probability, true tree and estimated prior probability, estimated tree and estimated prior probability. The result shows that using estimated tree structure and prior probabilities of nodes have very similar accuracies as the other two types of inputs in most cases (Additional file 1: Figure S5, Table S4). The only exception is cell type 2, where the performance is slightly worse by using estimated tree and probability. On the other hand, the observed FDRs from using estimated prior probability as input are closer to the nominal value (0.05) than using true prior portability. Further investigation suggests that the difference in FDR between using true and estimated prior probabilities is associated with data noise. When data noise is large, CeDAR with estimated prior probability has smaller FDR; otherwise, it has larger FDR (Table S5, Table S6). More details are provided in Additional file 1 Section S6.

We further evaluated CeDAR’s performance with mis-specified tree structures, which will happen when the tree estimation is inaccurate. We provided mis-specified tree structures to CeDAR and compared the results with CeDAR using the true tree. The results show that CeDAR is robust to mis-specified tree structures and that the major performance decreasing appears in low abundant cell types when they are mistakenly clustered with other cell types. Detailed procedures and discussions are provided in Additional file 1 Section S7 and Figure S6, Figure S7, Table S7. Overall, CeDAR is very robust to potential biases brought by the cell type hierarchy estimation.

##### Robustness to cell type proportion estimation

Although we assumed accurate proportion estimation in previous simulations, the estimation accuracy varies by the data quality and the choice of deconvolution methods. We evaluated the performance of the six methods under the same simulation scenario using estimated proportions from a reference-based deconvolution (RB) method *lsfit* [23] (Additional file 1: Figure S8, Table S8). As expected, using true proportion leads to better results for all methods, especially in low abundant cell types (cell type 3-6). However, these results show that using the estimated proportions, CeDAR methods still have much higher accuracies than the other four methods in all cell types. Another observation is that the observed FDRs from all methods are inflated using estimated proportions. We took a deeper examination of the results and found that the estimated proportions are more variable across individuals compared to the true proportions. Such higher variability makes all methods more sensitive (since proportions are used in the linear model as covariates), but also produces more false positives. The obvious solution to this problem is to have better proportion estimation, or to use a more stringent cutoff in calling csDE/csDM. Overall, these results show that CeDAR still greatly outperforms other methods using estimated cell type proportions.

#### Computation performance

We benchmarked the computation performance of CeDAR and other methods under the simulation scenario in the first simulation result section (12,402 genes), but varying the cell type number (4, 6, and 8) and sample size (50, 100, and 200). All simulations were performed on a PC running Linux with 2.80 GHz CPU and 8G RAM. TOAST is the fastest and CellDMC is the second fastest method. For example, they take 0.409 and 24.466 s respectively for 6 cell types and 100 samples on average. With default permutation number of 200, csSAM is slower than CeDAR-M with four cell types (sample sizes 50, 100, 200) and six cell types (sample sizes 50, 100), while it is faster with six cell types (sample sizes 200). TCA is the slowest in all scenarios. Overall, even though with *K* cell types, CeDAR needs to fit 2^*K*^ linear regression models, its computation performance is still very good due to efficient implementation. For example, it takes about 36.759 s for 6 cell types and 100 samples per group. Computation time for all scenarios is in Additional file 1: Table S9.

### Real data analysis

#### Cell type-specific differential methylation in brain

**Fig. 5:**
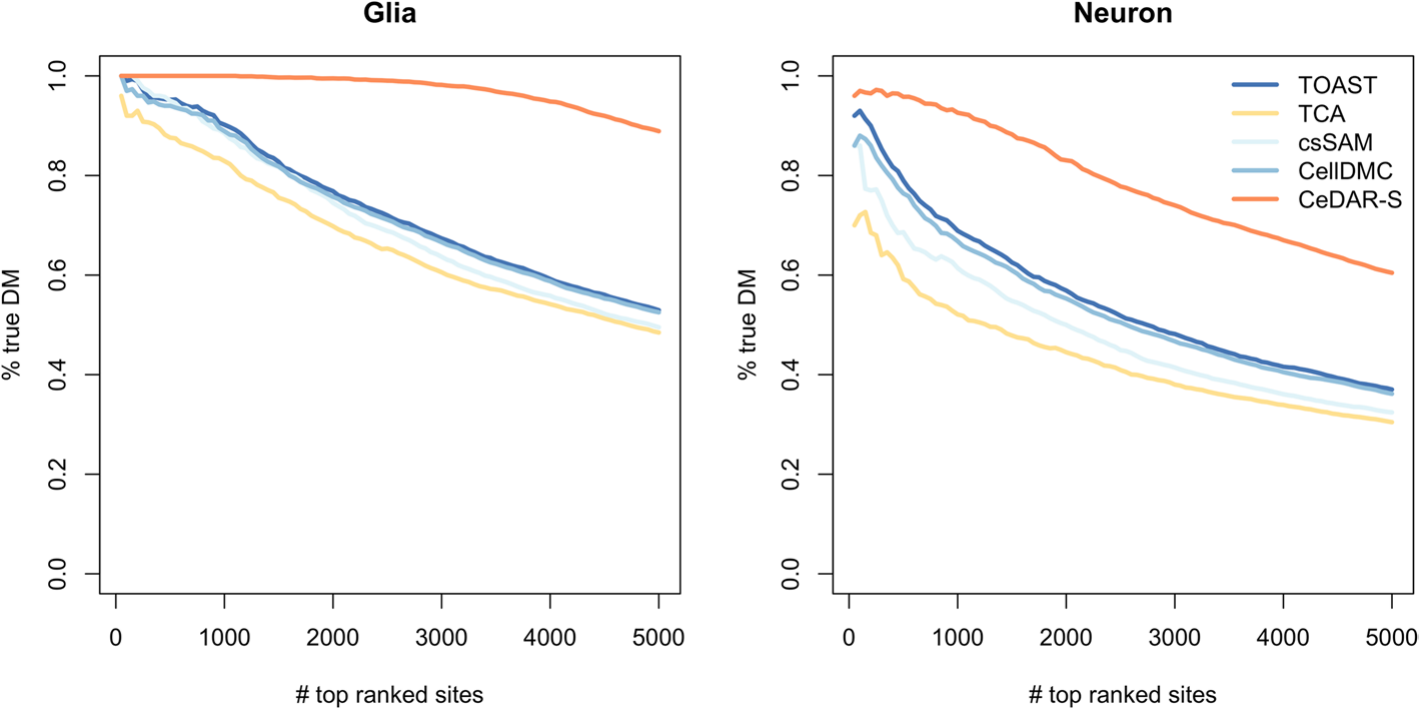
Accuracy of detecting csDM in human brain methylation data. The human brain DNA methylation dataset (GEO accession number: GSE41826) contains both bulk samples from postmortem frontal cortex and matched cell type samples of neuron and glia purified by fluorescence-activated cell sorting (FACS). The csDM sites associated with sex were identified between five healthy male and five healthy female samples with TOAST, TCA, csSAM, CellDMC, and CeDAR-S. The results are evaluated by the true discovery rate (TDR) curves, which show the accuracy among different numbers of top-ranked csDM sites from each method.

We first evaluated CeDAR on a human brain DNA methylation dataset (GEO accession number GSE41826 [24]) including both pure (glia and neuron) and bulk samples from 5 males and 5 females. The methylation level is represented as beta values in this study and all following DNA methylation analyses. We applied CeDAR-S, TOAST, TCA, csSAM, and CellDMC on the bulk data to call glia and neuron-specific differentially methylated CpGs (DMCs) comparing male vs female and used the DMCs identified from the pure cell type as the gold standard to benchmark the results. The gold standard cell type-specific DMCs were detected using *minfi* [25–31]. To obtain an accurate gold standard and avoid ambiguity in DM calling, we defined sites with FDR < 0.01 as DM and FDR > 0.8 as non-DM. Among all 480,492 CpGs, there were 8475 and 8587 true DM sites identified in glia and neuron respectively. The two cell types share 7622 common true DM sites, indicating a strong correlation between cell types. The true DM and non-DM sites are then used to evaluate the csDM called from bulk samples. The estimated mixture proportions (by RB deconvolution) and the whole-tissue DNA methylation data were used as inputs for TOAST, TCA, csSAM, CellDMC, and CeDAR-S. Accuracy was measured by true discovery rate (TDR) in top ranked sites. The TDR curves in Fig. 5 show that CeDAR-S has significantly higher accuracy among the top CpG sites than the other methods in both glia and neuron. For example, in glia, the difference of TDR between CeDAR-S and TOAST among top-ranked 5000 sites is more than 30%.

#### Cell type-specific differential methylation in whole blood

**Fig. 6:**
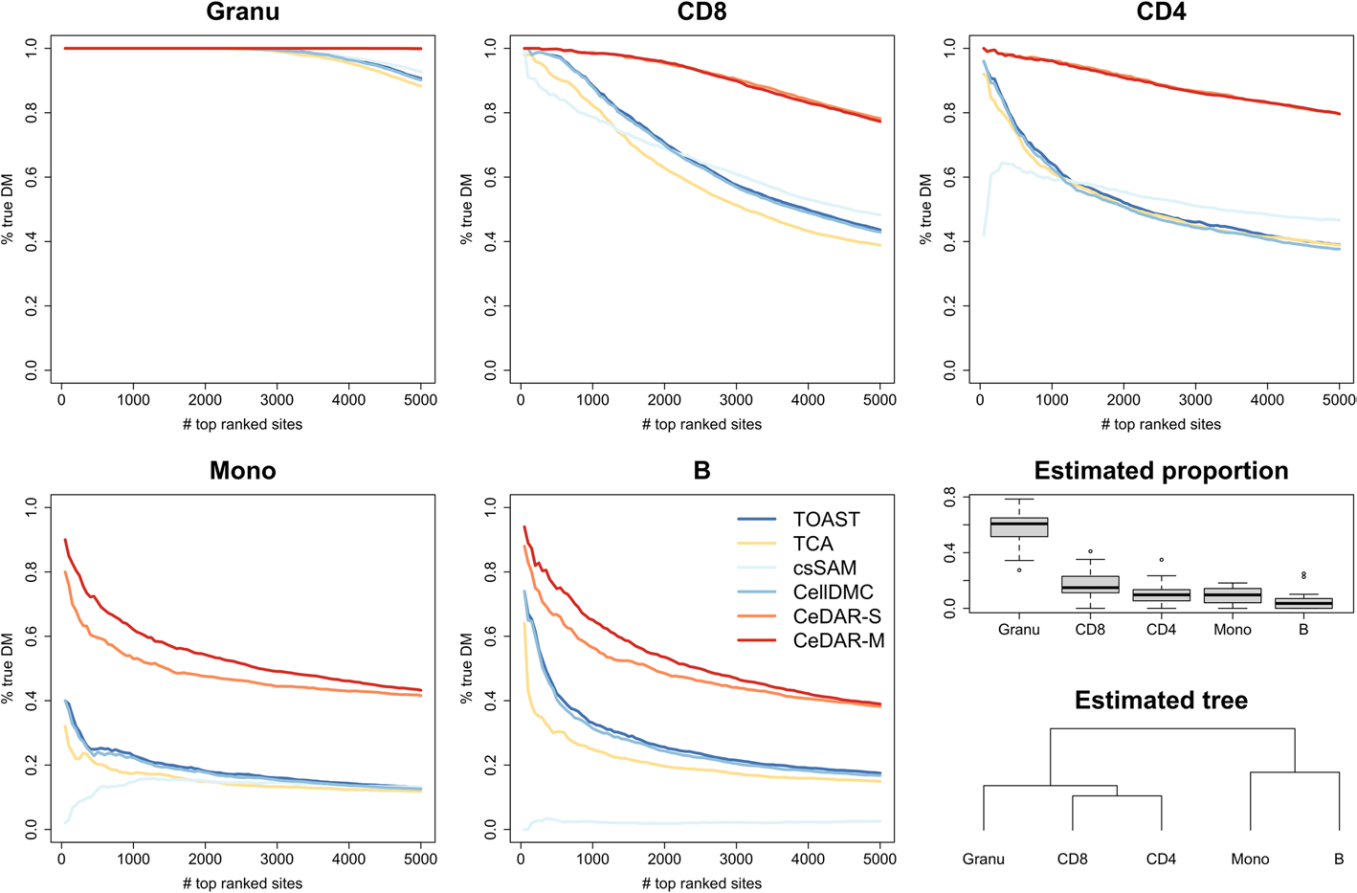
Accuracy of detecting csDM in human whole blood methylation data. The human blood DNA methylation dataset (GEO accession number: GSE166844) contains both bulk samples from whole blood and pure cell type samples of granulocytes, CD8, CD4, monocytes, and B cells derived by FACS. The csDM sites associated with sex were identified between eighteen females and twelve males samples using TOAST, TCA, csSAM, CellDMC, CeDAR-S, and CeDAR-M. The results are evaluated by TDR curves. The estimated proportions and estimated tree structure of cell types are shown in the last panel

We further evaluated CeDAR on another set of human blood DNA methylation data (GEO accession number GSE166844 [17]), which contains the profiles of five pure cell types (CD4, CD8, B, monocytes, and granulocytes) and the whole blood samples from 30 individuals (18 females vs. 12 males). We performed cell type-specific differential methylation analyses in the bulk data for male-female comparison. Since there are more cell types in this dataset, we can create a hierarchical tree on the cell types, which allows us to compare CeDAR-M and CeDAR-S. We again defined the gold standard csDM using the pure cell type methylation between males and females by FDR *<* 0*.*01; non-DM by FDR *>* 0*.*8. There were 27,219 (CD4), 11,155 (CD8), 10,482 (B), 11,325 (monocytes), and 13,938 (granulocytes) DM sites identified. The number of overlapped true DM sites among cell types is shown in Additional file 1: Figure S9. Again, there are significant overlaps of DMCs in different cell types. The TDR curves for top-ranked csDM sites detected from different methods are shown in Fig. 6. Both CeDAR-M and CeDAR-S have higher accuracies among the top CpG sites than the other four methods in all five cell types. For granulocytes (with the largest proportion), all methods have perfect accuracies in top 2000 ranked sites. However, the TDRs of TOAST, TCA, csSAM, and CellDMC in top 5000 sites drop to 90%, while the TDRs of two CeDAR methods are still close to 1, indicating a performance improvement. In cell types with relative smaller proportions (CD8, CD4, monocytes, and B cells), all methods have worse performance, but CeDAR methods still have much higher TDR than the other methods and the performance improvement is even greater. Additionally, for monocytes and B cells, CeDAR-M method has higher accuracy than CeDAR-S, since both have small proportions and are clustered together. This suggests that incorporating a detailed tree structure makes information sharing more efficient.

#### Cell type-specific differential methylation in rheumatoid arthritis study

Previous two real datasets provide pure cell type data to serve as gold standard. However, the analyses were performed on a rather simple setting: detecting csDM between males and females without other covariates. To fully evaluate CeDAR performance in a more complex experimental design, we analyzed another dataset that provides peripheral blood leukocytes (PBL) DNA methylation from 332 normal individuals and 354 rheumatoid arthritis (RA) patients (GEO accession number GSE42861 [32]). After preprocessing, we performed cell type-specific analyses by comparing different disease statuses (RA vs. control), treating age as a cell type-specific confounder and smoking status and sex as main-effect confounders. This design contains different types of variables (categorical disease status and continuous age) with potential cell type-specific effects, and other covariates without cell type-specific effects. This analysis showcases the flexibility of CeDAR. All data analysis settings are the same for the six methods except the threshold to call DMC. For TOAST, TCA, csSAM, and CellDMC, sites with FDR <0.05 were reported as csDMCs; for CeDAR-S and CeDAR-M, sites with posterior probability of DM > 0.95 were reported as csDMCs.

**Fig. 7:**
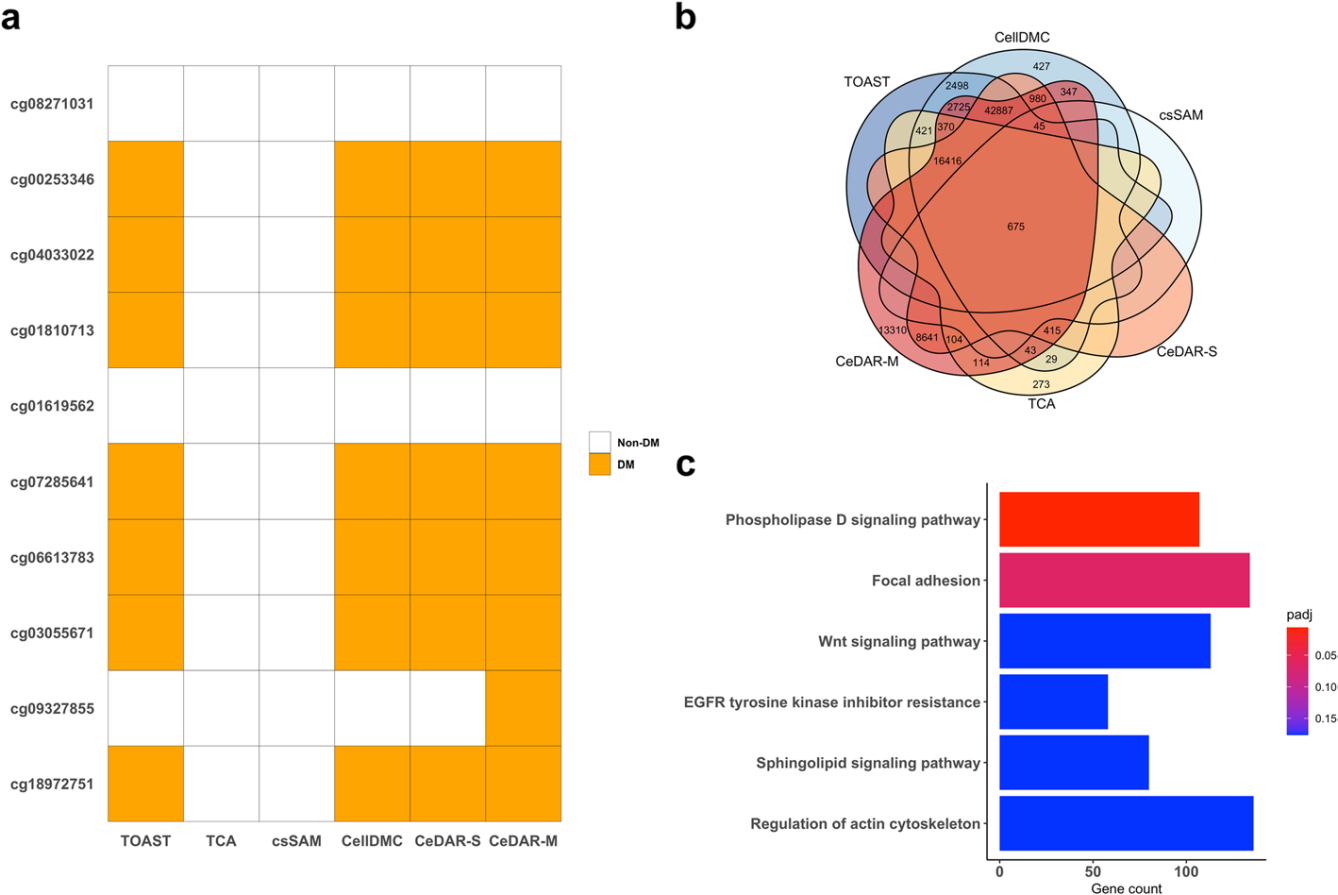
Cell type-specific DMC result for PBL DNA methylation data between RA and normal individuals. **a** Examination of six methods in identifying csDMCs of B cells from Liu et. al [32]. The ten csDMCs were identified and validated in two independent cohorts [36]. **b** Venn diagram showing overlap of reported csDMCs in CD4 cell type from six methods. **c** Top six KEGG pathways enriched by CeDAR-M uniquely identified csDMCs in CD4, but not TCA and TOAST

B cell plays an important role in RA [33–35]. From purified B cells, Julia et al. identified ten RA-related DMCs validated in two independent EWAS cohorts (UK and Spain) [36]. We examined whether the six methods could detect those ten DMCs in B cells from the PBL DNA methylation bulk data. As can be seen from Fig. 7a, TCA and csSAM did not report any site out of the ten in B cells; TOAST, CellDMC, and CeDAR-S identified seven of them; and CeDAR-M identified eight sites. CD4 is another cell type reported to be related to RA [37, 38]. However, there is no experimentally validated DMCs in CD4. To investigate whether the csDMCs detected for CD4 from CeDAR make biological and clinical sense, we performed a series of analyses to evaluate the results. First, Fig. 7b shows a Venn diagram for the overlaps of the reported csDMCs in CD4 by the six methods. We see that CeDAR-M detected much more csDMCs in CD4 that include all csDMCs from CeDAR-S, and a large proportion of csDMCs from other four methods. Furthermore, we performed an enrichment analysis for the csDMCs uniquely identified by CeDAR-M, but not by TOAST, TCA, csSAM, and CellDMC, using *missMethyl* [39]. There are six KEGG pathways [40–42] significantly enriched (two with adjusted *p*-value < 0.1 and four with adjusted *p*-value < 0.2). The top one, Phospholipase D signaling pathway, has been reported to play a pivotal role in RA. Previous studies showed that abnormal upregulation of a gene in Phospholipase D signaling pathway, Phospholipase D1 (PLD1), may contribute to the pathogenesis of IL-1β-induced chronic arthritis [43]. Additionally, genetic and pharmacological inhibition of PLD1 can cause suppression of collagen-induced arthritis symptom, such as induction of the inflammatory response, bone destruction, and osteoclastogenesis [44]. The other five pathways are focal adhesion, Wnt signaling pathway, EGFR tyrosine kinase inhibitor resistance, Sphingolipid signaling pathway, and regulation of actin cytoskeleton, which are also reported being related with RA disease [45–49]. We further investigated whether these six enriched KEGG pathways can be also identified by other competing methods (Table 1). We found that among the six pathways, Sphingolipid signaling pathway is uniquely identified by CeDAR. TOAST reports the remaining five other pathways, while TCA, CellDMC, and csSAM report fewer pathways. This result indicates that CeDAR can find unique csDMCs, leading to pathways and biological interpretations related to target phenotype that other methods cannot provide.

**Table 1:** Identification of CeDAR-enriched pathways by TOAST, TCA, CellDMC, and csSAM

Pathways reported in Fig. **7**c	CeDAR	TOAST	TCA	CellDMC	csSAM
Phospholipase D signaling pathway	Yes	Yes	Yes	Yes	No
Wnt signaling pathway	Yes	Yes	No	Yes	No
Focal adhesion	Yes	Yes	Yes	Yes	No
EGFR tyrosine kinase inhibitor resistance	Yes	Yes	No	No	No
Sphingolipid signaling pathway	Yes	No	No	No	No
Regulation of actin cytoskeleton	Yes	Yes	No	Yes	No

There are six enriched KEGG pathways (with adjusted *p*-value < 0.2) based on CeDAR uniquely identified csDMCs. We check whether they can be identified by performing the same enrichment analysis on csDMCs identified by TOAST, TCA, CellDMC, and csSAM. In the table, “Yes” means the pathway is enriched by csDMCs reported by corresponding method, while “No” means it is not.

### Other real data results

In additional to the above results, we analyzed several other real datasets: (1) detecting Down syndrome (DS)-associated csDM sites from frontal cortex gray matter samples (GSE74886 [50]); (2) detecting systemic lupus erythematosus (SLE)-associated csDM sites from whole blood samples (GSE118114 [51]); (3) detecting smoking-associated csDM sites from whole blood samples in two independent studies separately (GSE42861 and GSE402079 [52]). All the three results demonstrate that CeDAR methods can achieve much more accurate results than other methods. The details of the analysis procedure and results are provided in the Additional file 1 Section S8 and Figure S10 – S12.

Taken together from the real data analysis results, we conclude that the proposed methods are more accurate and sensitive compared to the existing methods. Particularly, CeDAR-M demonstrates better results compared to CeDAR-S and the results from CeDAR-M can potentially provide more biologically plausible target for future studies.

## Discussion

In this work, we developed a novel statistical model called “CeDAR” that incorporates the cell type hierarchy in the cell type-specific differential analysis. The model is inspired by real data observation that cell types show strong correlation in their DE/DM states. CeDAR is based on a Bayesian hierarchical model incorporating the cell type hierarchy in the construction of prior probabilities for DE/DM. We derived procedures for parameter estimation and used the posterior probabilities for determining features’ differential states. Extensive simulation studies and real data analyses demonstrate that CeDAR significantly improves the sensitivity and accuracy in identifying csDE/csDM compared to existing methods, especially for cell types with low proportions.

We showed that the performance improvement of CeDAR is robust to the specification of cell type hierarchy, for example, when the true structure is not bifurcating or just has a single layer. Even when the cell types are completely independent, CeDAR is not worse than other methods. When the correlation between cell types is strong, CeDAR-M is recommended, since it can capture a complex cell type hierarchy; when the correlation is weak or sample size is small, CeDAR-S is preferred, because it can capture a certain level correlation without the need for the complex tree structure estimation. We also showed that the biases in the cell type hierarchy and cell type proportion estimation may impact the results, but the improvements over other methods are still significant. On the other hand, accurate hierarchy and proportion estimation will lead to better results. With the increasing availability of single-cell genomics data, we envision that such estimation will become more accurate for many biological systems, which will greatly benefit cell type-specific analyses in bulk data.

In this work, we implicitly assumed that the correlations among cell types are consistent for all features. However, in the real world, cell types may show different correlation patterns in DE/DM states among different feature sets corresponding to different biological processes. Thus, a more sophisticated method is to assume cell types have different correlations in different feature sets, which will be our future research direction. Additionally, CeDAR method is currently designed for continuous data, such as gene expression or DNA methylation microarray data. However, the general framework of borrowing information from cell types can be applied to other data types, such as the count data from sequencing. This is another promising future direction for us to explore.

## Conclusion

Cell type-specific differential analysis plays an important role in uncovering biological mechanism and finding biomarkers in biological or clinical studies. While single-cell sequencing or cell sorting techniques can be too expensive to be applied in large-scale studies, computational deconvolution from bulk data is a promising method for such analysis. In this work, we developed a novel statistical method named CeDAR to incorporate cell type hierarchy in the cell type-specific differential analysis. Both simulation and real data analysis demonstrate that CeDAR significantly improves csDE/csDM detection accuracy and power, especially in low-abundance cell types. It is also robust to various correlation patterns of DE/DM status among cell types. We expect that CeDAR will better exploit the bulk omics data and extract more meaningful information.

## Methods

### The CeDAR method

#### Data model

Suppose the data was generated from a bulk high-throughput experiment, which contains measurement of *G* features (genes, CpG sites, etc.) in *N* samples. Let *Y*_*gi*_ represent the observed measurement of *g*th feature in *i*th sample. In each sample, the measurement of each feature is a mixed signal from *K* different cell types. Let ***θ***_***i***_ = (*θ*_*i*1_, …, *θ*_*iK*_)^*T*^ represent the cell composition of the *i*th sample. There are several methods for estimating *K* and ***θ***_***i***_ in both DNA methylation and gene expression data [53–55]. Here we assume both *K* and ***θ***_***i***_ are known. We assume there are *Q* confounders to be adjusted in the study. Let ***C***_***i***_ = (*C*_*i*1_, …, *C*_*iQ*_)^*T*^ represent the confounders of *i*th sample. Then ***C*** = (***C***_**1**_, …, ***C***_***N***_)_*Q* × *N*_ represents the confounders of all samples. Let ***A*** = (***A***_**1**_, …, ***A***_***N***_) represent the factor to be tested for cell type-specific effects across all *N* samples. ***A***_***i***_ is a scaler if a single continuous or binary factor is involved; if the factor is a categorical variable with multiple levels, it will be coded as a vector of dummy variables.

Now consider the csDE/csDM status for a particular covariate of interest. For the simplicity of notation, we will omit the subscript for covariate. The model described below will be applied to all covariates of interest. Define *Z*_*gk*_ as a binary random variable to represent the DE/DM state of the *g*th feature in *k*th cell type. When *Z*_*gk*_ = 1, the *g*th feature in *k*th cell type is DE/DM associated with the factor of interest, and *Z*_*gk*_ = 0 otherwise. Note that since ***Z***_***g***_ = (*Z*_*g*1_, …, *Z*_*gK*_) takes value in discrete space {0, 1}^*K*^, there are 2^*K*^ combinations of DE/DM states for *K* cell types. Let *X*_*gik*_ represent the unknown pure profile of feature *g* in cell type *k* for sample *i*. We assume that given all DE/DM state of feature *g* in cell type *k*, it satisfies $E\left[{X}_{gik}|{Z}_{gk}\right]={\mu}_{gk}+\boldsymbol{C}_{\boldsymbol{i}}^{\boldsymbol{T}}\boldsymbol{\beta}_{\boldsymbol{gk}}+{Z}_{gk}\boldsymbol{A}_{\boldsymbol{i}}^{\boldsymbol{T}}\boldsymbol{\delta}_{\boldsymbol{gk}}$. Here *μ*_*gk*_ is the baseline profile of feature *g* for cell type *k*; ***β***_***gk***_ = (*β*_*gk*1_, …, *β*_*gkQ*_)^***T***^ are coefficients associated with confounders, and ***δ***_***gk***_ are coefficients associated with the factors of interest. Specifically, for any confounder without cell type-specific effect (*C*_*q*_), its corresponding coefficients in different cell types are the same (*β*_*g*1*q*_ = *β*_*g*2*q*_ = … = *β*_*gKq*_). It is important to note here that the factors of interest only impact on *X*_*gik*_ when *Z*_*gk*_ = 1 (the cell type is DE/DM). This is a major modeling difference from all other linear model-based cell type-specific methods (TOAST, CellDMC, TCA, etc.), which would always include the impact of ***A***. For the observed bulk data, since they are mixtures of cell type-specific signals, the observed measurement *Y*_*gi*_ is a weighted average of *X*_*gik*_’s: *E*[*Y*_*gi*_; ***θ***_***i***_] = ∑_*k*_*θ*_*ik*_*E*[*X*_*gik*_]. Thus, given the DE/DM state in *K* cell types ***Z***_***g***_, *Y*_*gi*_ satisfies the following linear form:

1 $$E\left[{Y}_{gi}|\  \boldsymbol{Z}_{\boldsymbol{g}}\right]=\sum_{k=1}^K{\theta}_{ik}\left({\mu}_{gk}+\boldsymbol{C}_{\boldsymbol{i}}^{\boldsymbol{T}}\boldsymbol{\beta}_{\boldsymbol{gk}}+{Z}_{gk}\boldsymbol{A}_{\boldsymbol{i}}^{\boldsymbol{T}}\boldsymbol{\delta}_{\boldsymbol{gk}}\right)$$

Since the interactions between mixing proportion and factor of interest are only allowed for cell types showing DE/DM state (e.g., cell type *k* with *Z*_*gk*_ = 1), the linear model used in existing methods such as TOAST and CellDMC is a special case in which all cell types are assumed to be DE/DM *a priori* (the full model).

Given the data model, we can obtain the observed data likelihood and derive the posterior probability for DE/DM calling. Denote ***Y***_***g***_ = (*Y*_*g*1_, …, *Y*_*gN*_), the goal of csDE/csDM calling is to compute *P*(*Z*_*gk*_ = 1|***Y***_***g***_). Of course, such posterior probability relies on the prior. In the next subsection, we provide a detailed explanation on how to construct priors based on cell type hierarchy to achieve information sharing.

#### Prior probabilities for the DE/DM states

As discussed before, a major methodological contribution of this work is the specification of csDE/csDM priors based on the cell type hierarchy. This plays a major role in capturing the similarity among cell types and improving the DE/DM calling result. For each feature, we define a list of binary random variables for the underlying DE/DM states for all nodes: ***Z*** for leaf nodes and ***D*** for non-leaf nodes. We assume these binary random variables are independent and identically distributed for all genes. We further assume that the cell type hierarchy is known at this step. The estimation of cell type hierarchy will be discussed in the later section.

The correlation in the hidden DE/DM states among cell types is captured by the joint probability of ***Z***_***g***_ and ***D***_***g***_. For *g* = 1, …, *G*, and *k* = 1, …, *K*, the DE/DM state of the leaf nodes is represented by binary random variables *Z*_*g*1_, …, *Z*_*gK*_, with *Z*_*gk*_ ∼ *Bernoulli*(*π*_*k*_). *Z*_*gk*_ = 1 means that the *g*th feature in *k*th cell type is DE/DM, and *Z*_*gk*_ = 0 otherwise. The states of all non-leaf nodes are also represented by binary random variables. Given a hierarchical tree of the cell types, the state for the *n*th node at *l*th level (*l* = 1, …, *L*; *n* = 1, …, *n*_*l*_) of the tree is denoted by binary random variable ${D}_{g{\Phi}_{l,n}}$, where Φ_*l*, *n*_ is a set of cell types represented by corresponding descendant leaf nodes. Specifically, the root node is defined as the first node at level 0, denoted as ${D}_{g{\Phi}_{0,1}}$. We assume ${D}_{g{\Phi}_{l,n}}\sim Bernoulli\left({\pi}_{\Phi_{l,n}}\right)$. To capture the tree structure, we define that for any non-root node (internal or leaf): if its parent node has state 0, it must have state 0; if the parent node has state 1, its state follows a Bernoulli distribution. Thus, the conditional distribution for the states of the leaf nodes can be expressed as the following, where ${D}_{g{\Phi}_{l,n}}$ is the parent node of *Z*_*gk*_:

2 $${Z}_{gk} \mid {D}_{g{\Phi}_{l,n}}\sim Bernoulli\left({p}_k{D}_{g{\Phi}_{l,n}}\right)$$

Here, ${p}_k=\frac{\pi_k}{\pi_{\Phi_{l,n}}}$. Distributions for the non-leaf internal nodes can be expressed in a similar form, that is, the state of a child internal node condition on the state of its parent follows a Bernoulli distribution. Finally, we assume that the sibling nodes are mutually independent if their parent node has state 1.

The specification of the prior probabilities captures the similarity among cell types according to the cell type hierarchy. Using the structure in Fig. 2 as an example, there are three leaf nodes with underlying states represented by *Z*_*g*1_, *Z*_*g*2_, *Z*_*g*3_, and two non-leaf nodes represented by *D*_*g*{1, 2, 3}_, *D*_*g*{2, 3}_. The marginal prior probabilities of a randomly picked feature being DE/DM in cell types 2 and 3 are *P*(*Z*_*g*2_ = 1) = *P*(*Z*_*g*2_ = 1| *D*_*g*{2, 3}_ = 1) × *P*(*D*_*g*{2, 3}_ = 1| *D*_*g*{1, 2, 3}_ = 1) × *P*(*D*_*g*{1, 2, 3}_ = 1) = *p*_2_ × *p*_{2, 3}_ × *π*_{1, 2, 3}_ = 0.06 and *P*(*Z*_*g*3_ = 1) = *P*(*Z*_*g*3_ = 1| *D*_*g*{2, 3}_ = 1) × *P*(*D*_*g*{2, 3}_ = 1| *D*_*g*{1, 2, 3}_ = 1) × *P*(*D*_*g*{1, 2, 3}_ = 1) = *p*_3_ × *p*_{2, 3}_ × *π*_{1, 2, 3}_ = 0.04, respectively. The marginal joint probability of a randomly picked feature being DE/DM in both cell type 2 and cell type 3 is *P*(*Z*_*g*2_ = *Z*_*g*3_ = 1) = *p*_2_ × *p*_3_ × *p*_{2, 3}_ × *π*_{1, 2, 3}_ = 0.03. It is much larger than *P*(*Z*_*g*2_ = 1) × *P*(*Z*_*g*3_ = 1) = 0.0024, which is the probability assuming cell types 2 and 3 are independent. If the root node always has state 1, i.e., *P*(*D*_*g*{1, 2, 3}_ = 1) = 1, then cell type 1 will be independent of cell types 2 and 3. Furthermore, if *P*(*D*_*g*{1, 2, 3}_ = 1) = *P*(*D*_*g*{2, 3}_ = 1) = 1, then the three cell types are mutually independent. Importantly, such cell type hierarchy is used merely as a statistical way to capture DE/DM state correlations among cell types. It does not necessarily represent the cell type lineage tree during differentiation or development.

We use *Parent*() to represent the parent node of a specific node. Then, a prior joint probability of ***Z***_***g***_ = (*Z*_*g*1_, …, *Z*_*gK*_) and $\boldsymbol{D}_{\boldsymbol{g}}=\left({D}_{g{\Phi}_{0,1}},\dots, {D}_{g{\Phi}_{L,{n}_L}}\right)$ has the following form:

3 $$\begin{array}{c}\begin{array}{rcl}P\left({\boldsymbol Z}_{\boldsymbol g},{\boldsymbol D}_{\boldsymbol g}\right)&=&P\left({\boldsymbol Z}_{\boldsymbol g}\vert{\boldsymbol D}_{\boldsymbol g}\right)\times P\left({\boldsymbol D}_{\boldsymbol g}\right)\\&=&\left[\prod_{k=1}^KP\left(Z_{gk}\vert Parent\left(Z_{gk}\right)\right)\right]\times\left[\prod_{l=1}^L\prod_{n=1}^{n_l}P\left(D_{g\Phi_{l,n}}\vert Parent\left(D_{g\Phi_{l,n}}\right)\right)\right]\times P\left(D_{g\Phi_{0,1}}\right)\end{array}\\ \kern-5.2em=\kern.5em\left(\prod_{k=1}^K\left\{\left[p_kParent\left(Z_{gk}\right)\right]^{Z_{gk}}\left[1-p_kParent\left(Z_{gk}\right)\right]^{1-Z_{gk}}\right\}\right)\times\\\begin{array}{c}\kern4.5em\left(\prod_{l=1}^L\prod_{n=1}^{n_l}\left\{\left[p_{\Phi_{\text{l},\text{n}}}Parent\left(D_{g\Phi_{l,n}}\right)\right]^{D_{g\Phi_{l,n}}}\left[1-p_{\Phi_{l,n}}Parent\left(D_{g\Phi_{l,n}}\right)\right]^{1-D_{g\Phi_{l,n}}}\right\}\right)\times\\ \kern-14.6em\left[\pi_{\Phi_{0,1}}^{D_{g\Phi_{0,1}}}\left(1-\pi_{\Phi_{0,1}}\right)^{1-D_{g\Phi_{0,1}}}\right]\end{array}\end{array}$$

#### Likelihood and posterior probability

Given the data model and the prior probabilities, we are now in position to derive the posterior probability for DE/DM calling. Denote ***Y***_***g***_ = (*Y*_*g*1_, …, *Y*_*gN*_), the probability of ***Y***_***g***_ given ***Z***_***g***_ is:

4 $$P\left({\boldsymbol{Y}}_{\boldsymbol{g}}|{\boldsymbol{Z}}_{\boldsymbol{g}}\right)={\prod}_{i=1}^NP\left({Y}_{gi}|\boldsymbol{Z}_{\boldsymbol{g}}\right)$$

The joint probability of ***Y***_***g***_, ***Z***_***g***_, ***D***_***g***_ can be derived as the following, noting that *P*(***Y***_***g***_| ***Z***_***g***_, ***D***_***g***_) = *P*(***Y***_***g***_| ***Z***_***g***_)

5 $$P\left(\boldsymbol{Y}_{\boldsymbol{g}},\boldsymbol{Z}_{\boldsymbol{g}},\boldsymbol{D}_{\boldsymbol{g}}\right)=P\left(\boldsymbol{Y}_{\boldsymbol{g}}|\boldsymbol{Z}_{\boldsymbol{g}}\right)\times P\left(\boldsymbol{Z}_{\boldsymbol{g}},\boldsymbol{D}_{\boldsymbol{g}}\right)=\left({\prod}_{i=1}^NP\left({Y}_{gi}|\boldsymbol{Z}_{\boldsymbol{g}}\right)\right)\times P\left(\boldsymbol{Z}_{\boldsymbol{g}},\boldsymbol{D}_{\boldsymbol{g}}\right)$$

Then, we can have the marginal probability for the observed data *P*(***Y***_***g***_) by summing over all combinations of (***Z***_***g***_, ***D***_***g***_):

6 $$P\left(\boldsymbol{Y}_{\boldsymbol{g}}\right)=\sum_{\left(\boldsymbol{Z}_{\boldsymbol{g}},\boldsymbol{D}_{\boldsymbol{g}}\right)}P\left(\boldsymbol{Y}_{\boldsymbol{g}},\boldsymbol{Z}_{\boldsymbol{g}},\boldsymbol{D}_{\boldsymbol{g}}\right)$$

Similarly, the joint probability of *Z*_*gk*_ = 1 and ***Y***_***g***_ is:

7 $$P\left({Z}_{gk}=1,\boldsymbol{Y}_{\boldsymbol{g}}\right)=\sum_{\left(\boldsymbol{Z}_{\boldsymbol{g}},\boldsymbol{D}_{\boldsymbol{g}}\right)}P\left(\boldsymbol{Y}_{\boldsymbol{g}},\boldsymbol{Z}_{\boldsymbol{g}},\boldsymbol{D}_{\boldsymbol{g}}\right)\times I\left({Z}_{gk}=1\right)$$

Based on these, we have the posterior probability of *Z*_*gk*_ = 1 conditional on ***Y***_***g***_ as:

8 $$P\left({Z}_{gk}=1|\boldsymbol{Y}_{\boldsymbol{g}}\right)=\frac{\sum_{\left(\boldsymbol{Z}_{\boldsymbol{g}},\boldsymbol{D}_{\boldsymbol{g}}\right)}P\left(\boldsymbol{Y}_{\boldsymbol{g}},\boldsymbol{Z}_{\boldsymbol{g}},\boldsymbol{D}_{\boldsymbol{g}}\right)\times I\left({Z}_{gk}=1\right)}{\sum_{\left(\boldsymbol{Z}_{\boldsymbol{g}},\boldsymbol{D}_{\boldsymbol{g}}\right)}P\left(\boldsymbol{Y}_{\boldsymbol{g}},\boldsymbol{Z}_{\boldsymbol{g}},\boldsymbol{D}_{\boldsymbol{g}}\right)}$$

The joint prior *P*(***Z***_***g***_, ***D***_***g***_) derived from Eq. (3) can be plugged into Eq. (5) to obtain *P*(***Y***_***g***_, ***Z***_***g***_, ***D***_***g***_), and then the posterior probabilities can be calculated for csDE/csDM calling. For all above, we have not made any distribution assumption on the data. For microarray data, we use normal distributions for the observed data. The same principles apply for other data types with different distribution assumptions.

### Parameter estimation

To derive the posterior probability of *Z*_*gk*_ (Eq. 8), we need to estimate the cell type hierarchy capturing cells correlation in DE/DM state, the prior probabilities of all nodes in the tree, and the marginal likelihood given different combinations of DE/DM states.

#### Estimation of the cell type hierarchy

The tree structure describing cell type hierarchy could be estimated by hierarchical clustering of cell types, in which the similarity between cell types is defined based on the Pearson correlation of *p*-values with the following form:

9 $$similarity\left(k,{k}^{\prime}\right)=\frac{1}{2}\left[\ 1- cor\left(-{\log}10\left( \boldsymbol{pval}_{\boldsymbol{k}}\right),-{\log}10\left( \boldsymbol{pval}_{\boldsymbol{k\prime}}\right)\right)\right]$$

***pval***_***k***_ are *p*-values generated by TOAST for testing differential signal in *k*th cell type of features satisfying {feature *g* : for 1 ≤ *g* ≤ *G*, ∃*k* ∈ {1, …, *K*} *s*. *t*. *pval*_*gk*_ (*or fdr*_*gk*_) < *threshold*}. This step is designed to reduce noise signal from non-DE/non-DM features. The threshold could be arbitrarily defined by users. Users could even define their own rule to select features for estimating the tree structure. Cell types with higher correlations should be more similar.

We want to emphasize that the cell type hierarchy does not have to be a bifurcating tree. In our software implementation, a bifurcating tree will be estimated from the data by default, but users have the option to specify a tree structure according to their prior biological knowledge. In addition, we also have option for using a simplified cell type hierarchy, in which all cell types are assumed to be independent under the root node. We call this the “single-layer” model, where the correlations among cell types are only captured at the root level.

#### Estimation of the prior probabilities

Based on the *p*-values provided by TOAST, the prior probability for an internal node ${D}_{g{\Phi}_{l,n}}$to be DE/DM (${\pi}_{\Phi_{l,n}}$) is estimated as the proportion of features deemed significant in any cell type belonging to set Φ_*l*, *n*_ among all *G* features.

10 $${\hat{\pi}}_{\Phi_{l,n}}=\frac{\sum_{g=1}^GI\left(\underset{k\in {\Phi}_{l,n}}{\min}\left\{ pva{l}_{gk}\right\}< threshold\right)}{G}$$

Then the conditional probability of non-root internal node ${D}_{g{\Phi}_{l,n}}$ conditional on its parent node ${D}_{g{\Phi}_{l^{\prime },{n}^{\prime }}}$ equals to one (${p}_{\Phi_{l,n}}$) is simply estimated by plugging in corresponding estimates of marginal probabilities:

11 $${\hat{p}}_{\Phi_{l,n}}=\frac{{\hat{\pi}}_{\Phi_{l,n}}}{{\hat{\pi}}_{\Phi_{l^{\prime },{n}^{\prime }}}}$$

Prior probabilities of leaf node *Z*_*gk*_ can be estimated in a same way, since we can treat it like an internal node, whose set only contains a single cell type *k*:

12 $${\hat{\pi}}_k=\frac{\sum_{g=1}^GI\left( pva{l}_{gk}< threshold\right)}{G}$$

13 $${\hat{p}}_k=\frac{{\hat{\pi}}_k}{{\hat{\pi}}_{\Phi_{l^{\prime },{n}^{\prime }}}}$$

#### Computation of data likelihood

For *K* cell types, ***Z***_*g*_ have 2^*K *^possible combinations. So, totally there are 2^*K *^different linear models to fit. Under each combination of ***Z***_*g*_, *μ*_*gk*_, *β*_*gk*_ and *δ*_*gk*_ (for *k* = 1, …, *K*) are estimated by least square estimators of corresponding linear model (Eq. 1). By assuming the observed bulk signal follows a normal distribution, posterior probability of *Z*_*gk*_ (Eq. 8) can be computed by plugging in the least square estimates. In this work, computation of data likelihood is based on normal distribution assumption, which is often used for microarray data. Specifically, for DNA methylation data, we used beta value for analysis. Even though the beta values for all CpG sites follow a bimodal distribution at around 0 and 1, they can be well approximated by normal distributions for one CpG site cross samples [4, 56]. The same framework could be extended to count data by assuming a negative binomial distribution, which would be our future research direction.

#### Differential signal detection

A feature would be reported showing differential signal in certain cell type if its corresponding posterior probability of DE/DM (Eq. 8) is greater than a user-defined threshold. Higher posterior probability of DE/DM suggests more convincible cell type-specific DE/DM. Besides, the estimated posterior probability of non-DE/non-DM can be viewed as estimated local FDR. The global FDR for a list of features can be derived by averaging their estimated local FDRs.

### Cell type correlation calculation from real data

We obtained two datasets from the GEO database. The first data set (GEO accession number GSE166844 [17]) measures DNA methylation profile on Infinium MethylationEPIC microarray for several purified blood cell types, including CD4 T cells, CD8 T cells, B cells, monocytes, and granulocytes, from 30 individuals (18 females vs. 12 males). The second dataset (GSE60424 [18]) provides gene expression from RNA-seq for six immune cell types (CD4 T cells, CD8 T cells, B cells, NK cells, monocytes, and neutrophils) of sclerosis patients before and 24 hours after the first treatment with IFN-beta. In the DNA methylation data (GSE166844), sites with detection *p*-value greater than or equal to 0.01 in any sample were removed from the processed data set provided on GEO website. We used *minfi* [25–31, 57] to call DM for male vs. female comparison. CpG sites with *q*-value less than 0.05 are deemed differentially methylated sites. For the gene expression data, we used *edgeR* [58–60] to call DE for before vs after first IFN-beta treatment. DE genes are defined as genes with false discovery rate (FDR) less than 0.05.

For both data sets, Pearson correlation coefficient depicting cell type correlation in DE/DM state was calculated based on negative log-transformed (base 10) *p*-values of two cell types and a *t*-test was applied to test whether the correlation estimate is statistically significant different from zero. Odds ratio of DE/DM in two cell types was calculated based on DMC defined above. Each count of the 2 × 2 contingency table was added one to avoid infinite OR value. Fisher’s exact test was used to test whether the estimated odds ratio is statistically significantly different from one.

### Simulation

#### Data simulation

We first estimated cell type-specific mean *μ*_*gk*_ and variance ${\sigma}_{gk}^2$ for gene *g* = 1, …, *G* (*G* = 12,402) in cell type *k* = 1, …, *K* (*K* = 6) (neutrophils, monocytes, CD8 cells, CD4 cells, B cells, and NK cells) from *log* expression values of microarray gene expression data GSE22886 [20]. We defined 10% DE genes between case and control groups in each cell type. Each DE gene has equal probability to be up- or downregulated. To maintain the cell type hierarchy, the DE states of genes were generated based on a pre-defined tree structure (Fig. 1a). The prior probability of each node on the tree is *π*_{1, 2, 3, 4, 5, 6}_ = 0.4, *p*_{1, 2}_ = 0.3125, *p*_1_ = *p*_2_ = 0.8, *p*_{3, 4, 5, 6}_ = 0.5, *p*_{3, 4, 5}_ = 0.8, *p*_6_ = 0.5, *p*_{3, 4}_ = 0.78125, *p*_5_ = 0.625, *p*_3_ = *p*_4_ = 0.8. For root node, among *G* = 12,402 genes, we used Bernoulli distribution with *π*_{1, 2, 3, 4, 5, 6}_ = 0.4 to generate DE state for each feature. Then for one of its child nodes containing cell types 1 and 2, among features with generated potential DE state 1, we used Bernoulli distribution with *p*_{1, 2}_ = 0.3125 to generate DE state. In this way, we can derive DE state of each cell type (each leaf node) and make sure they share different correlation strengths between cell types. For any non-DE gene *g* in case and control groups, its expression in cell type *k* of sample *i,* denoted by *X*_*gik*_, follows a log-normal distribution

$${\log}{X}_{gik}\sim N\left({\mu}_{gk},{\sigma}_{gk}^2\right)$$

For any DE gene *g* in cell type *k* of sample *i* in the case group, the pure expression follows a log-normal distribution

$$\log {X}_{gik}\sim N\left({\mu}_{gk}+ lf{c}_{gk},{\sigma}_{gk}^2\right)$$

where *lfc* is the log2 fold change. For upregulated genes, the log2 fold change (*lfc*_*gk*_) is randomly drawn from normal distribution *N*(1,0.2^2^), while for downregulated genes, it is from *N*(−1,0.2^2^),.

In the simulations setting with six cell types, the mixture proportion of each sample *i*, ***θ***_*i*_, was generated from a Dirichlet distribution with parameters estimated from the real cell type proportion of six cell types (neutrophils, monocytes, CD8 cell, CD4 cell, B cell, and NK cell) [61]: 27*.*94, 4*.*64, 2*.*47, 4*.*87, 2*.*30, 2*.*21. In the simulation setting for evaluating the impact of different cell type hierarchy, the four cell types selected were neutrophils, monocytes, CD8 cell, and CD4 cells, and the corresponding Dirichlet parameter was 27*.*94, 4*.*64, 2*.*47, and 9*.*38. We assumed there is no cell type proportion difference between the case and control groups.

Finally, we simulated *s* cases and *s* controls (*s* = 50*,* 100*,* 200 for different simulations). The simulated measurement for gene *g* of sample *i*, *Y*_*gi*_, is a linear combination of simulated cell type-specific expression ***X***_***gi***_ = (*X*_*gi*1_, …, *X*_*giK*_) weighted by the mixture proportion ***θ***_***i***_, and added by a random noise *ϵ*_*gi*_:

$${Y}_{gi}=\boldsymbol{X}_{\boldsymbol{gi}}\boldsymbol{\theta}_{\boldsymbol{i}}^{\boldsymbol{T}}+{\epsilon}_{gi}$$

We assumed the random noises are mutually independent for each gene and each sample. To reflect the mean-variance dependence of gene expression, we assumed the variance of the random noise is positively correlated with gene expression:

$${\epsilon}_{gi}\sim N\left(0,{\eta}_g^2\right)$$

where $${\eta}_g=0.1\times \max \left(\ {\sum}_{i: control}\frac{\boldsymbol{X}_{\boldsymbol{gi}}\boldsymbol{\theta}_{\boldsymbol{i}}^{\boldsymbol{T}}}{s},{\sum}_{i: case}\frac{\boldsymbol{X}_{\boldsymbol{gi}}\boldsymbol{\theta}_{\boldsymbol{i}}^{\boldsymbol{T}}}{s}\right)$$.

#### Cell type proportion estimation

In the second simulation results section, we evaluated robustness of CeDAR to estimated proportions. We estimated the cell type proportion for each sample from the mixture profiles by using RB method *lsfit* from the R package *CellMix* [62]. The estimated cell type-specific mean from GSE22886, which was used for generating pure cell type expression, was used as a reference profile. Reported marker genes for the six blood cell types [53] were used for deconvolution. Proportions of samples in cases and controls were estimated separately.

#### Evaluation of CeDAR method

After deriving the simulated bulk data and corresponding proportion, we compared CeDAR method with TOAST and TCA. We used ROC to evaluate the accuracy of proposed method and calculated observed FDR at a given cutoff to evaluate type I error control. For the detail of evaluation method used in simulation, please see Additional file 1: Section S1.

### Real data analysis

We downloaded three DNA methylation datasets (GSE41826, GSE166844, GSE42861) from GEO database. The methylation level is measured with beta value. R package *minfi* [25–31] was used to pre-process raw data and call gold standard csDMCs. For datasets with pure cell type samples, we defined gold standard of cell type-specific DM state by setting sites with FDR smaller than 0.01 as true DM, with FDR greater than 0.8 as non-DM. For detecting cell type-specific effects in bulk data, we first used *EpiDISH* [4, 53, 63–66] to estimate cell type compositions. The DNA methylation reference is mean profile of each cell type for GSE41826 and GSE166844; for GSE42861, which does not have pure cell type samples, DNAm reference consists of 333 immune cell type-specific DMCs [63, 64]. More details are provided in Additional file 1: Section S2-S4.

## Supplementary Information

**Additional file 1:** Supplementary Section S1.(Evaluation of CeDAR method); S2 (Cell-type-specific differential methylation in brain); S3 (Cell-type-specific differential methylation in whole blood); S4 (Cell-type-specific differential methylation in RA EWAS study); S5 (Additional real data analysis showing DE/DM state correlations among cell types); S6 (Additional simulation analysis evaluating impact of data noise on observed FDR for CeDAR method); S7 (Additional simulation analysis evaluating impact of mis-specified tree structures as input of CeDAR-M); S8 (Additional real data analyses); Figure S1 – S12; Table S1 – S13.

**Additional file 2:** Review history.

## Data Availability

The Gene expression and DNA methylation data sets used and analyzed in this study are available in the Gene Expression Omnibus (GEO) repository under the following accession IDs: GSE60424 [18, 67], GSE166844 [17, 68], GSE22886 [20, 68, 69], GSE41826 [24, 70], GSE42861 [32, 71], GSE149050 [72, 73], GSE59250 [74, 75], GSE131525 [76, 77], GSE40279 [52, 78], GSE74486 [50, 79], GSE118144 [51, 80]. The proposed method is implemented in Bioconductor package TOAST, which is freely available at https://www.bioconductor.org/packages/release/bioc/html/TOAST.html [81]. The scripts generating reported results is accessible on GitHub: https://github.com/luxiao10/CeDAR_reproduction [82]. Both repositories are licensed under the open-source GPL-2.0. The version of software package used to produce the results reported in the paper was also deposited at Zenodo: 10.5281/zenodo.7272410 [83]. A summary of the details of all simulated and real datasets are provided in Additional file 1 as Table S10, Table S11, and Table S12. A summary of the tools used for the evaluation of CeDAR performance in simulated and real data analysis is in Additional file 1 Table S13.
