# Supplementary material for: CeDAR: incorporating cell type hierarchy improves cell type-specific differential analyses in bulk omics data
Source: Genome Biol. 2023 Feb 28;24:37. doi: 10.1186/s13059-023-02857-5 (PMC9972684; doi:10.1186/s13059-023-02857-5)
Supplement: Supplementary file 1 — Additional file 1: Supplementary Section S1.(Evaluation of CeDAR method); S2 (Cell-type-specific differential methylation in brain); S3 (Cell-type-specific differential methylation in whole blood); S4 (Cell-type-specific differential methylation in RA EWAS study); S5 (Additional real data analysis showing DE/DM state correlations among cell types); S6 (Additional simulation analysis evaluating impact of data noise on observed FDR for CeDAR method); S7 (Additional simulation analysis evaluating impact of mis-specified tree structures as input of CeDAR-M); S8 (Additional real data analyses); Figure S1 – S12; Table S1 – S13. [file 13059_2023_2857_MOESM1_ESM.docx]

CeDAR: incorporating cell type hierarchy improves cell type specific

differential analyses in bulk omics data

Additional file 1

Luxiao Chen^1^, Ziyi Li^2^ & Hao Wu^1^*

1. Department of Biostatistics and Bioinformatics, Emory University, Atlanta, GA 30322, USA
2. Department of Biostatistics, The University of Texas MD Anderson Cancer Center, Houston, TX77030, USA

*Correspondence: [hao.wu@emory.edu](mailto:hao.wu@emory.edu)

## S1. Evaluation of CeDAR method

Given simulated mixture gene expression and known cell type proportion in each sample, TOAST was run first to provide cell type specific DE inference result. Then tree structure depicting cell types correlation was estimated by following estimation procedure Methods section 4.2.1 for CeDAR-M based on the TOAST results. Genes with FDR smaller than 0.01 in any cell type were selected for tree structure estimation. For CeDAR-S method, an arbitrarily defined single layer tree was used directly. After deriving estimated tree structure, prior probability on each node of the tree was estimated by following proposed estimation procedures with the threshold set as 0.01. In TCA, variances in the model are learned by maximum likelihood estimation. In csSAM, all results are based on 200 permutations. To evaluate the accuracy of each method (CeDAR-S, CeDAR-M, TOAST, TCA, csSAM and CellDMC), the threshold-averaged ROC curve (1), area under receiver operating characteristic curve (AUC-ROC), and area under precision-recall curves (AUC-PR) were calculated based on 50 simulations by using R package *ROCR* (2). To evaluate the FDR control of these methods, observed FDR was calculated at cut off (TOAST, TCA, csSAM, CellDMC: estimated FDR; CeDAR-S, CeDAR-M: posterior probability of non-DEG) 0.05. In addition, another metric - Matthews correlation coefficient (MCC), which is used to measure quality of binary classification, was calculated at cut off (TOAST, TCA, csSAM, CellDMC: estimated FDR; CeDAR-S, CeDAR-M: posterior probability of non-DEG) 0.05. Both reported observed FDR and MCC results are average of 50 simulations.

## S2. Cell-type-specific differential methylation in brain

We downloaded the processed Illumina 450k data, which contains both bulk brain tissue samples and pure sorted neuronal and glia samples for a number of individuals with sex information, from GEO with accession number GSE41826 (3). We used function *dmpFinder* in R package *minfi (4-9)* to call DMCs by performing two group comparison between the fourteen healthy males and fifteen healthy females in each cell type based on pure cell type profiles. True DM sites were defined when corresponding FDR is smaller than 0.01; non-DM sites were defined when corresponding FDR is greater than 0.8. The DNA methylation reference used for estimating mixture proportions of bulk samples is the mean profile of each cell type. The top 1,000 sites with largest variance among bulk samples were used to estimate mixture proportion with *EpiDISH* (10-15) in RPC mode. Threshold used in CeDAR-S to estimate prior probability on each node is pval = 10^−5^. Result of csSAM is based on 200 permutations.

## S3. Cell-type-specific differential methylation in whole blood

We downloaded the processed Illumina MethylationEPIC data with the whole blood profiles, as well as the cell-sorted CD4 T cells, CD8 T cells, B cells, monocytes, granulocytes profiles for 30 individuals (GSE166844 (16)). In the QC step, any site with detection p-value greater than 0.01 in any sample was removed. There are 757,133 sites kept and 45,083 sites removed. We used function *dmpFinder* in R package *minfi* to call DMCs by performing two group comparison between twelve healthy males and eighteen healthy females in each cell type based on pure cell type profiles. True DM sites were defined when corresponding FDR is smaller than 0.01; non-DM sites were defined when corresponding FDR is greater than 0.8. The DNA methylation reference used for estimating mixture proportions of bulk samples is the mean profile of each cell type. Cell type specific markers were selected with two sample t-test by setting target cell type samples as one group, and all other samples corresponding to remaining cell types as the other group. Sites with FDR smaller than 0.05 and beta value with a 0.2 difference greater than any other cell types were selected as markers. We selected 10 markers per cell type with the largest variance among bulk samples to estimate mixture proportion with *EpiDISH* in RPC mode. Then tree structure depicting cell types correlation was estimated by following estimation procedure Methods section 4.2.1 for CeDAR-M based on the TOAST results. Genes with FDR smaller than 0.01 in any cell type were selected for tree structure estimation. Threshold used in CeDAR-S and CeDAR-M to estimate prior probability is pval = 10^−5^. Result of csSAM is based on 200 permutations.

## S4. Cell-type-specific differential methylation in RA EWAS study

We downloaded raw Illumina 450K data with the peripheral blood lymphocytes profile for 332 normal individuals and 354 rheumatoid arthritis (RA) patients (GSE42861 (17, 18)). Any probe with detection p-value greater than the threshold ${10}^{-16}$ was treated as missing value. Samples with call rate $<95\%$ and probes with call rate $<90\%$ were excluded. Probes located on chromosome X and chromosome Y were removed. We also dropped probes containing a SNP at the CpG interrogation and/or at the single nucleotide extension. Two samples without smoking status information were removed. Normalization was completed by “*Funnorm*” method(6) in *minfi*. Missing values were imputed by function “impute.knn” in R package “impute” (19). Finally, beta value was calculated for cell type specific DM analysis. We estimated cell type fractions of six major immune cell types (B cells, CD4, CD8, NK, and monocytes) by using *EpiDISH* in RCP mode with a DNAm reference consisting of 333 immune cell type-specific DMCs (12). In the cell type specific DM analysis, both disease state (RA vs. normal) and age are assumed to have cell type specific effects, while smoking status and gender were treated as global confounders (have same effect on all cell types). In TCA, variances in the model are learned by maximum likelihood estimation. Same as simulation settings, for TOAST, TCA, csSAM and CellDMC, probes with FDR < 0.05 were reported as DMC; for CeDAR-S and CeDAR-M, probes with posterior probability of DM > 0.95 were reported as DMC. Enrichment analysis were performed with *gometh* function in package *missMethyl* (20) for *KEGG* (21-23) pathways.

## S5. Additional real data analysis showing DE/DM state correlations among cell types

We obtained three additional datasets from GEO database, which measure gene expression/DNA methylation profiles of different cell types from samples of different groups. The first data set (GEO accession number GSE149050 (24)) contains gene expression profile (raw counts) from RNA-seq for six major circulating immune cell types (T cells, B cells, polymorphonuclear neutrophils, conventional dendritic cells, plasmacytoid dendritic cells, classical monocytes) from blood of healthy subjects and Systemic Lupus Erythematosus (SLE) patients with high expressed type I interferon – related genes. The second dataset (GSE59250 (25)) contains DNA methylation profiles (normalized beta value) measured by Illumina HumanMethylation450 for cell types (CD4 T cells, CD8 T cells, and monocytes) of SLE patients and controls. The third dataset (GSE131525 (26)) contains gene expression profile (raw counts) from RNA-seq for cell types (CD4 T cells, CD8 T cells, B cells and monocytes) of SLE patients and healthy subjects. For DNA methylation data (GSE59250), we used function *dmpFinder* in R package *minfi* to call DM for SLE vs. control comparison. CpG site with q-value less than 0.05 are deemed differentially methylated sites. For the gene expression data (GSE149050, GSE131525), we used *DEseq2 (27)* to call DE for SLE vs. control comparison. DE genes are defined as genes with false discovery rate (FDR) less than 0.05. Then, we evaluated the pairwise correlation among cell types in terms of their DE/DM status, using both Pearson correlation coefficient (PCC) of log transformed p-values from the DE/DM tests for all features, and the odds ratio (OR) of being DE/DM from the cell types.

The pairwise scatterplots for the comparisons are shown in Figure S1. In data GSE149050 (Figure S1a), the p-values from all cell types are statistically significant positive that the smallest PCC is 0.36 between B cells and polymorphonuclear neutrophils (PMN) and the largest PCC is 0.63 between classical monocytes (cMo) and conventional dendritic cells (cDC)/PMN. Besides, the ORs for being DE between any two cell types are also statistically greater than 1 that smallest OR is 3.0 between B cells and PMN and the largest OR is 24 between classical monocytes and plasmacytoid dendritic cells. In data GSE59250 (Figure S1b), even though the PCCs are smaller than those in GSE149050 that the largest value is 0.25 between CD4 and B cells, the ORs are all statistically significantly greater than 1 that smallest value is 26 between CD4 and monocytes, and largest value is 180 between B cells and monocytes. The results of the two data sets indicate existence of DE/DM state correlation among cell types. In addition, in data GSE131525 (Fig S1c), we can observe that between CD4 and CD8 both PCC (0.65) and OR (37) are greatly larger than other pairs of cell types (remaining largest PCC is 0.35, largest OR is 7.1), which implies a cell type hierarchy of DE/DM state correlation. Overall, these results demonstrate that there are strong correlations among cell types in terms of their DE/DM status.

## S6. Additional simulation analysis evaluating impact of data noise on observed FDR for CeDAR method

To illustrate the effect of data noise on observed FDR from CeDAR, we performed simulation with different data noise levels (extremely low: 0.01, low: 0.1, normal: 1, high: 2). In the settings, normal level (noise level 1) is the setting we used in our reported simulations. We modify the noise level by multiplying 0.01, 0.1 or 2 to the standard deviation of both cell type specific gene expression and bulk gene expression.

We first performed the simulation on two cell types with proportion ratio 9:1. As can be seen from Table S5, when noise level is low (0.01, 0.1), the FDR of cell type 2 with true prior is still smaller with estimated prior (0.024 vs. 0.039, 0.025 vs. 0.037). But when noise level is larger (1, 2), we can observe larger FDR in cell type 2 with true prior (0.083 vs. 0.047, 0.225 vs. 0.126).

We then performed the simulation on six cell types with true/estimated prior probability and tree structure on the four different noise levels (extremely low: 0.01, low: 0.1, normal: 1, high: 2). Same conclusion can be derived from Table S6 that when data noise is small (noise level 0.01, 0.1), CeDAR with true prior probability has lower FDR than CeDAR with estimated prior probability (e.g., in cell type 2, 0.066 vs. 0.089, 0.069 vs. 0.080). When data noise is larger (noise level 1, 2), CeDAR with true prior probability has higher FDR than CeDAR with estimated prior probability (e.g., in cell type 2, 0.165 vs. 0.073, 0.345 vs. 0.206).

Overall, the FDR difference between CeDAR with true prior probability and estimated probability is related with data noise. When data noise is large, CeDAR with estimated prior prob has smaller FDR and when data noise is small, CeDAR with true prior prob has smaller FDR.

## S7. Additional simulation analysis evaluating impact of mis-specified tree structures as input of CeDAR-M

To evaluate impact of mis-specified tree structure as input for CeDAR-M, we designed additional simulation with either correct or mis-specified tree structure as input for CeDAR-M. Correct tree structure means applying the tree structure generating DE state in simulation data as input of CeDAR-M, while mis-specified tree structure means applying tree structures with cell types arbitrarily switched under nodes. The simulation is performed with six cell types under different sample sizes per group (50, 100, 200). The evaluation process is similar as process described in section S1, except that all the tree structures are pre-specified without estimation.

We performed simulation with six cell types (proportions of cell type 1-6: 0.63, 0.10, 0.11, 0.06, 0.06 and 0.05). The “correct” tree structure and “mis-specified” tree structures are shown in the top row of Figure S6 and S7 (Correct: “tree 1”; Mis-specified: “tree 2”, “tree 3”, “tree 4” and “tree 5”). In “correct” tree structure, cell type 1 and 2 are set under same node, while cell type 4, 5, 6 are set under same node with cell type 3 but with different DE state correlation level. In “mis-specified” tree structures, we switch cell type 2 with cell type 3/4 (“tree 2”/ “tree 5”) to check impact of a cell type mis-clustered with small proportion cell types Besides, we also switch cell type 4 with cell type 5/6, which decreases DE state correlation between cell type 4 and cell type 3. Such misspecification is common during estimation process because small proportion providing less information for accurate clustering.

The simulation result (Figure S6, S7, and Table S7) shows that using “mis-specified” tree structures as input of CeDAR-M has small impact on csDE inference compared to using “correct” tree structure. For cell types with large proportion (e.g., cell type 1 with mean proportion 0.63), we can barely observe difference of ROC curves and boxplot of observed FDR between correct and mis-specified tree structures. When cell type 1 is clustered with cell type 3 or cell type 4 (“tree 2”/ “tree5”), compared to “correct” tree structure the decrease of AUC-ROC, AUC-PR, MCC and increase of observed FDR are extremely small. For example, with sample size 100 per group, the AUC-ROC for cell type 1 with “tree 1” vs. “tree 2” is 0.989 vs. 0.987, and the observed FDR is 0.068 vs. 0.073. For small proportion cell types that are mis-clustered with other weak correlated cell types that have small proportions (e.g., cell type 2 in “tree 2” and “tree 5”, cell type 4 in “tree 2”), we can observe decrease of AUC-ROC and inflation of observed FDR compared to the result with “tree 1”. For example, with sample size 100 per group, the AUC-ROC for cell type 2 with “tree 1” vs. “tree 2” is 0.919 vs. 0.871, and the observed FDR is 0.070 vs. 0.102. Such change is because cell types with relatively small proportions (cell type 4, 5, 6) cannot provide accurate information as cell type 1. Besides, for cell type 2, “tree 3” and “tree 4” have similar performance in AUC-ROC and observed FDR as “tree 1”, which indicates that when cell type 2 is correctly clustered with large proportion cell type 1, the mis-specified tree structure in other sibling nodes have little impact on it. For small proportion cell types that are mis-clustered with other cell types under same non-root node (e.g., cell type 4, 6 in “tree 3” and cell type 4, 5 in “tree 4”), we can observe that the change of AUC-ROC and observed FDR is small compared to “tree 1”. For example, with sample size 100 per group, the AUC-ROC for cell type 4 with “tree 1” vs. “tree 4” is 0.850. vs. 0.847, and for cell type 5 is 0.829 vs. 0.830; the observed FDR for cell type 4 with “tree 1” vs. “tree 4” is 0.097 vs. 0.092, and for cell type 5 is 0.129 vs. 0.138. In addition, with increasing sample size, CeDAR-M performance with “mis-specified” tree can improve. For example, from sample size 50 to 200, the AUC-ROC of cell type 2 in “tree 2” increases from 0.852 to 0.894 and the observed FDR decreases from 0.175 to 0.075. Overall, using “mis-specified” tree structure as input has little impact on cell types with large proportion, cell types that are correctly clustered in sibling nodes, or cell types that are mis-clustered with other cell types in same non-root node. The main impact of “mis-specified” tree structure (decrease of AUC-ROC, inflation of observed FDR) is observed for small proportion cell types that are clustered with other weak correlated (under different non-root node) cell types with small proportion. Meanwhile, with increasing sample size, the performance of CeDAR-M with “mis-specified” tree structure can be improved.

## S8. Additional real data analyses

We applied CeDAR for three more real data analyses and compared it with other methods (TOAST, TCA, csSAM and CellDMC). The first two analyses were performed separately on Down syndrome (DS) methylation data (GSE74486 (28)) and Systemic Lupus Erythematosus (SLE) methylation data (GSE118144 (29)), which contain both bulk samples and pure cell type samples. We identified cell type specific differential methylation sites from pure cell type samples and use them as gold standard to benchmark the result of csDM analysis on bulk samples. In the third analysis, we performed csDM analysis on two DNA methylation data (GSE42861 and GSE40279 (30)) and examined whether methods in comparison can identify seven reported smoking associated cell type specific probes.

**Cell-type-specific differential methylation in Down syndrome study**

The DS methylation data (GSE74886) contains both bulk samples of frontal cortex grey matter (14 DS vs. 8 normal) and pure cell type samples of glia and neuron cells derived by FACS from DS subjects and healthy control subjects. We first performed two-group comparison (DS vs. normal) separately for glia and neuron samples to identify csDMCs serving for gold standard by using *dmpFinder* function in *minfi* package with default settings. We defined sites with FDR < 0.01 as true DM; FDR > 0.8 as non-DM in the two cell types. Among all 390,089 sites, there are 8,099 and 12,438 true DM sites identified in glia and neuron respectively. The two cell types share 1,284 common true DM sites. We estimated the mixture proportions for each bulk sample by using *EpiDISH* with RPC-mode, in which the mean profile of each cell type is used as reference and top 1,000 sites with largest variance among bulk samples were used for deconvolution. The estimated mixture proportions and the whole-tissue DNA methylation data were used as inputs for TOAST, TCA, csSAM, CellDMC and CeDAR-S. Result of csSAM is generated based on 200 permutations. Threshold used in CeDAR-S to estimate prior probability on each node is pval = 10^−5^. Accuracy was measured by true discovery rate (TDR) in top ranked sites. The TDR curves in Figure S10 show that CeDAR-S has significantly higher accuracy among the top CpG sites than all other methods in both glia and neuron that the differences of TDR between CeDAR-S and TOAST among top ranked 5,000 sites in both cell types are more than 20%.

**Cell-type-specific differential methylation in Systemic Lupus Erythematosus study**

The SLE methylation data (GSE118114) contains both bulk samples of whole blood (16 SLE vs. 13 normal) and pure cell type samples of neutrophils, CD8, CD4, and B cells from SLE patients and healthy control subjects. We performed two-group comparison (SLE vs. normal) separately for neutrophils, CD8, CD4 and B cells to identify csDMCs serving for gold standard by using *dmpFinder* function in *minfi* package with default settings. We defined sites with FDR < 0.01 as true DM; FDR > 0.8 as non-DM in the four cell types. Among all the 662,741 sites, there are 5,425 (neutrophils), 6,886 (CD4), 59 (CD8), 25 (B cells) true DM sites identified. We estimated the mixture proportions for each bulk sample by using *EpiDISH* with RPC-mode, in which the reference is a DNAm reference consisting of 333 immune cell type-specific DMCs (12) and sites in both reference data and bulk samples were kept for deconvolution. The estimated mixture proportions and the whole-tissue DNA methylation data were used as inputs for TOAST, TCA, csSAM, CellDMC, CeDAR-S and CeDAR-M. Result of csSAM is generated based on 200 permutations. Sites with p-value smaller than 0.01 in any cell type were selected for tree structure estimation. Threshold used in CeDAR-S and CeDAR-M to estimate prior probability on each node is pval = 10^−5^. Accuracy was measured by true discovery rate (TDR) in top ranked sites. The TDR curves in Figure S11 show that both CeDAR-S and CeDAR-M have higher accuracies than all other methods. For example, in cell type neutrophils, which has largest mean proportion (0.67), we can see that CeDAR-S and CeDAR-M have higher TDR curve than other methods. Meanwhile, in low abundant cell types, like CD4 (mean proportion: 0.064), the performances of all methods are not good, but only CeDAR-S and CeDAR-M can identify some true DM sites among top ranked 5,000 sites. This can also be observed in cell type CD8 and B cells, which only have 59 and 25 true DM sites respectively.

**Cell-type-specific differential methylation analysis for smoking associated DNA methylation sites**

Su et al. (31) reported five smoking associated Myeloid-specific DM sites (cg05575921, cg21566642, cg09935388, cg06126421, and cg03636183) and two smoking associated Lymphoid-specific DM sites (cg19859270 and cg09099830). We performed csDM analysis on two DNA methylation data (Liu’s data: GSE42861 and Hannum’s data: GSE40279) to check whether CeDAR and other methods can identify these csDMCs. In the analysis we compare CeDAR-S with TOAST, TCA, csSAM and CellDMC.

For Liu’s data, after preprocessing described in section S5, proportions of seven blood cell types (B cells, CD4, CD8, NK, monocytes, neutrophils, and eosinophils) were first estimated by EpiDISH with RPC-mode, which using DNAm reference consisting of 333 immune cell type specific DMCs as reference. Then proportion of lymphoid is the summation of estimated proportions of B cells, CD4, CD8, and NK cells. Similarly, proportion of myeloid is the summation of estimated proportions of monocytes, neutrophils, and eosinophils. We defined smoking status as binary variable (never vs. smoke) that never-smokers are in “never” group, while ex-smokers, occasional-smokers and current-smokers are in “smoke” group. In the cell type specific DM (csDM) analysis, disease state, age, and smoking status are assumed to have cell type specific effect, and gender is treated as global confounder (have same effect on all cell types). For Hannum’s data, preprocessed data was derived online from figshare (<https://figshare.com/articles/online_resource/CompCellDMCtoTCA/12922322/1>) and the proportion estimation process is same as Liu’s data. Similarly, we defined smoking status as binary variable (never vs. smoke) that never-smokers are in “never” group, while ex-smokers and current-smokers are in “smoke” group. In the csDM analysis, age and smoking status are assumed to have cell type specific effect, and plate is treated as global confounder. In both analyses, threshold used in CeDAR-S to estimate prior probability on each node is pval = 10^−5^. Same as simulation settings, for TOAST, TCA, csSAM and CellDMC, probes with FDR < 0.05 were reported as DMC; for CeDAR-S, probes with posterior probability of DM > 0.95 were reported as DMC. As can be seen from Figure S12, CeDAR-S can identify more smoking associated DNA methylation sites reported by Su et. al. than other four methods in both Liu’s data and Hannum’s data. In Liu’s data, while TOAST, TCA, csSAM and CellDMC can identify four myeloid-specific sites (cg05575921, cg21566642, cg06126421, and cg03636183) but zero lymphoid-specific sites, CeDAR-S can identify all myeloid-specific sites and one more lymphoid-specific site (cg19859270). Meanwhile, CeDAR-S identified a myeloid-specific site (cg03636183) in lymphoid cells. In Hannum’s data, while TOAST, TCA and CellDMC can only identify one myeloid-specific site (cg05575921) and csSAM cannot identify any site, CeDAR-S can identify four out of five myeloid-specific sites (cg05575921, cg09935388, cg06126421, and cg03636183) and one lymphoid-specific site (cg19859270) with one myeloid-specific (cg21566642) site identified in lymphoid cells.

Overall, all the three analyses demonstrate that incorporating DM state correlation among cell types can improve accuracy and power in csDM analysis.

**References**

1. Fawcett T. An introduction to ROC analysis. Pattern Recog Lett. 2006;27(8):861-74.

2. Sing T, Sander O, Beerenwinkel N, Lengauer T. ROCR: visualizing classifier performance in R. Bioinformatics. 2005;21(20):3940-1.

3. Guintivano J, Aryee MJ, Kaminsky ZA. A cell epigenotype specific model for the correction of brain cellular heterogeneity bias and its application to age, brain region and major depression. Epigenetics. 2013;8(3):290-302.

4. Aryee MJ, Jaffe AE, Corrada-Bravo H, Ladd-Acosta C, Feinberg AP, Hansen KD, et al. Minfi: a flexible and comprehensive Bioconductor package for the analysis of Infinium DNA methylation microarrays. Bioinformatics. 2014;30(10):1363-9.

5. Maksimovic J, Gordon L, Oshlack A. SWAN: Subset-quantile within array normalization for illumina infinium HumanMethylation450 BeadChips. Genome biology. 2012;13(6):1-12.

6. Fortin J-P, Labbe A, Lemire M, Zanke BW, Hudson TJ, Fertig EJ, et al. Functional normalization of 450k methylation array data improves replication in large cancer studies. Genome biology. 2014;15(11):1-17.

7. Triche Jr TJ, Weisenberger DJ, Van Den Berg D, Laird PW, Siegmund KD. Low-level processing of Illumina Infinium DNA methylation beadarrays. Nucleic Acids Res. 2013;41(7):e90-e.

8. Andrews SV, Ladd-Acosta C, Feinberg AP, Hansen KD, Fallin MD. “Gap hunting” to characterize clustered probe signals in Illumina methylation array data. Epigenetics & chromatin. 2016;9(1):1-21.

9. Fortin J-P, Triche T, Hansen K. Preprocessing, normalization and integration of the Illumina HumanMethylationEPIC array. bioRxiv.65490(2016).

10. Zheng SC, Breeze CE, Beck S, Teschendorff AE. Identification of differentially methylated cell types in epigenome-wide association studies. Nat Methods. 2018;15(12):1059-66.

11. Zheng SC, Webster AP, Dong D, Feber A, Graham DG, Sullivan R, et al. A novel cell-type deconvolution algorithm reveals substantial contamination by immune cells in saliva, buccal and cervix. Epigenomics. 2018;10(7):925-40.

12. Teschendorff AE, Breeze CE, Zheng SC, Beck S. A comparison of reference-based algorithms for correcting cell-type heterogeneity in Epigenome-Wide Association Studies. BMC Bioinformatics. 2017;18(1):1-14.

13. Newman AM, Liu CL, Green MR, Gentles AJ, Feng W, Xu Y, et al. Robust enumeration of cell subsets from tissue expression profiles. Nat Methods. 2015;12(5):453-7.

14. Teschendorff AE, Zheng SC. Cell-type deconvolution in epigenome-wide association studies: a review and recommendations. Epigenomics. 2017;9(5):757-68.

15. Houseman EA, Accomando WP, Koestler DC, Christensen BC, Marsit CJ, Nelson HH, et al. DNA methylation arrays as surrogate measures of cell mixture distribution. BMC Bioinformatics. 2012;13(1):1-16.

16. Hannon E, Mansell G, Walker E, Nabais MF, Burrage J, Kepa A, et al. Assessing the co-variability of DNA methylation across peripheral cells and tissues: Implications for the interpretation of findings in epigenetic epidemiology. PLoS Genet. 2021;17(3):e1009443.

17. Liu Y, Aryee MJ, Padyukov L, Fallin MD, Hesselberg E, Runarsson A, et al. Epigenome-wide association data implicate DNA methylation as an intermediary of genetic risk in rheumatoid arthritis. Nat Biotechnol. 2013;31(2):142-7.

18. Kular L, Liu Y, Ruhrmann S, Zheleznyakova G, Marabita F, Gomez-Cabrero D, et al. DNA methylation as a mediator of HLA-DRB1* 15: 01 and a protective variant in multiple sclerosis. Nature communications. 2018;9(1):1-15.

19. Trevor Hastie RT, Balasubramanian Narasimhan and Gilbert Chu. impute: impute: Imputation for microarray data. 2021.

20. Phipson B, Maksimovic J, Oshlack A. missMethyl: an R package for analyzing data from Illumina’s HumanMethylation450 platform. Bioinformatics. 2016;32(2):286-8.

21. Kanehisa M, Goto S. KEGG: kyoto encyclopedia of genes and genomes. Nucleic Acids Res. 2000;28(1):27-30.

22. Kanehisa M, Furumichi M, Sato Y, Ishiguro-Watanabe M, Tanabe M. KEGG: integrating viruses and cellular organisms. Nucleic Acids Res. 2021;49(D1):D545-D51.

23. Kanehisa M. Toward understanding the origin and evolution of cellular organisms. Protein Sci. 2019;28(11):1947-51.

24. Panwar B, Schmiedel BJ, Liang S, White B, Rodriguez E, Kalunian K, et al. Multi–cell type gene coexpression network analysis reveals coordinated interferon response and cross–cell type correlations in systemic lupus erythematosus. Genome Res. 2021;31(4):659-76.

25. Absher DM, Li X, Waite LL, Gibson A, Roberts K, Edberg J, et al. Genome-wide DNA methylation analysis of systemic lupus erythematosus reveals persistent hypomethylation of interferon genes and compositional changes to CD4+ T-cell populations. PLoS Genet. 2013;9(8):e1003678.

26. Speake C, Skinner SO, Berel D, Whalen E, Dufort MJ, Young WC, et al. A composite immune signature parallels disease progression across T1D subjects. JCI insight. 2019;4(23).

27. Love MI, Huber W, Anders S. Moderated estimation of fold change and dispersion for RNA-seq data with DESeq2. Genome biology. 2014;15(12):1-21.

28. Mendioroz M, Do C, Jiang X, Liu C, Darbary HK, Lang CF, et al. Trans effects of chromosome aneuploidies on DNA methylation patterns in human Down syndrome and mouse models. Genome biology. 2015;16(1):1-26.

29. Yeung KS, Lee TL, Mok MY, Mak CCY, Yang W, Chong PCY, et al. Cell lineage-specific genome-wide DNA methylation analysis of patients with paediatric-onset systemic lupus erythematosus. Epigenetics. 2019;14(4):341-51.

30. Hannum G, Guinney J, Zhao L, Zhang L, Hughes G, Sadda S, et al. Genome-wide methylation profiles reveal quantitative views of human aging rates. Mol Cell. 2013;49(2):359-67.

31. Su D, Wang X, Campbell MR, Porter DK, Pittman GS, Bennett BD, et al. Distinct epigenetic effects of tobacco smoking in whole blood and among leukocyte subtypes. PloS one. 2016;11(12):e0166486.

32. Julià A, Absher D, López-Lasanta M, Palau N, Pluma A, Waite Jones L, et al. Epigenome-wide association study of rheumatoid arthritis identifies differentially methylated loci in B cells. Hum Mol Genet. 2017;26(14):2803-11.


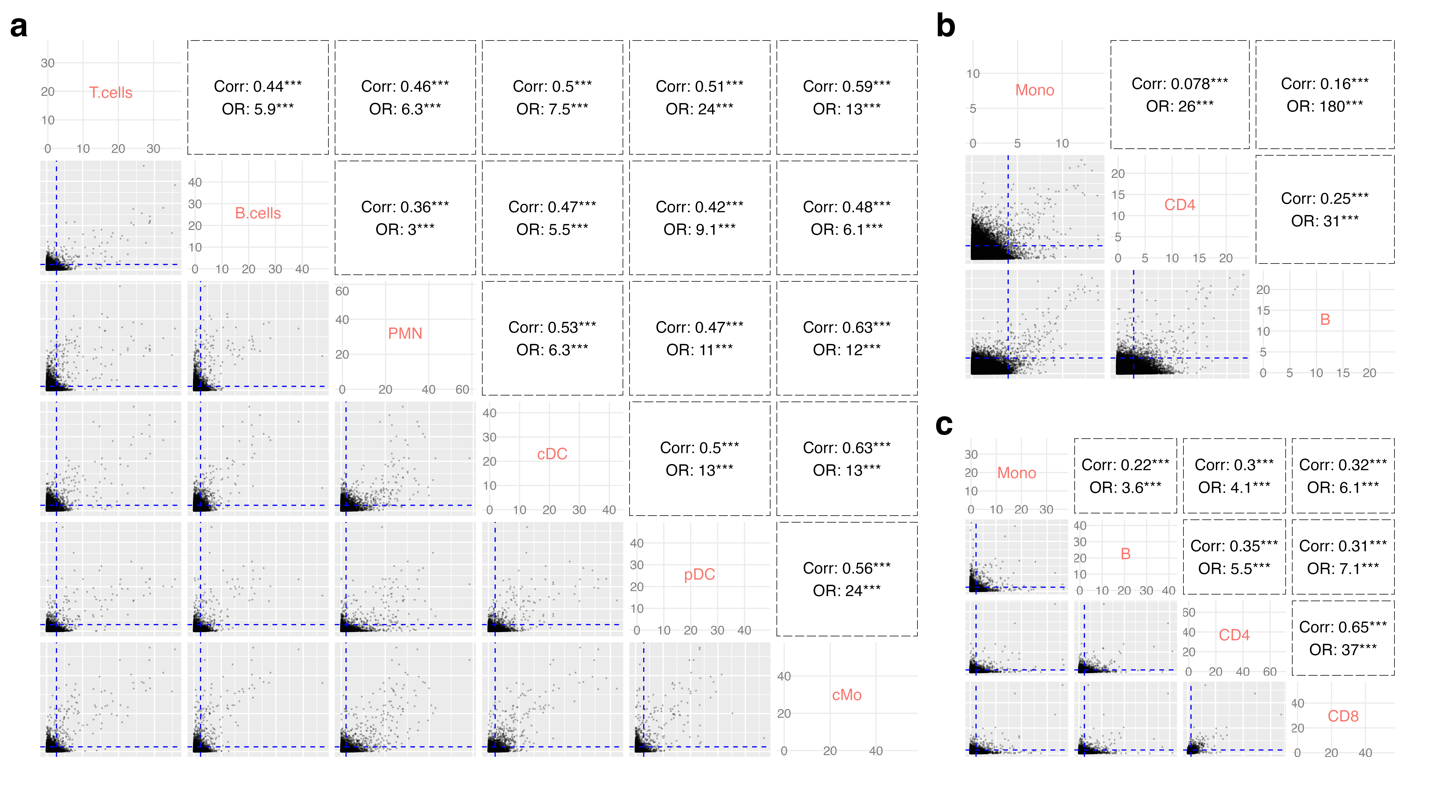


Figure S1: Correlations among cell types from cell type specific differential analysis. (a) cell type specific differential expression analysis on data GSE149050 (healthy controls vs. SLE patients with high expressed type I interferon – related genes) in major circulating immune cell types (T cells, B cells, Polymorphonuclear Neutrophils (PMNs), conventional dendritic cells (cDC), plasmacytoid dendritic cells (pDC), classical monocytes (cMo)); (b) cell type specific differential methylation analysis on data GSE59250 (lupus patients vs. controls) in cell types (CD14+ Monocytes, CD4+ T-cells, and CD19+ B-cells); (c) cell type specific differential expression analysis on data GSE131525 (SLE patients vs. healthy controls) in cell types (Monocytes, CD8+ T-cells, CD4+ T-cells, and B cells). DE/DM tests were applied for each feature in each cell type. X-axis and Y-axis represent -log10 transformed p-value from DE/DM tests in corresponding cell types. Each point represents a gene or CpG site. Dashed blue lines represent the thresholds used to define DEG/DMC in each cell type. Pearson correlation coefficients (PCC) of transformed p-values and odds ratio (OR) of differential state are tested for their significance. *** represents p-value < 0.01.


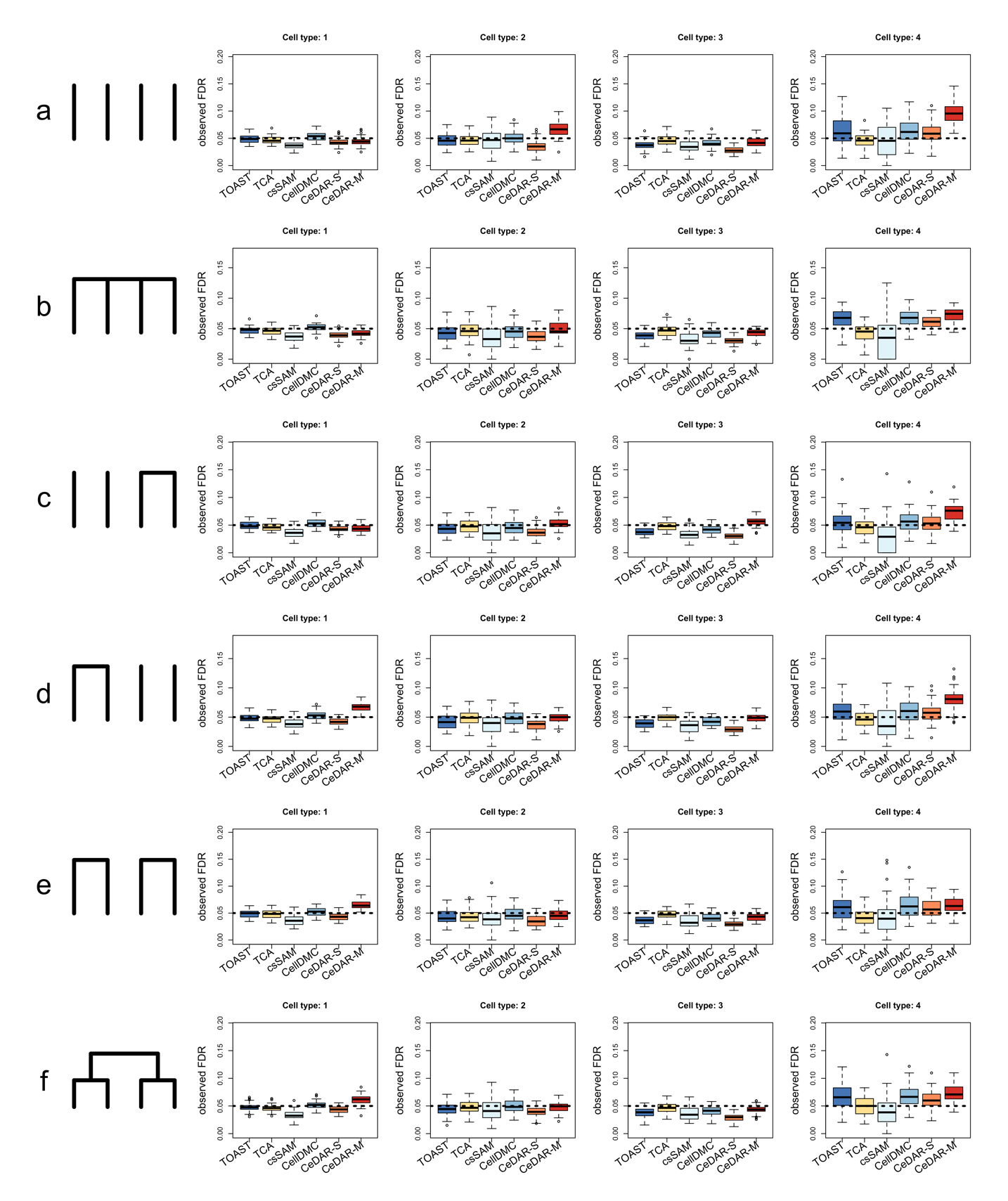


Figure S2: Observed FDR under different DE patterns (strong correlation). DE genes were defined with rule: FDR < 0.05 (TOAST, TCA, csSAM, CellDMC); posterior probability of DE > 0.95 (CeDAR-M, CeDAR-S). Observed FDR of 50 simulations were summarized in box plot.


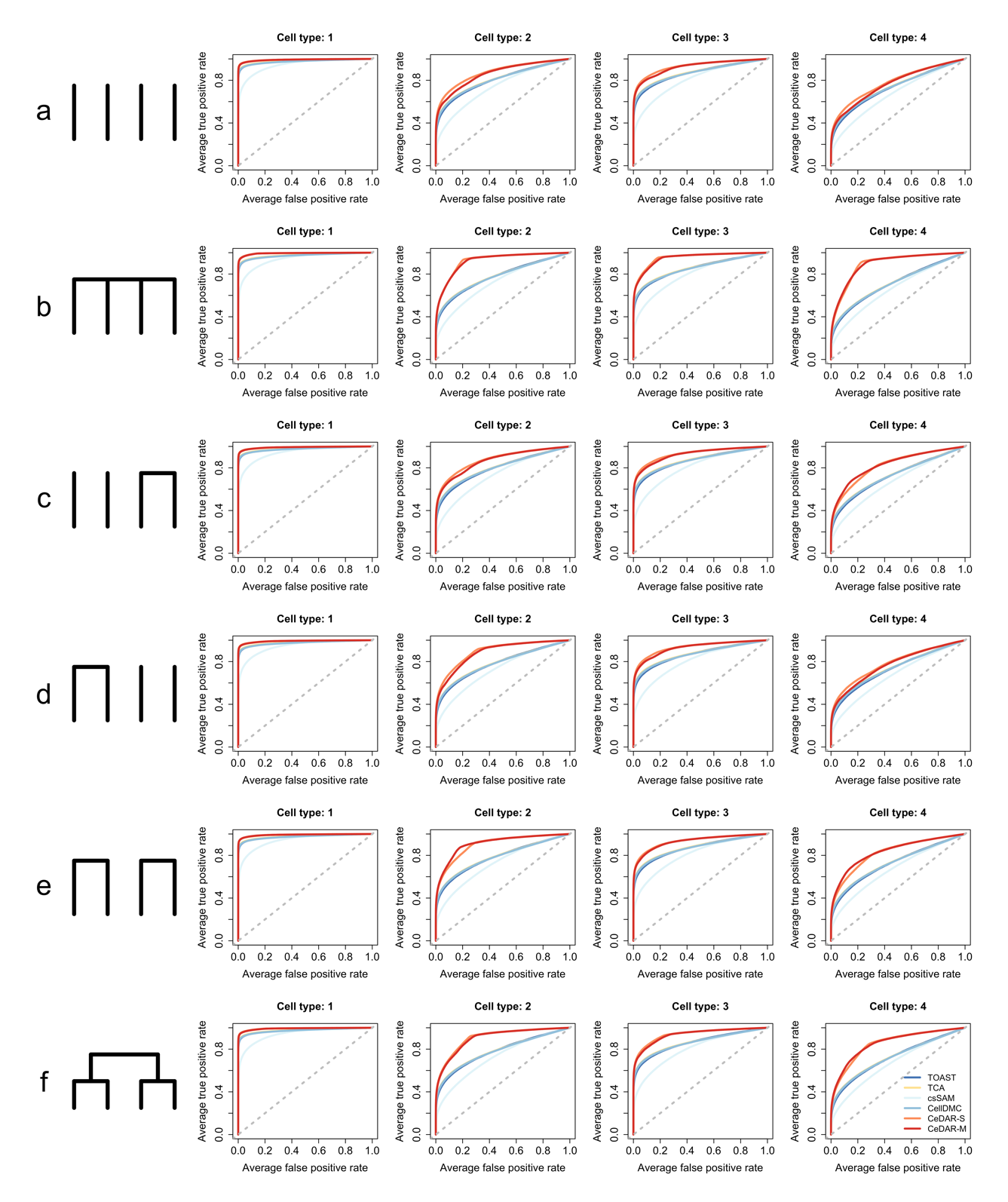


Figure S3: ROC curves under different DE patterns (weak correlation). The simulation mimics a two-group comparison based on bulk microarray gene expression – a mixture of four common blood immune cell types (1: Neutrophils, 2: Monocytes, 3: CD4+, 4: CD8+ cells) under six different DE patterns (a: all cell types are independent; b: all cell types are correlated under a single layer tree structure; c: only cell types 3 and 4 are correlated; d: only cell types 1 and 2 are correlated; e: cell types 1 and 2 are correlated, and cell types 3 and 4 are correlated; f: all cell types are correlated under a multiple-layer tree structure). Methods under comparison include TOAST, TCA, csSAM, CellDMC, CeDAR-S and CeDAR-M. Reported ROC curves are average results from 50 simulations.


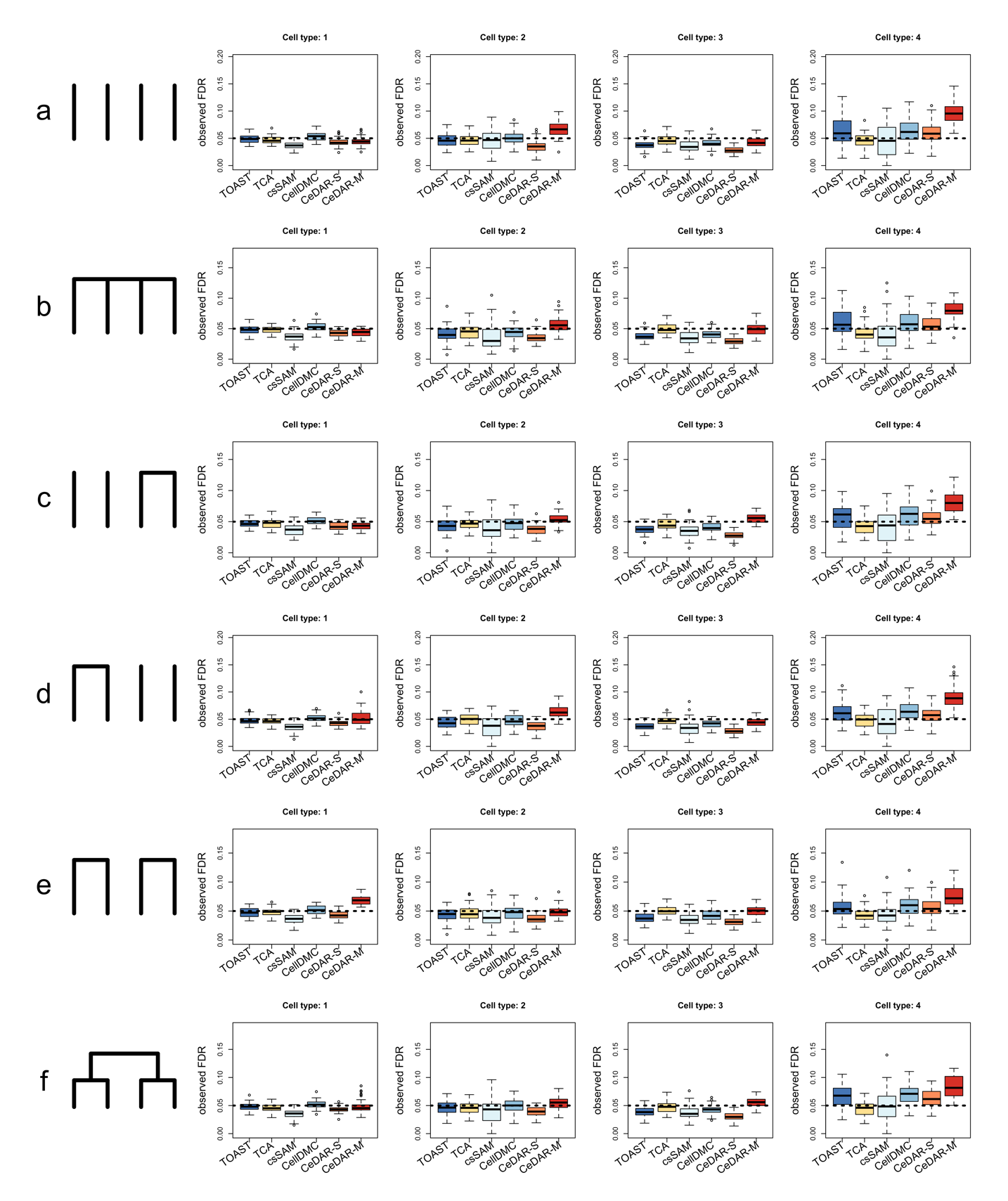


Figure S4: Observed FDR under different DE patterns (weak correlation). DE genes were defined with rule: FDR < 0.05 (TOAST, TCA, csSAM and CellDMC); posterior probability of DE > 0.95 (CeDAR-M, CeDAR-S). Observed FDR of 50 simulations were summarized in box plot.


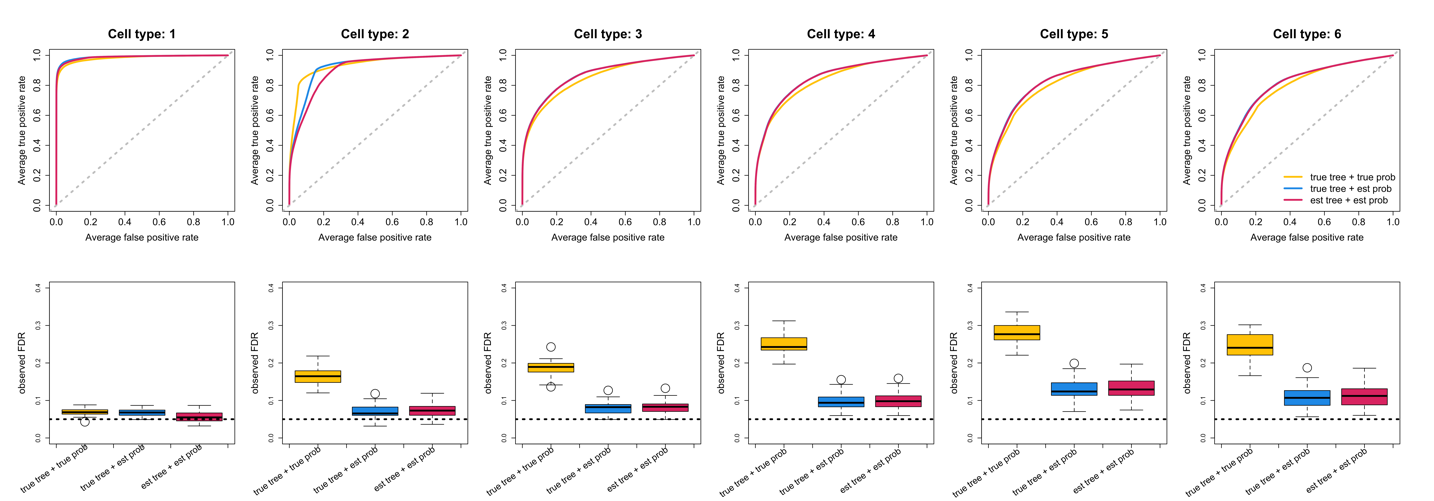


Figure S5: Evaluation of effect on csDE detection performance by using estimated tree structure and estimated prior probability for each node on estimated tree. The upper panel shows ROC curves of csDE analysis by CeDAR-M with true tree + true prior probability (gold), true tree + estimated prior probability (blue), and estimated tree + estimated prior probability (red). The lower panel shows observed FDR of using true/estimated tree structures and prior probabilities. DE genes were defined with rule: posterior probability of DE > 0.95. Observed FDR of 50 simulations were summarized in box plot.


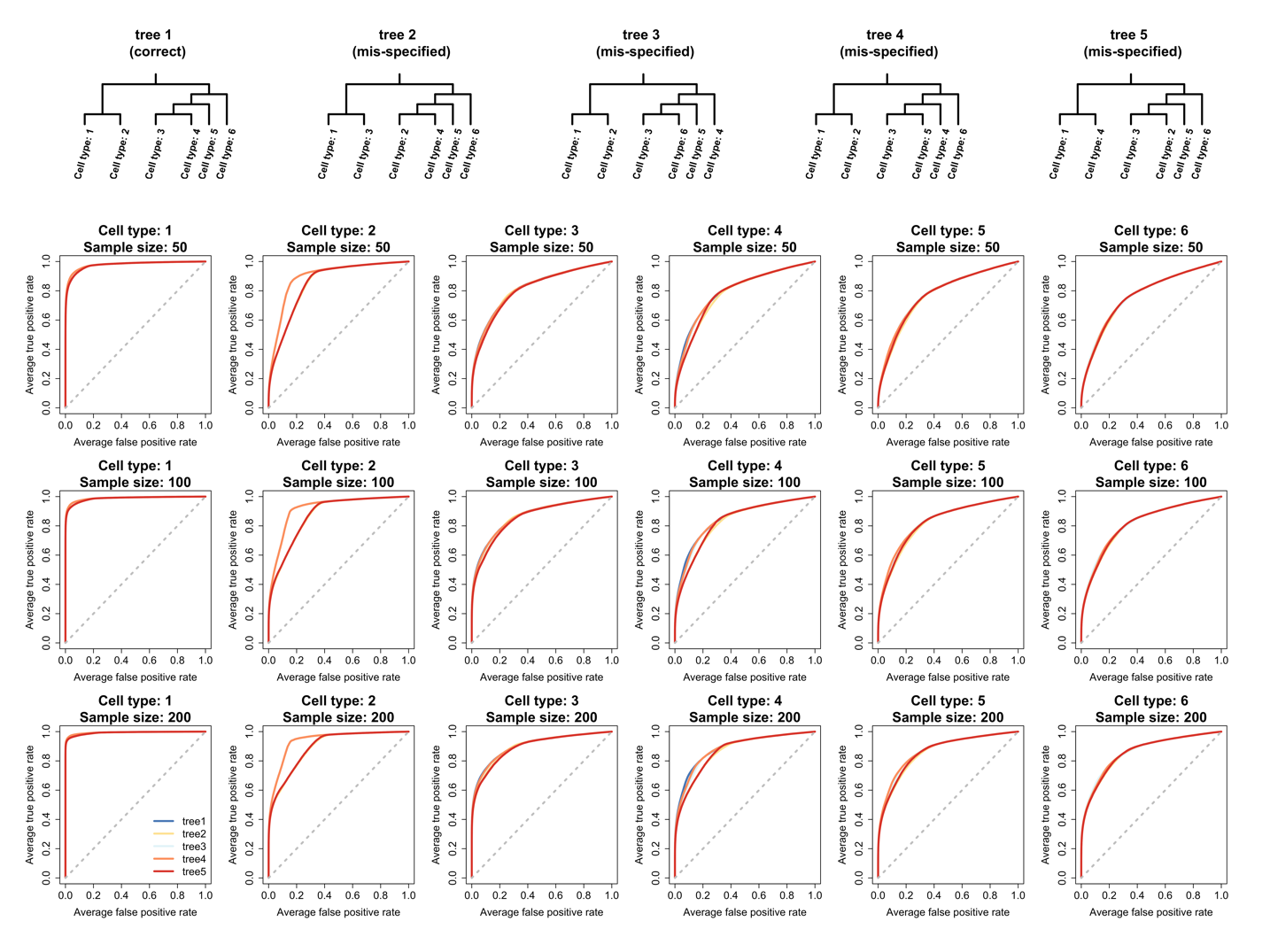


Figure S6: ROC curves with correct/mis-specified tree structure as input of CeDAR-M for cell type specific differential expression analysis. The simulation mimics a two-group comparison based on bulk microarray gene expression – a mixture of six common blood immune cell types (1: Neutrophils, 2: Monocytes, 3: CD4+, 4: CD8+ cells, 5: B cells, 6: NK cells) with different sample sizes per group (50, 100, and 200). “tree 1” is the correct tree structure used to generated simulation data; “tree 2”, “tree 3”, “tree 4” and “tree 5” are mis-specified tree structures by switching cell type 2 with cell type 3, and by switching cell type 4 with cell type 2/5/6, which are used for evaluating impact of mis-specified tree structure. Reported ROC curves are average results from 50 simulations.


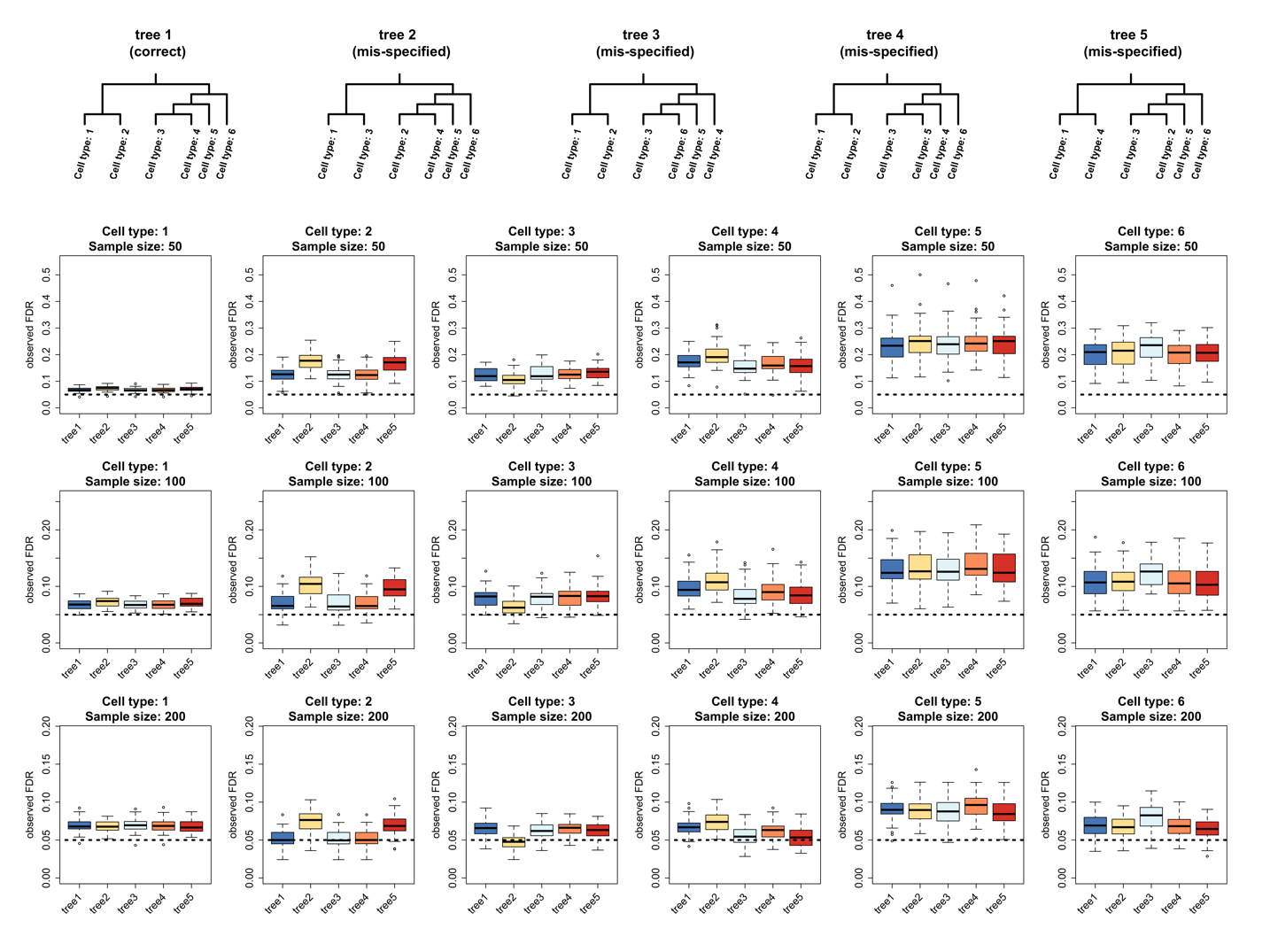


Figure S7 Observed FDR with correct/mis-specified tree structure as input of CeDAR-M for cell type specific differential expression analysis. The simulation mimics a two-group comparison based on bulk microarray gene expression – a mixture of six common blood immune cell types (1: Neutrophils, 2: Monocytes, 3: CD4+, 4: CD8+ cells, 5: B cells, 6: NK cells) with different sample sizes per group (50, 100, and 200). “tree 1” is the correct tree structure used to generated simulation data; “tree 2”, “tree 3”, “tree 4” and “tree 5” are mis-specified tree structures by switching cell type 2 with cell type 3, and by switching cell type 4 with cell type 2/5/6, which are used for evaluating impact of mis-specified tree structure. Reported observed FDR values are average results from 50 simulations.


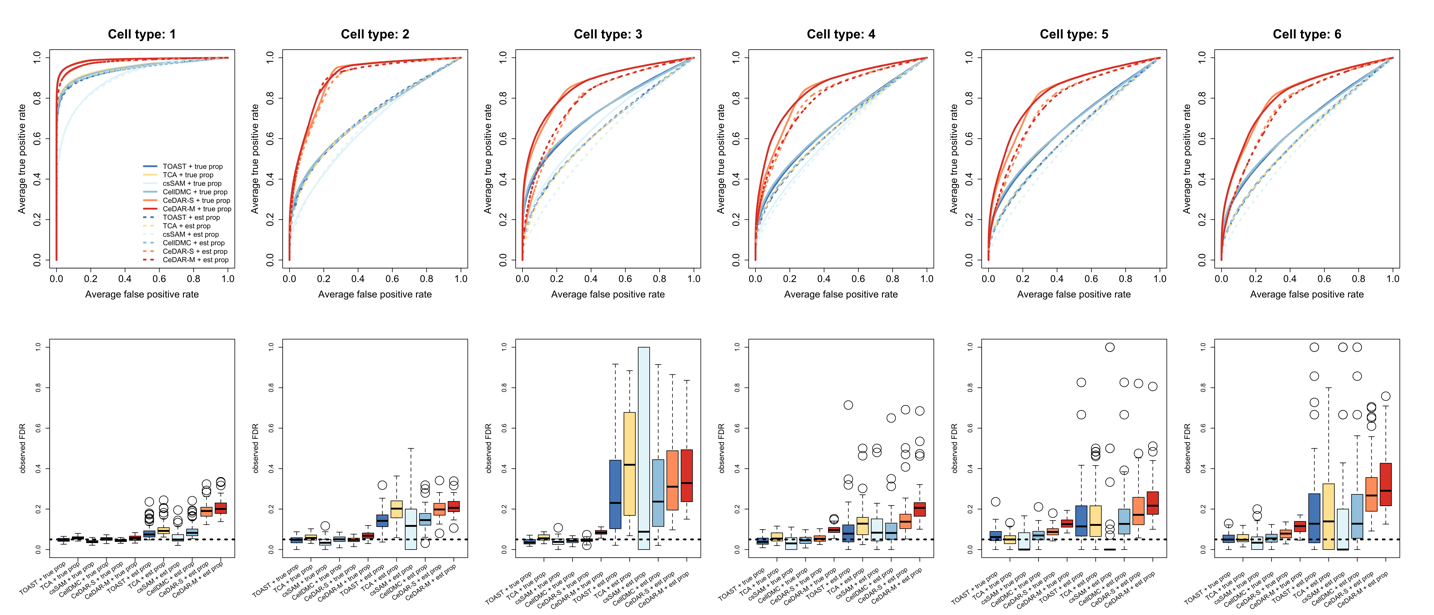


Figure S8: Evaluation of effect on csDE detection performance by using estimated proportion. The upper panel shows ROC curves of four methods with either true proportion (solid line) or estimated proportion (dashed line). The lower panel shows observed FDR of four methods with either true proportion (left four) or estimated proportion (right four). DE genes were defined with rule: FDR < 0.05 (TOAST, TCA, csSAM and CellDMC); posterior probability of DE > 0.95 (CeDAR-M, CeDAR-S). Observed FDR of 50 simulations were summarized in box plot.


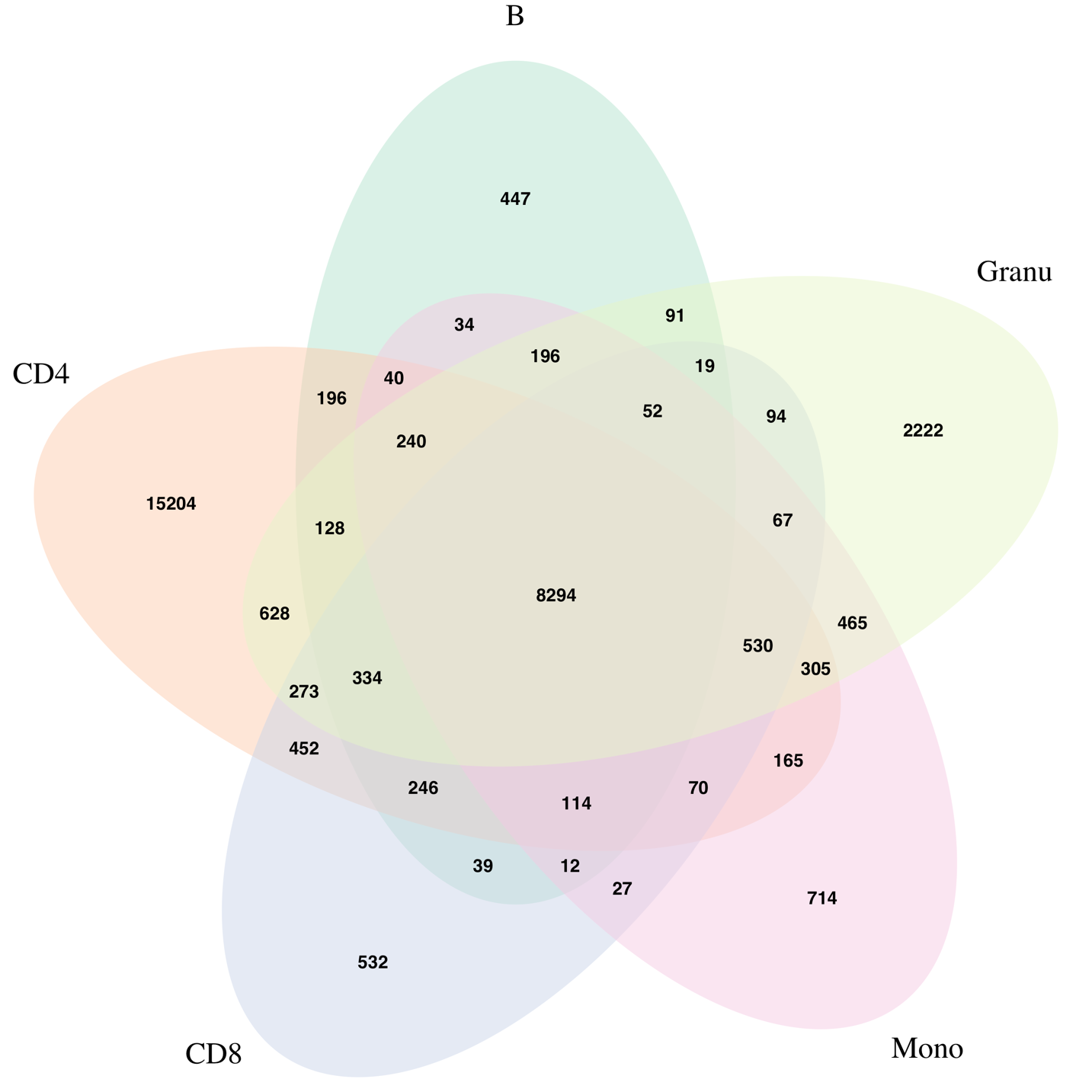


Figure S9: overlap of DMCs detected in pure cell types for data set GSE166844. DMCs in five cell types (granulocytes, monocytes, CD4, CD8 and B cells) were defined with rule FDR < 0.01.


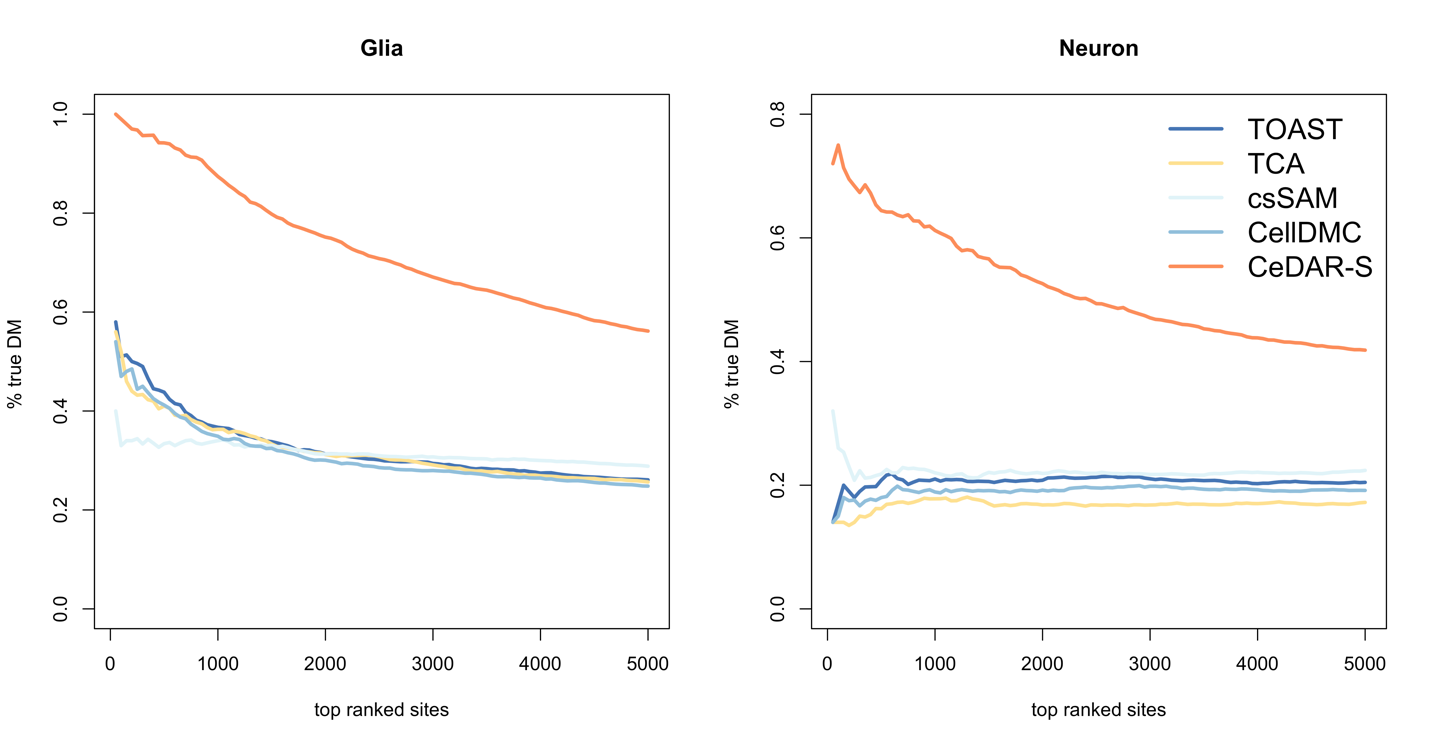


Figure S10: Accuracy of detecting csDM associated with Down syndrome (DS) in human frontal cortex grey matter methylation data. The human frontal cortex grey matter methylation dataset (GEO accession number: GSE74486) contains both bulk samples from frontal cortex grey matter and pure cell type samples of glia and neuron cells derived by FACS. The csDM sites associated with disease DS were identified between 14 DS and 8 normal bulk samples using TOAST, TCA, csSAM, CellDMC, and CeDAR-S. The accuracy was evaluated by TDR curves.


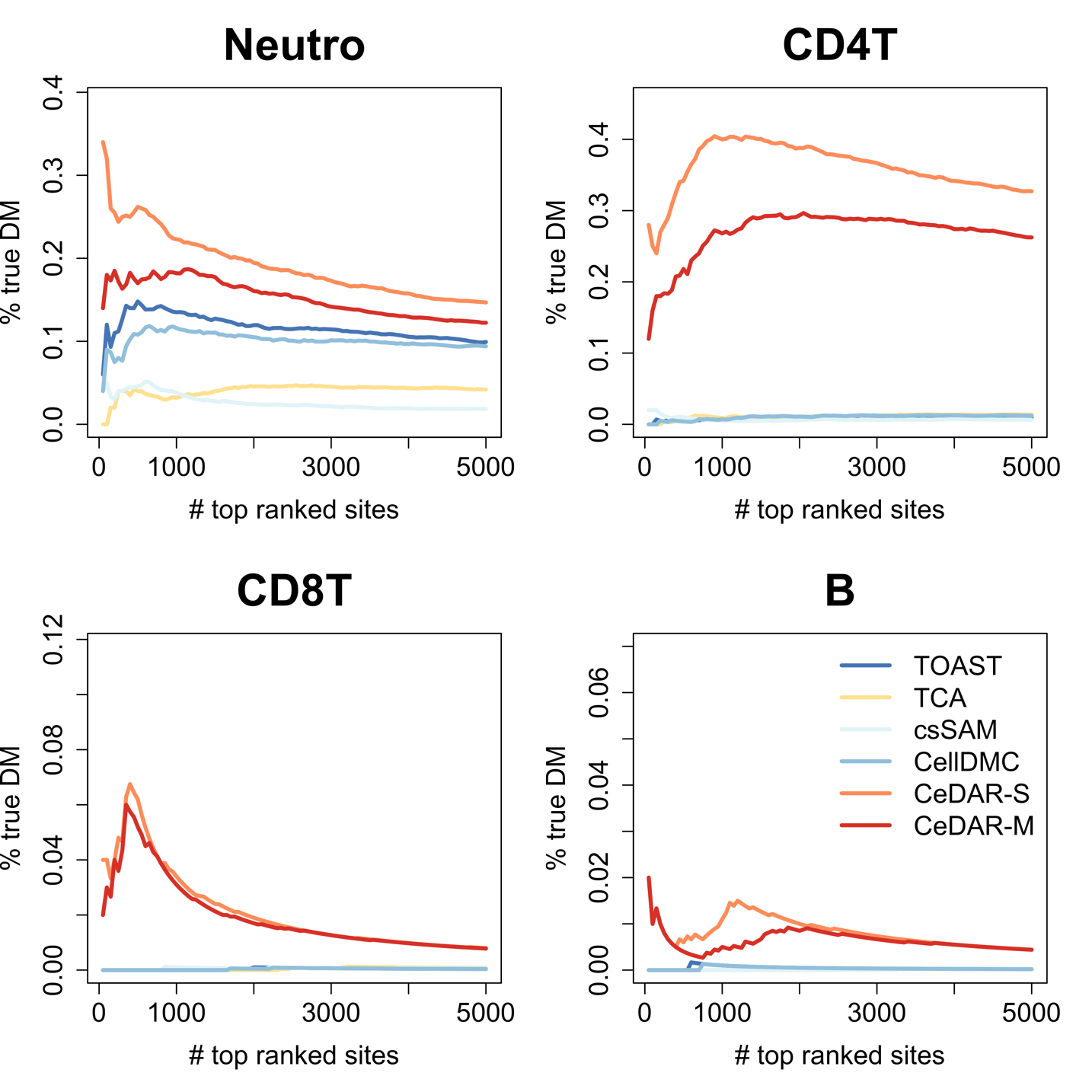


Figure S11: Accuracy of detecting csDM associated with Systemic Lupus Erythematosus (SLE) in human whole blood methylation data. The human whole blood methylation dataset (GEO accession number: GSE118144) contains both bulk samples from whole blood and pure cell type samples of neutrophils, CD8, CD4, and B cells derived by FACS. The csDM sites associated with disease SLE were identified between 16 SLE and 13 normal bulk samples using TOAST, TCA, csSAM, CellDMC, CeDAR-S and CeDAR-M. The accuracy was evaluated by TDR curves.


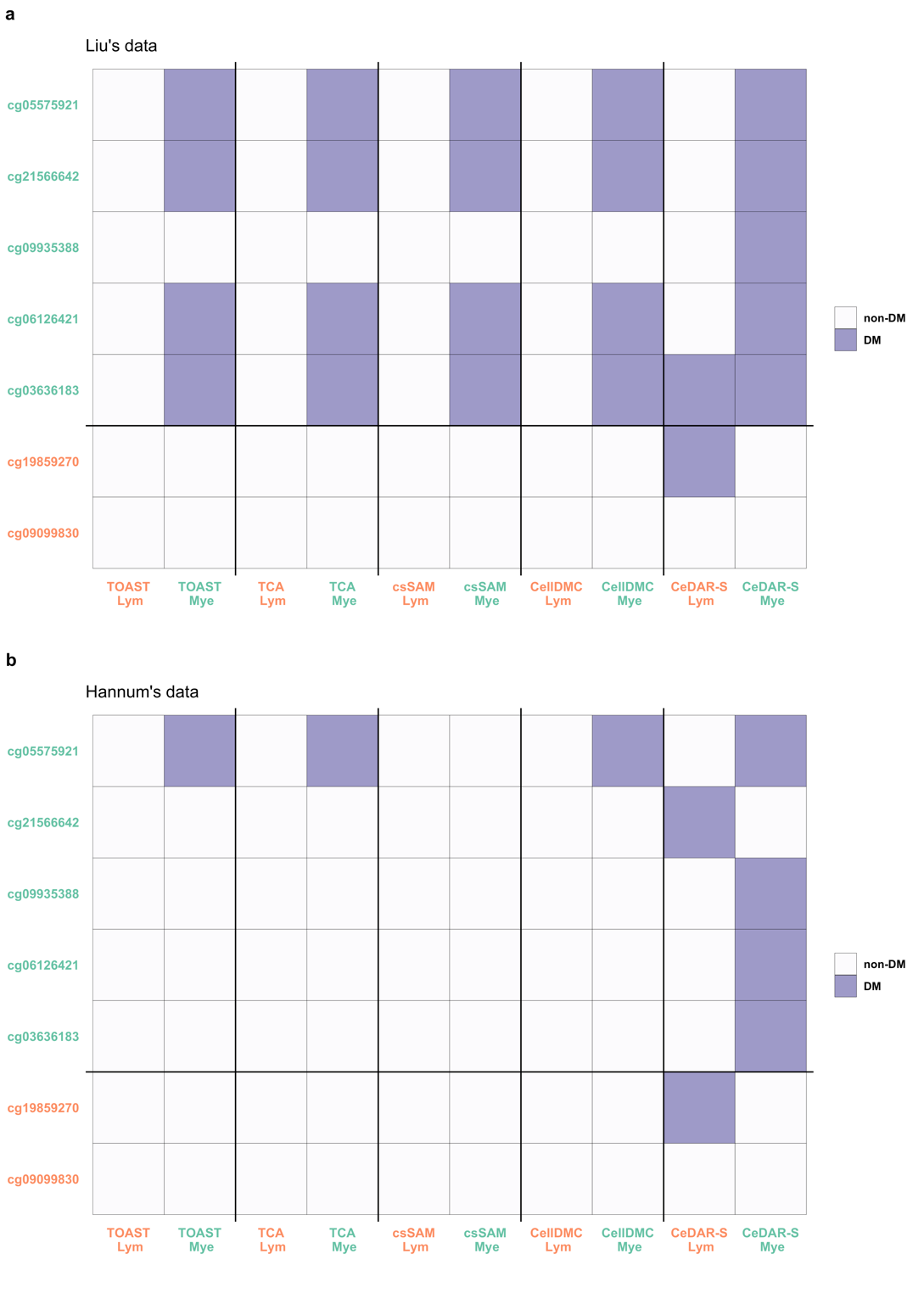


Figure S12: Cell type specific DMC result associated with smoking status for blood methylation data. Examination of TOAST, TCA and CeDAR-S in identifying csDMCs of Lymphoid (Lym) and myeloid (Mye) cells in (a) Liu’s DNA methylation data (GSE42861), and (b) Hannum’s DNA methylation data (GSE40279). Five smoking associated Mye-specific DMCs (cg05575921, cg21566642, cg09935388, cg06126421, and cg03636183) and two Lym-specific DMCs (cg19859270 and cg09099830) used for evaluation were reported by Su et. al. The csDMCs were called by FDR $< 0.05$ for TOAST, TCA, csSAM and CellDMC; by posterior probability of DM $>0.95$ for CeDAR-S.

Table S1: Evaluation (AUC-ROC, AUC-PR, MCC and observed FDR) of different methods with correlated DE states among cell types under various sample sizes per group for cell type specific differential expression analyses.

| **Sample size** | **Methods** | **Cell type 1** | | | | **Cell type 2** | | | | **Cell type 3** | | | | **Cell type 4** | | | | **Cell type 5** | | | | **Cell type 6** | | | |
| --- | --- | --- | --- | --- | --- | --- | --- | --- | --- | --- | --- | --- | --- | --- | --- | --- | --- | --- | --- | --- | --- | --- | --- | --- | --- |
|  |  | **ROC**  **AUC** | **PR**  **AUC** | **MCC** | **FDR** | **ROC**  **AUC** | **PR**  **AUC** | **MCC** | **FDR** | **ROC**  **AUC** | **PR**  **AUC** | **MCC** | **FDR** | **ROC**  **AUC** | **PR**  **AUC** | **MCC** | **FDR** | **ROC**  **AUC** | **PR**  **AUC** | **MCC** | **FDR** | **ROC**  **AUC** | **PR**  **AUC** | **MCC** | **FDR** |
| **50** | **TOAST** | 0.897 | 0.732 | 0.600 | 0.049 | 0.642 | 0.264 | 0.160 | 0.048 | 0.664 | 0.304 | 0.202 | 0.042 | 0.616 | 0.226 | 0.135 | 0.047 | 0.605 | 0.205 | 0.107 | 0.095 | 0.609 | 0.204 | 0.087 | 0.077 |
|  | **TCA** | 0.897 | 0.736 | 0.638 | 0.093 | 0.640 | 0.269 | 0.187 | 0.099 | 0.664 | 0.310 | 0.232 | 0.091 | 0.615 | 0.229 | 0.151 | 0.095 | 0.605 | 0.209 | 0.118 | 0.119 | 0.607 | 0.205 | 0.099 | 0.170 |
|  | **csSAM** | 0.841 | 0.528 | 0.315 | 0.036 | 0.608 | 0.198 | 0.084 | 0.033 | 0.625 | 0.227 | 0.137 | 0.039 | 0.587 | 0.180 | 0.086 | 0.043 | 0.580 | 0.164 | 0.037 | 0.046 | 0.580 | 0.161 | 0.060 | 0.042 |
|  | **CellDMC** | 0.898 | 0.746 | 0.632 | 0.054 | 0.641 | 0.275 | 0.180 | 0.049 | 0.666 | 0.317 | 0.219 | 0.048 | 0.616 | 0.235 | 0.142 | 0.050 | 0.606 | 0.213 | 0.114 | 0.107 | 0.610 | 0.212 | 0.097 | 0.086 |
|  | **CeDAR-S** | 0.979 | 0.921 | 0.829 | 0.050 | 0.877 | 0.487 | 0.274 | 0.089 | 0.821 | 0.477 | 0.320 | 0.080 | 0.797 | 0.379 | 0.216 | 0.131 | 0.778 | 0.342 | 0.191 | 0.184 | 0.771 | 0.338 | 0.182 | 0.183 |
|  | **CeDAR-M** | 0.977 | 0.913 | 0.814 | 0.055 | 0.861 | 0.458 | 0.286 | 0.139 | 0.818 | 0.483 | 0.355 | 0.132 | 0.800 | 0.404 | 0.252 | 0.188 | 0.776 | 0.351 | 0.215 | 0.243 | 0.766 | 0.335 | 0.198 | 0.247 |
| **100** | **TOAST** | 0.950 | 0.872 | 0.797 | 0.047 | 0.704 | 0.381 | 0.303 | 0.046 | 0.728 | 0.430 | 0.351 | 0.038 | 0.670 | 0.323 | 0.246 | 0.044 | 0.656 | 0.293 | 0.213 | 0.070 | 0.671 | 0.313 | 0.216 | 0.054 |
|  | **TCA** | 0.952 | 0.882 | 0.815 | 0.060 | 0.706 | 0.396 | 0.332 | 0.059 | 0.732 | 0.448 | 0.384 | 0.057 | 0.672 | 0.337 | 0.272 | 0.062 | 0.659 | 0.309 | 0.233 | 0.062 | 0.673 | 0.327 | 0.240 | 0.055 |
|  | **csSAM** | 0.905 | 0.700 | 0.537 | 0.040 | 0.662 | 0.270 | 0.173 | 0.043 | 0.675 | 0.301 | 0.224 | 0.037 | 0.628 | 0.236 | 0.159 | 0.041 | 0.617 | 0.213 | 0.091 | 0.037 | 0.626 | 0.220 | 0.115 | 0.037 |
|  | **CellDMC** | 0.949 | 0.878 | 0.812 | 0.052 | 0.704 | 0.395 | 0.327 | 0.048 | 0.731 | 0.448 | 0.376 | 0.043 | 0.672 | 0.338 | 0.265 | 0.048 | 0.659 | 0.308 | 0.232 | 0.068 | 0.673 | 0.330 | 0.240 | 0.056 |
|  | **CeDAR-S** | 0.988 | 0.958 | 0.901 | 0.045 | 0.897 | 0.575 | 0.388 | 0.055 | 0.865 | 0.595 | 0.444 | 0.043 | 0.837 | 0.480 | 0.320 | 0.063 | 0.820 | 0.441 | 0.291 | 0.095 | 0.820 | 0.460 | 0.305 | 0.074 |
|  | **CeDAR-M** | 0.988 | 0.958 | 0.896 | 0.056 | 0.899 | 0.585 | 0.402 | 0.076 | 0.866 | 0.614 | 0.489 | 0.081 | 0.847 | 0.526 | 0.368 | 0.100 | 0.826 | 0.466 | 0.321 | 0.132 | 0.820 | 0.468 | 0.327 | 0.102 |
| **200** | **TOAST** | 0.975 | 0.940 | 0.891 | 0.050 | 0.771 | 0.513 | 0.446 | 0.045 | 0.795 | 0.565 | 0.496 | 0.039 | 0.734 | 0.448 | 0.380 | 0.042 | 0.722 | 0.416 | 0.350 | 0.058 | 0.733 | 0.436 | 0.364 | 0.048 |
|  | **TCA** | 0.978 | 0.947 | 0.903 | 0.050 | 0.775 | 0.534 | 0.478 | 0.050 | 0.800 | 0.588 | 0.531 | 0.048 | 0.739 | 0.469 | 0.414 | 0.051 | 0.727 | 0.443 | 0.381 | 0.047 | 0.736 | 0.457 | 0.392 | 0.053 |
|  | **csSAM** | 0.944 | 0.818 | 0.703 | 0.035 | 0.721 | 0.365 | 0.274 | 0.041 | 0.737 | 0.402 | 0.325 | 0.042 | 0.681 | 0.317 | 0.249 | 0.039 | 0.671 | 0.292 | 0.180 | 0.039 | 0.679 | 0.297 | 0.203 | 0.034 |
|  | **CellDMC** | 0.974 | 0.941 | 0.897 | 0.054 | 0.771 | 0.528 | 0.470 | 0.049 | 0.797 | 0.582 | 0.520 | 0.043 | 0.736 | 0.465 | 0.404 | 0.045 | 0.724 | 0.435 | 0.374 | 0.062 | 0.735 | 0.454 | 0.390 | 0.051 |
|  | **CeDAR-S** | 0.993 | 0.976 | 0.936 | 0.042 | 0.916 | 0.668 | 0.514 | 0.038 | 0.901 | 0.699 | 0.570 | 0.032 | 0.873 | 0.590 | 0.444 | 0.042 | 0.860 | 0.557 | 0.417 | 0.059 | 0.860 | 0.571 | 0.434 | 0.046 |
|  | **CeDAR-M** | 0.994 | 0.980 | 0.929 | 0.071 | 0.940 | 0.723 | 0.538 | 0.055 | 0.905 | 0.723 | 0.617 | 0.065 | 0.887 | 0.642 | 0.493 | 0.072 | 0.870 | 0.587 | 0.448 | 0.086 | 0.863 | 0.583 | 0.452 | 0.065 |

The reported metrics (AUC-ROC, AUC-PR, MCC, and observed FDR) are average of 50 simulations. For TOAST, TCA, csSAM, and CellDMC, the MCC and observed FDR were derived by calling DE with estimated FDR < 0.05; for CeDAR-S and CeDAR-M, the MCC and observed FDR were derived by calling DE with estimated posterior probability of DE > 0.95

Table S2: Evaluation (AUC-ROC, AUC-PR, MCC and observed FDR) of different methods under various DE state patterns for cell type specific differential expression analyses (corresponding to Figure 4: strong correlation).

| **Fig 4 panel** | **Methods** | **Cell type 1** | | | | **Cell type 2** | | | | **Cell type 3** | | | | **Cell type 4** | | | |  |
| --- | --- | --- | --- | --- | --- | --- | --- | --- | --- | --- | --- | --- | --- | --- | --- | --- | --- | --- |
|  |  | **ROC**  **AUC** | **PR**  **AUC** | **MCC** | **FDR** | **ROC**  **AUC** | **PR**  **AUC** | **MCC** | **FDR** | **ROC**  **AUC** | **PR**  **AUC** | **MCC** | **FDR** | **ROC**  **AUC** | **PR**  **AUC** | **MCC** | **FDR** | |
| **a** | **TOAST** | 0.976 | 0.943 | 0.896 | 0.049 | 0.803 | 0.569 | 0.489 | 0.046 | 0.868 | 0.713 | 0.647 | 0.037 | 0.732 | 0.434 | 0.362 | 0.065 | |
|  | **TCA** | 0.978 | 0.949 | 0.907 | 0.047 | 0.809 | 0.594 | 0.523 | 0.046 | 0.874 | 0.736 | 0.680 | 0.047 | 0.739 | 0.464 | 0.397 | 0.046 | |
|  | **csSAM** | 0.945 | 0.821 | 0.706 | 0.037 | 0.740 | 0.392 | 0.293 | 0.044 | 0.807 | 0.526 | 0.435 | 0.036 | 0.675 | 0.297 | 0.182 | 0.044 | |
|  | **CellDMC** | 0.975 | 0.944 | 0.901 | 0.053 | 0.806 | 0.588 | 0.517 | 0.050 | 0.87 | 0.729 | 0.673 | 0.041 | 0.736 | 0.456 | 0.393 | 0.065 | |
|  | **CeDAR-S** | 0.992 | 0.976 | 0.937 | 0.043 | 0.875 | 0.681 | 0.566 | 0.036 | 0.932 | 0.820 | 0.724 | 0.028 | 0.782 | 0.514 | 0.430 | 0.061 | |
|  | **CeDAR-M** | 0.991 | 0.974 | 0.933 | 0.045 | 0.861 | 0.650 | 0.575 | 0.067 | 0.925 | 0.804 | 0.728 | 0.043 | 0.768 | 0.488 | 0.439 | 0.096 | |
| **b** | **TOAST** | 0.970 | 0.930 | 0.882 | 0.047 | 0.761 | 0.491 | 0.421 | 0.042 | 0.836 | 0.651 | 0.589 | 0.038 | 0.698 | 0.373 | 0.307 | 0.065 | |
|  | **TCA** | 0.972 | 0.937 | 0.894 | 0.046 | 0.763 | 0.510 | 0.448 | 0.048 | 0.839 | 0.669 | 0.618 | 0.047 | 0.701 | 0.393 | 0.332 | 0.043 | |
|  | **csSAM** | 0.940 | 0.810 | 0.694 | 0.037 | 0.717 | 0.353 | 0.256 | 0.036 | 0.789 | 0.494 | 0.405 | 0.033 | 0.660 | 0.269 | 0.148 | 0.037 | |
|  | **CellDMC** | 0.969 | 0.931 | 0.887 | 0.052 | 0.760 | 0.504 | 0.444 | 0.046 | 0.834 | 0.662 | 0.610 | 0.043 | 0.697 | 0.386 | 0.329 | 0.067 | |
|  | **CeDAR-S** | 0.997 | 0.984 | 0.934 | 0.040 | 0.961 | 0.756 | 0.508 | 0.038 | 0.976 | 0.860 | 0.674 | 0.030 | 0.947 | 0.661 | 0.388 | 0.061 | |
|  | **CeDAR-M** | 0.996 | 0.983 | 0.932 | 0.043 | 0.963 | 0.778 | 0.541 | 0.049 | 0.976 | 0.866 | 0.700 | 0.043 | 0.953 | 0.703 | 0.425 | 0.073 | |
| **c** | **TOAST** | 0.977 | 0.944 | 0.895 | 0.049 | 0.796 | 0.558 | 0.479 | 0.043 | 0.868 | 0.711 | 0.645 | 0.039 | 0.721 | 0.412 | 0.337 | 0.055 | |
|  | **TCA** | 0.979 | 0.950 | 0.907 | 0.047 | 0.802 | 0.583 | 0.514 | 0.047 | 0.873 | 0.733 | 0.676 | 0.048 | 0.725 | 0.436 | 0.366 | 0.046 | |
|  | **csSAM** | 0.945 | 0.820 | 0.704 | 0.036 | 0.733 | 0.384 | 0.284 | 0.038 | 0.807 | 0.526 | 0.435 | 0.034 | 0.671 | 0.287 | 0.177 | 0.031 | |
|  | **CellDMC** | 0.976 | 0.945 | 0.900 | 0.053 | 0.799 | 0.578 | 0.509 | 0.047 | 0.869 | 0.726 | 0.669 | 0.042 | 0.723 | 0.429 | 0.363 | 0.057 | |
|  | **CeDAR-S** | 0.992 | 0.978 | 0.939 | 0.043 | 0.878 | 0.680 | 0.558 | 0.037 | 0.931 | 0.817 | 0.721 | 0.030 | 0.867 | 0.557 | 0.402 | 0.054 | |
|  | **CeDAR-M** | 0.992 | 0.976 | 0.935 | 0.044 | 0.869 | 0.662 | 0.566 | 0.053 | 0.930 | 0.819 | 0.745 | 0.056 | 0.895 | 0.638 | 0.448 | 0.073 | |
| **d** | **TOAST** | 0.974 | 0.939 | 0.891 | 0.048 | 0.767 | 0.508 | 0.437 | 0.043 | 0.870 | 0.713 | 0.646 | 0.039 | 0.731 | 0.433 | 0.361 | 0.059 | |
|  | **TCA** | 0.976 | 0.946 | 0.902 | 0.047 | 0.769 | 0.525 | 0.465 | 0.048 | 0.875 | 0.737 | 0.679 | 0.049 | 0.739 | 0.461 | 0.392 | 0.047 | |
|  | **csSAM** | 0.944 | 0.817 | 0.700 | 0.039 | 0.719 | 0.362 | 0.271 | 0.038 | 0.807 | 0.527 | 0.435 | 0.035 | 0.675 | 0.297 | 0.188 | 0.041 | |
|  | **CellDMC** | 0.974 | 0.940 | 0.896 | 0.053 | 0.767 | 0.521 | 0.461 | 0.048 | 0.872 | 0.730 | 0.672 | 0.042 | 0.736 | 0.454 | 0.389 | 0.061 | |
|  | **CeDAR-S** | 0.992 | 0.974 | 0.933 | 0.042 | 0.922 | 0.673 | 0.505 | 0.037 | 0.939 | 0.831 | 0.727 | 0.030 | 0.787 | 0.519 | 0.431 | 0.057 | |
|  | **CeDAR-M** | 0.993 | 0.980 | 0.931 | 0.068 | 0.950 | 0.743 | 0.532 | 0.048 | 0.932 | 0.809 | 0.727 | 0.048 | 0.778 | 0.499 | 0.436 | 0.08 | |
| **e** | **TOAST** | 0.974 | 0.940 | 0.893 | 0.048 | 0.768 | 0.510 | 0.439 | 0.043 | 0.868 | 0.712 | 0.646 | 0.037 | 0.719 | 0.409 | 0.338 | 0.062 | |
|  | **TCA** | 0.976 | 0.945 | 0.903 | 0.048 | 0.770 | 0.527 | 0.468 | 0.044 | 0.874 | 0.735 | 0.677 | 0.048 | 0.724 | 0.434 | 0.367 | 0.044 | |
|  | **csSAM** | 0.944 | 0.819 | 0.705 | 0.037 | 0.721 | 0.366 | 0.278 | 0.039 | 0.808 | 0.526 | 0.432 | 0.035 | 0.667 | 0.283 | 0.168 | 0.041 | |
|  | **CellDMC** | 0.974 | 0.941 | 0.897 | 0.053 | 0.767 | 0.522 | 0.463 | 0.048 | 0.870 | 0.728 | 0.670 | 0.041 | 0.721 | 0.427 | 0.361 | 0.064 | |
|  | **CeDAR-S** | 0.993 | 0.977 | 0.935 | 0.043 | 0.932 | 0.695 | 0.512 | 0.037 | 0.938 | 0.829 | 0.726 | 0.030 | 0.881 | 0.578 | 0.405 | 0.059 | |
|  | **CeDAR-M** | 0.994 | 0.982 | 0.933 | 0.065 | 0.954 | 0.755 | 0.537 | 0.046 | 0.940 | 0.836 | 0.749 | 0.043 | 0.907 | 0.665 | 0.449 | 0.065 | |
| **f** | **TOAST** | 0.973 | 0.936 | 0.889 | 0.047 | 0.762 | 0.497 | 0.428 | 0.045 | 0.852 | 0.682 | 0.619 | 0.038 | 0.711 | 0.394 | 0.325 | 0.067 | |
|  | **TCA** | 0.975 | 0.942 | 0.900 | 0.046 | 0.765 | 0.515 | 0.454 | 0.048 | 0.856 | 0.701 | 0.649 | 0.047 | 0.714 | 0.417 | 0.353 | 0.048 | |
|  | **csSAM** | 0.944 | 0.817 | 0.701 | 0.033 | 0.716 | 0.355 | 0.259 | 0.044 | 0.799 | 0.511 | 0.423 | 0.036 | 0.666 | 0.277 | 0.156 | 0.040 | |
|  | **CellDMC** | 0.972 | 0.937 | 0.893 | 0.052 | 0.761 | 0.509 | 0.450 | 0.049 | 0.852 | 0.695 | 0.641 | 0.041 | 0.711 | 0.409 | 0.348 | 0.070 | |
|  | **CeDAR-S** | 0.995 | 0.978 | 0.932 | 0.043 | 0.946 | 0.717 | 0.504 | 0.040 | 0.956 | 0.835 | 0.699 | 0.029 | 0.914 | 0.613 | 0.401 | 0.061 | |
|  | **CeDAR-M** | 0.995 | 0.982 | 0.931 | 0.061 | 0.957 | 0.754 | 0.522 | 0.048 | 0.955 | 0.838 | 0.721 | 0.044 | 0.928 | 0.677 | 0.435 | 0.072 | |

There are six different DE patterns corresponding to six panels in Figure 4 (a: all cell types are independent; b: all cell types are correlated under a single layer tree structure; c: only cell types 3 and 4 are correlated; d: only cell types 1 and 2 are correlated; e: cell types 1 and 2 are correlated, and cell types 3 and 4 are correlated; f: all cell types are correlated under a multiple-layer tree structure) The reported metrics (AUC-ROC, AUC-PR, MCC, and observed FDR) are average of 50 simulations. For TOAST, TCA, csSAM, and CellDMC the observed FDR was derived by calling DE with estimated FDR < 0.05; for CeDAR-S and CeDAR-M, the MCC and observed FDR was derived by calling DE with estimated posterior probability of DE > 0.95.

Table S3: Evaluation (AUC-ROC, AUC-PR, MCC and observed FDR) of different methods under various DE state patterns for cell type specific differential expression analyses (corresponding to Figure S3: weak correlation).

| **Fig S3 panel** | **Methods** | **Cell type 1** | | | | **Cell type 2** | | | | **Cell type 3** | | | | **Cell type 4** | | | |
| --- | --- | --- | --- | --- | --- | --- | --- | --- | --- | --- | --- | --- | --- | --- | --- | --- | --- |
|  |  | **ROC**  **AUC** | **PR**  **AUC** | **MCC** | **FDR** | **ROC**  **AUC** | **PR**  **AUC** | **MCC** | **FDR** | **ROC**  **AUC** | **PR**  **AUC** | **MCC** | **FDR** | **ROC**  **AUC** | **PR**  **AUC** | **MCC** | **FDR** |
| **a** | **TOAST** | 0.976 | 0.943 | 0.896 | 0.049 | 0.803 | 0.569 | 0.489 | 0.046 | 0.868 | 0.713 | 0.647 | 0.037 | 0.732 | 0.434 | 0.362 | 0.065 |
|  | **TCA** | 0.978 | 0.949 | 0.907 | 0.047 | 0.809 | 0.594 | 0.523 | 0.046 | 0.874 | 0.736 | 0.680 | 0.047 | 0.739 | 0.464 | 0.397 | 0.046 |
|  | **csSAM** | 0.945 | 0.821 | 0.706 | 0.037 | 0.740 | 0.392 | 0.293 | 0.044 | 0.807 | 0.526 | 0.435 | 0.036 | 0.675 | 0.297 | 0.182 | 0.044 |
|  | **CellDMC** | 0.975 | 0.944 | 0.901 | 0.053 | 0.806 | 0.588 | 0.517 | 0.050 | 0.870 | 0.729 | 0.673 | 0.041 | 0.736 | 0.456 | 0.393 | 0.065 |
|  | **CeDAR-S** | 0.992 | 0.976 | 0.937 | 0.043 | 0.875 | 0.681 | 0.566 | 0.036 | 0.932 | 0.820 | 0.724 | 0.028 | 0.782 | 0.514 | 0.430 | 0.061 |
|  | **CeDAR-M** | 0.991 | 0.974 | 0.933 | 0.045 | 0.861 | 0.650 | 0.575 | 0.067 | 0.925 | 0.804 | 0.728 | 0.043 | 0.768 | 0.488 | 0.439 | 0.096 |
| **b** | **TOAST** | 0.973 | 0.936 | 0.887 | 0.048 | 0.778 | 0.524 | 0.453 | 0.041 | 0.854 | 0.686 | 0.621 | 0.037 | 0.713 | 0.396 | 0.329 | 0.059 |
|  | **TCA** | 0.975 | 0.942 | 0.898 | 0.049 | 0.781 | 0.544 | 0.481 | 0.045 | 0.857 | 0.704 | 0.651 | 0.049 | 0.716 | 0.420 | 0.359 | 0.043 |
|  | **csSAM** | 0.943 | 0.814 | 0.697 | 0.037 | 0.725 | 0.370 | 0.273 | 0.037 | 0.800 | 0.514 | 0.424 | 0.035 | 0.666 | 0.279 | 0.169 | 0.040 |
|  | **CellDMC** | 0.972 | 0.937 | 0.892 | 0.053 | 0.778 | 0.539 | 0.477 | 0.044 | 0.853 | 0.698 | 0.643 | 0.041 | 0.713 | 0.412 | 0.355 | 0.059 |
|  | **CeDAR-S** | 0.994 | 0.977 | 0.932 | 0.043 | 0.926 | 0.708 | 0.528 | 0.036 | 0.954 | 0.829 | 0.698 | 0.030 | 0.895 | 0.589 | 0.401 | 0.056 |
|  | **CeDAR-M** | 0.994 | 0.976 | 0.929 | 0.044 | 0.924 | 0.707 | 0.549 | 0.056 | 0.951 | 0.821 | 0.713 | 0.049 | 0.894 | 0.599 | 0.426 | 0.080 |
| **c** | **TOAST** | 0.976 | 0.942 | 0.895 | 0.047 | 0.797 | 0.560 | 0.484 | 0.044 | 0.868 | 0.711 | 0.647 | 0.038 | 0.726 | 0.423 | 0.352 | 0.057 |
|  | **TCA** | 0.977 | 0.948 | 0.906 | 0.047 | 0.803 | 0.586 | 0.517 | 0.047 | 0.874 | 0.733 | 0.678 | 0.046 | 0.732 | 0.450 | 0.383 | 0.043 |
|  | **csSAM** | 0.945 | 0.820 | 0.702 | 0.037 | 0.734 | 0.386 | 0.286 | 0.039 | 0.809 | 0.529 | 0.438 | 0.035 | 0.673 | 0.292 | 0.177 | 0.041 |
|  | **CellDMC** | 0.975 | 0.943 | 0.900 | 0.052 | 0.800 | 0.580 | 0.511 | 0.047 | 0.870 | 0.726 | 0.671 | 0.041 | 0.729 | 0.442 | 0.378 | 0.060 |
|  | **CeDAR-S** | 0.992 | 0.976 | 0.937 | 0.043 | 0.876 | 0.679 | 0.561 | 0.037 | 0.932 | 0.818 | 0.721 | 0.028 | 0.824 | 0.532 | 0.416 | 0.055 |
|  | **CeDAR-M** | 0.991 | 0.974 | 0.934 | 0.043 | 0.867 | 0.662 | 0.568 | 0.053 | 0.926 | 0.804 | 0.733 | 0.056 | 0.831 | 0.554 | 0.441 | 0.082 |
| **d** | **TOAST** | 0.975 | 0.940 | 0.893 | 0.048 | 0.781 | 0.529 | 0.454 | 0.044 | 0.867 | 0.712 | 0.649 | 0.037 | 0.732 | 0.433 | 0.361 | 0.062 |
|  | **TCA** | 0.977 | 0.947 | 0.905 | 0.046 | 0.786 | 0.551 | 0.486 | 0.048 | 0.873 | 0.735 | 0.681 | 0.047 | 0.74 | 0.462 | 0.394 | 0.047 |
|  | **csSAM** | 0.944 | 0.819 | 0.703 | 0.036 | 0.727 | 0.372 | 0.276 | 0.037 | 0.806 | 0.527 | 0.438 | 0.035 | 0.674 | 0.296 | 0.184 | 0.042 |
|  | **CellDMC** | 0.974 | 0.942 | 0.898 | 0.053 | 0.783 | 0.546 | 0.481 | 0.047 | 0.869 | 0.728 | 0.673 | 0.042 | 0.737 | 0.454 | 0.389 | 0.064 |
|  | **CeDAR-S** | 0.992 | 0.974 | 0.935 | 0.042 | 0.896 | 0.668 | 0.527 | 0.037 | 0.935 | 0.824 | 0.727 | 0.028 | 0.784 | 0.515 | 0.430 | 0.058 |
|  | **CeDAR-M** | 0.991 | 0.973 | 0.930 | 0.052 | 0.885 | 0.644 | 0.536 | 0.064 | 0.929 | 0.808 | 0.728 | 0.044 | 0.771 | 0.491 | 0.438 | 0.090 |
| **e** | **TOAST** | 0.975 | 0.941 | 0.894 | 0.047 | 0.783 | 0.535 | 0.461 | 0.044 | 0.870 | 0.712 | 0.648 | 0.039 | 0.727 | 0.421 | 0.352 | 0.058 |
|  | **TCA** | 0.977 | 0.946 | 0.904 | 0.048 | 0.788 | 0.557 | 0.492 | 0.045 | 0.874 | 0.734 | 0.677 | 0.050 | 0.732 | 0.447 | 0.381 | 0.043 |
|  | **csSAM** | 0.943 | 0.817 | 0.701 | 0.036 | 0.728 | 0.374 | 0.276 | 0.041 | 0.810 | 0.532 | 0.439 | 0.035 | 0.674 | 0.293 | 0.184 | 0.043 |
|  | **CellDMC** | 0.974 | 0.942 | 0.899 | 0.052 | 0.784 | 0.552 | 0.487 | 0.046 | 0.871 | 0.727 | 0.671 | 0.043 | 0.73 | 0.440 | 0.377 | 0.059 |
|  | **CeDAR-S** | 0.992 | 0.975 | 0.935 | 0.043 | 0.901 | 0.680 | 0.534 | 0.036 | 0.934 | 0.822 | 0.722 | 0.031 | 0.828 | 0.536 | 0.418 | 0.056 |
|  | **CeDAR-M** | 0.992 | 0.975 | 0.926 | 0.069 | 0.911 | 0.700 | 0.548 | 0.049 | 0.932 | 0.813 | 0.731 | 0.050 | 0.837 | 0.565 | 0.439 | 0.075 |
| **f** | **TOAST** | 0.974 | 0.940 | 0.893 | 0.048 | 0.781 | 0.530 | 0.457 | 0.046 | 0.861 | 0.699 | 0.636 | 0.039 | 0.723 | 0.414 | 0.346 | 0.067 |
|  | **TCA** | 0.976 | 0.946 | 0.904 | 0.047 | 0.785 | 0.553 | 0.489 | 0.046 | 0.864 | 0.719 | 0.665 | 0.047 | 0.728 | 0.440 | 0.376 | 0.045 |
|  | **csSAM** | 0.944 | 0.820 | 0.704 | 0.036 | 0.726 | 0.373 | 0.278 | 0.042 | 0.804 | 0.520 | 0.432 | 0.037 | 0.673 | 0.287 | 0.171 | 0.052 |
|  | **CellDMC** | 0.974 | 0.941 | 0.898 | 0.052 | 0.782 | 0.547 | 0.483 | 0.049 | 0.862 | 0.713 | 0.658 | 0.043 | 0.725 | 0.431 | 0.371 | 0.068 |
|  | **CeDAR-S** | 0.992 | 0.975 | 0.935 | 0.043 | 0.909 | 0.686 | 0.530 | 0.040 | 0.940 | 0.820 | 0.710 | 0.030 | 0.853 | 0.552 | 0.412 | 0.063 |
|  | **CeDAR-M** | 0.992 | 0.974 | 0.930 | 0.049 | 0.904 | 0.677 | 0.540 | 0.054 | 0.936 | 0.808 | 0.723 | 0.055 | 0.856 | 0.568 | 0.436 | 0.084 |

There are six different DE patterns corresponding to six panels in Figure S3 (a: all cell types are independent; b: all cell types are correlated under a single layer tree structure; c: only cell types 3 and 4 are correlated; d: only cell types 1 and 2 are correlated; e: cell types 1 and 2 are correlated, and cell types 3 and 4 are correlated; f: all cell types are correlated under a multiple-layer tree structure) The reported metrics (AUC-ROC, AUC-PR, MCC, and observed FDR) are average of 50 simulations. For TOAST, TCA, csSAM, and CellDMC the observed FDR was derived by calling DE with estimated FDR < 0.05; for CeDAR-S and CeDAR-M, the MCC and observed FDR was derived by calling DE with estimated posterior probability of DE > 0.95.

Table S4: Evaluation (AUC-ROC, AUC-PR, MCC and observed FDR) of CeDAR with true/estimated tree structure and true/estimated prior probability of nodes on the tree as input for cell type specific differential analyses.

| **Tree structure type** | **Prior probability type** | **Cell type 1** | | | | **Cell type 2** | | | | **Cell type 3** | | | | **Cell type 4** | | | | **Cell type 5** | | | | **Cell type 6** | | | | |
| --- | --- | --- | --- | --- | --- | --- | --- | --- | --- | --- | --- | --- | --- | --- | --- | --- | --- | --- | --- | --- | --- | --- | --- | --- | --- | --- |
|  |  | **ROC**  **AUC** | **PR**  **AUC** | **MCC** | **FDR** | **ROC**  **AUC** | **PR**  **AUC** | **MCC** | **FDR** | **ROC**  **AUC** | **PR**  **AUC** | **MCC** | **FDR** | **ROC**  **AUC** | **PR**  **AUC** | **MCC** | **FDR** | **ROC**  **AUC** | **PR**  **AUC** | **MCC** | **FDR** | **ROC**  **AUC** | **PR**  **AUC** | **MCC** | **FDR** |  |
| **True** | **True** | 0.982 | 0.940 | 0.872 | 0.069 | 0.933 | 0.720 | 0.512 | 0.165 | 0.849 | 0.592 | 0.509 | 0.186 | 0.833 | 0.513 | 0.406 | 0.250 | 0.808 | 0.441 | 0.347 | 0.280 | 0.795 | 0.426 | 0.341 | 0.243 |  |
| **True** | **Estimated** | 0.989 | 0.961 | 0.899 | 0.068 | 0.919 | 0.630 | 0.413 | 0.070 | 0.868 | 0.620 | 0.495 | 0.080 | 0.850 | 0.532 | 0.367 | 0.097 | 0.829 | 0.473 | 0.324 | 0.129 | 0.818 | 0.460 | 0.321 | 0.109 |  |
| **Estimated** | **Estimated** | 0.988 | 0.956 | 0.894 | 0.057 | 0.898 | 0.586 | 0.408 | 0.073 | 0.868 | 0.618 | 0.495 | 0.083 | 0.849 | 0.529 | 0.367 | 0.100 | 0.828 | 0.470 | 0.324 | 0.133 | 0.817 | 0.457 | 0.322 | 0.112 |  |

True tree structure/prior probability represents using parameters generating simulation data as CeDAR input. Estimated tree structure/prior probability represents using tree structure/prior probability that are estimated from estimation procedure described in Methods section. The reported metrics (AUC-ROC, AUC-PR, MCC, and observed FDR) are average of 50 simulations. For TOAST, TCA, csSAM, and CellDMC, the MCC and observed FDR was derived by calling DE with estimated FDR < 0.05; for CeDAR-S and CeDAR-M, the MCC and observed FDR was derived by calling DE with estimated posterior probability of DE > 0.95.

Table S5: Observed FDR of CeDAR-S with estimated/true prior probability as input on simulated data with different noise level (two cell types).

| **Noise level** | **Estimated prior probability** | | **True prior**  **probability** | |
| --- | --- | --- | --- | --- |
|  | **FDR in Cell type 1** | **FDR in Cell type 2** | **FDR in Cell type 1** | **FDR in Cell type 2** |
| **0.01** | 0.043 | 0.039 | 0.026 | 0.024 |
| **0.1** | 0.043 | 0.037 | 0.026 | 0.025 |
| **1** | 0.063 | 0.047 | 0.024 | 0.083 |
| **2** | 0.046 | 0.126 | 0.024 | 0.225 |

True prior probability represents using parameters generating simulation data as CeDAR input. Estimated tree prior probability represents using estimated prior probability that are estimated from estimation procedure described in Methods section. The reported observed FDR is average of 50 simulations. The observed FDR was derived by calling DE with estimated posterior probability of DE > 0.95. Noise level 1 is the parameter setting we used in other simulations. For other noise levels (extremely low 0.01, low: 0.1, high: 2), we multiply 0.01, 0.1 or 2 to the standard deviation of both cell type specific gene expression and bulk expression. Sample size is 100 per group.

Table S6: Observed FDR of CeDAR-M with estimated/true prior probability as input on simulated data with different noise level (six cell types)

| **Prior prob and tree structure type** | **Noise level** | **FDR in**  **Cell type 1** | **FDR in**  **Cell type 2** | **FDR in**  **Cell type 3** | **FDR in**  **Cell type 4** | **FDR in**  **Cell type 5** | **FDR in**  **Cell type 6** |
| --- | --- | --- | --- | --- | --- | --- | --- |
| **Estimated** | **0.01** | 0.097 | 0.089 | 0.105 | 0.108 | 0.096 | 0.073 |
|  | **0.1** | 0.095 | 0.080 | 0.101 | 0.095 | 0.089 | 0.069 |
|  | **1** | 0.057 | 0.073 | 0.083 | 0.100 | 0.133 | 0.112 |
|  | **2** | 0.042 | 0.206 | 0.168 | 0.292 | 0.395 | 0.374 |
| **True** | **0.01** | 0.068 | 0.066 | 0.086 | 0.090 | 0.076 | 0.056 |
|  | **0.1** | 0.068 | 0.069 | 0.089 | 0.095 | 0.082 | 0.062 |
|  | **1** | 0.069 | 0.165 | 0.186 | 0.250 | 0.280 | 0.243 |
|  | **2** | 0.107 | 0.345 | 0.408 | 0.528 | 0.587 | 0.555 |

True tree structure/prior probability represents using parameters generating simulation data as CeDAR input. Estimated tree structure/prior probability represents using prior probability that are estimated from estimation procedure described in Methods section. The reported observed FDR is average of 50 simulations. The observed FDR was derived by calling DE with estimated posterior probability of DE > 0.95. Noise level 1 is the parameter setting we used in other simulations. For other noise levels (extremely low 0.01, low: 0.1, high: 2), we multiply 0.01, 0.1 or 2 to the standard deviation of both cell type specific gene expression and bulk expression. Sample size is 100 per group.

Table S7: Evaluation (AUC-ROC, AUC-PR, MCC and observed FDR) of CeDAR-M with correct/mis-specified tree structure as input for cell type specific differential expression analyses from different methods.

| **Sample size** | **Methods** | **Cell type 1** | | | | **Cell type 2** | | | | **Cell type 3** | | | | **Cell type 4** | | | | **Cell type 5** | | | | **Cell type 6** | | | |
| --- | --- | --- | --- | --- | --- | --- | --- | --- | --- | --- | --- | --- | --- | --- | --- | --- | --- | --- | --- | --- | --- | --- | --- | --- | --- |
|  |  | **ROC**  **AUC** | **PR**  **AUC** | **MCC** | **FDR** | **ROC**  **AUC** | **PR**  **AUC** | **MCC** | **FDR** | **ROC**  **AUC** | **PR**  **AUC** | **MCC** | **FDR** | **ROC**  **AUC** | **PR**  **AUC** | **MCC** | **FDR** | **ROC**  **AUC** | **PR**  **AUC** | **MCC** | **FDR** | **ROC**  **AUC** | **PR**  **AUC** | **MCC** | **FDR** |
| **50** | **Tree 1**  **(Correct)** | 0.979 | 0.923 | 0.834 | 0.067 | 0.897 | 0.531 | 0.284 | 0.128 | 0.821 | 0.493 | 0.363 | 0.124 | 0.802 | 0.413 | 0.257 | 0.175 | 0.780 | 0.352 | 0.201 | 0.237 | 0.771 | 0.348 | 0.201 | 0.202 |
|  | **Tree 2**  **(Mis-specified)** | 0.977 | 0.911 | 0.822 | 0.074 | 0.852 | 0.437 | 0.285 | 0.175 | 0.816 | 0.468 | 0.330 | 0.105 | 0.783 | 0.364 | 0.238 | 0.199 | 0.771 | 0.332 | 0.195 | 0.247 | 0.766 | 0.339 | 0.198 | 0.208 |
|  | **Tree 3**  **(Mis-specified)** | 0.979 | 0.923 | 0.834 | 0.067 | 0.897 | 0.532 | 0.285 | 0.127 | 0.819 | 0.484 | 0.357 | 0.127 | 0.799 | 0.393 | 0.238 | 0.154 | 0.780 | 0.351 | 0.200 | 0.239 | 0.770 | 0.353 | 0.211 | 0.226 |
|  | **Tree 4**  **(Mis-specified)** | 0.979 | 0.923 | 0.833 | 0.067 | 0.897 | 0.531 | 0.285 | 0.127 | 0.820 | 0.489 | 0.360 | 0.124 | 0.801 | 0.401 | 0.246 | 0.166 | 0.781 | 0.359 | 0.209 | 0.247 | 0.771 | 0.348 | 0.201 | 0.200 |
|  | **Tree 5**  **(Mis-specified)** | 0.976 | 0.908 | 0.816 | 0.071 | 0.854 | 0.439 | 0.285 | 0.168 | 0.812 | 0.464 | 0.347 | 0.135 | 0.789 | 0.366 | 0.223 | 0.162 | 0.775 | 0.340 | 0.196 | 0.243 | 0.768 | 0.342 | 0.198 | 0.204 |
| **100** | **Tree 1**  **(Correct)** | 0.989 | 0.961 | 0.899 | 0.068 | 0.919 | 0.630 | 0.413 | 0.070 | 0.868 | 0.620 | 0.495 | 0.080 | 0.850 | 0.532 | 0.367 | 0.097 | 0.829 | 0.473 | 0.324 | 0.129 | 0.818 | 0.460 | 0.321 | 0.109 |
|  | **Tree 2**  **(Mis-specified)** | 0.987 | 0.952 | 0.888 | 0.073 | 0.871 | 0.532 | 0.412 | 0.102 | 0.862 | 0.587 | 0.453 | 0.063 | 0.828 | 0.472 | 0.345 | 0.111 | 0.819 | 0.449 | 0.316 | 0.134 | 0.813 | 0.449 | 0.317 | 0.109 |
|  | **Tree 3**  **(Mis-specified)** | 0.989 | 0.961 | 0.899 | 0.068 | 0.920 | 0.630 | 0.413 | 0.070 | 0.866 | 0.609 | 0.486 | 0.080 | 0.845 | 0.505 | 0.343 | 0.084 | 0.829 | 0.471 | 0.321 | 0.128 | 0.818 | 0.469 | 0.334 | 0.126 |
|  | **Tree 4**  **(Mis-specified)** | 0.989 | 0.961 | 0.899 | 0.068 | 0.919 | 0.630 | 0.413 | 0.070 | 0.867 | 0.614 | 0.490 | 0.081 | 0.847 | 0.516 | 0.353 | 0.092 | 0.830 | 0.481 | 0.332 | 0.138 | 0.818 | 0.460 | 0.321 | 0.109 |
|  | **Tree 5**  **(Mis-specified)** | 0.987 | 0.951 | 0.886 | 0.072 | 0.873 | 0.536 | 0.411 | 0.097 | 0.858 | 0.585 | 0.472 | 0.083 | 0.831 | 0.469 | 0.326 | 0.087 | 0.823 | 0.457 | 0.315 | 0.130 | 0.815 | 0.452 | 0.316 | 0.106 |
| **200** | **Tree 1**  **(Correct)** | 0.994 | 0.980 | 0.930 | 0.069 | 0.940 | 0.723 | 0.534 | 0.052 | 0.905 | 0.725 | 0.617 | 0.066 | 0.889 | 0.643 | 0.489 | 0.067 | 0.870 | 0.584 | 0.445 | 0.090 | 0.863 | 0.587 | 0.456 | 0.067 |
|  | **Tree 2**  **(Mis-specified)** | 0.992 | 0.974 | 0.925 | 0.068 | 0.894 | 0.630 | 0.527 | 0.075 | 0.899 | 0.694 | 0.576 | 0.047 | 0.867 | 0.583 | 0.465 | 0.074 | 0.859 | 0.560 | 0.437 | 0.089 | 0.859 | 0.577 | 0.451 | 0.066 |
|  | **Tree 3**  **(Mis-specified)** | 0.994 | 0.980 | 0.930 | 0.069 | 0.940 | 0.723 | 0.534 | 0.052 | 0.903 | 0.715 | 0.607 | 0.063 | 0.883 | 0.614 | 0.464 | 0.054 | 0.869 | 0.581 | 0.442 | 0.087 | 0.864 | 0.597 | 0.470 | 0.081 |
|  | **Tree 4**  **(Mis-specified)** | 0.994 | 0.980 | 0.930 | 0.069 | 0.940 | 0.723 | 0.534 | 0.052 | 0.904 | 0.719 | 0.612 | 0.065 | 0.886 | 0.626 | 0.474 | 0.061 | 0.871 | 0.593 | 0.455 | 0.096 | 0.863 | 0.587 | 0.456 | 0.067 |
|  | **Tree 5**  **(Mis-specified)** | 0.992 | 0.973 | 0.923 | 0.068 | 0.896 | 0.635 | 0.526 | 0.070 | 0.895 | 0.692 | 0.592 | 0.063 | 0.869 | 0.579 | 0.446 | 0.054 | 0.863 | 0.565 | 0.436 | 0.086 | 0.860 | 0.579 | 0.449 | 0.065 |

The simulation mimics a two-group comparison based on bulk microarray gene expression – a mixture of six common blood immune cell types (1: Neutrophils, 2: Monocytes, 3: CD4+, 4: CD8+ cells, 5: B cells, 6: NK cells) with different sample sizes per group (50, 100, and 200). “tree 1” is the correct tree structure used to generated simulation data; “tree 2”, “tree 3”, “tree 4” and “tree 5” are mis-specified tree structures by switching cell type 2 with cell type 3, and by switching cell type 4 with cell type 2/5/6, which were used for evaluating impact of mis-specified tree structure. The reported metrics (AUC-ROC, AUC-PR, MCC, and observed FDR) are average of 50 simulations. The MCC and observed FDR were derived by calling DE with estimated posterior probability of DE > 0.95.

Table S8: Evaluation (AUC-ROC, AUC-PR, MCC and observed FDR) of different methods with true/estimated cell type composition as input for cell type specific differential expression analyses from different methods.

| **Proportion type** | **Methods** | **Cell type 1** | | | | **Cell type 2** | | | | **Cell type 3** | | | | **Cell type 4** | | | | **Cell type 5** | | | | **Cell type 6** | | | |
| --- | --- | --- | --- | --- | --- | --- | --- | --- | --- | --- | --- | --- | --- | --- | --- | --- | --- | --- | --- | --- | --- | --- | --- | --- | --- |
|  |  | **ROC**  **AUC** | **PR**  **AUC** | **MCC** | **FDR** | **ROC**  **AUC** | **PR**  **AUC** | **MCC** | **FDR** | **ROC**  **AUC** | **PR**  **AUC** | **MCC** | **FDR** | **ROC**  **AUC** | **PR**  **AUC** | **MCC** | **FDR** | **ROC**  **AUC** | **PR**  **AUC** | **MCC** | **FDR** | **ROC**  **AUC** | **PR**  **AUC** | **MCC** | **FDR** |
| **True** | **TOAST** | 0.948 | 0.870 | 0.796 | 0.047 | 0.708 | 0.389 | 0.308 | 0.047 | 0.734 | 0.438 | 0.358 | 0.039 | 0.672 | 0.329 | 0.251 | 0.041 | 0.662 | 0.301 | 0.220 | 0.069 | 0.667 | 0.309 | 0.213 | 0.055 |
|  | **TCA** | 0.951 | 0.881 | 0.816 | 0.059 | 0.710 | 0.405 | 0.338 | 0.061 | 0.738 | 0.457 | 0.393 | 0.059 | 0.675 | 0.344 | 0.278 | 0.061 | 0.664 | 0.317 | 0.239 | 0.052 | 0.668 | 0.320 | 0.233 | 0.056 |
|  | **csSAM** | 0.902 | 0.697 | 0.535 | 0.038 | 0.665 | 0.276 | 0.176 | 0.036 | 0.681 | 0.310 | 0.228 | 0.039 | 0.630 | 0.240 | 0.160 | 0.035 | 0.621 | 0.217 | 0.095 | 0.040 | 0.624 | 0.217 | 0.112 | 0.040 |
|  | **CellDMC** | 0.948 | 0.877 | 0.812 | 0.052 | 0.708 | 0.404 | 0.332 | 0.051 | 0.737 | 0.456 | 0.383 | 0.043 | 0.675 | 0.343 | 0.270 | 0.046 | 0.663 | 0.315 | 0.239 | 0.071 | 0.669 | 0.324 | 0.234 | 0.058 |
|  | **CeDAR-S** | 0.988 | 0.959 | 0.901 | 0.044 | 0.898 | 0.583 | 0.395 | 0.047 | 0.865 | 0.597 | 0.450 | 0.046 | 0.836 | 0.483 | 0.325 | 0.056 | 0.822 | 0.449 | 0.296 | 0.089 | 0.815 | 0.452 | 0.298 | 0.079 |
|  | **CeDAR-M** | 0.988 | 0.958 | 0.896 | 0.057 | 0.902 | 0.596 | 0.411 | 0.069 | 0.867 | 0.618 | 0.497 | 0.083 | 0.848 | 0.531 | 0.371 | 0.099 | 0.828 | 0.473 | 0.324 | 0.127 | 0.815 | 0.458 | 0.319 | 0.114 |
| **Estimated** | **TOAST** | 0.940 | 0.859 | 0.793 | 0.089 | 0.708 | 0.342 | 0.187 | 0.147 | 0.647 | 0.259 | 0.170 | 0.304 | 0.650 | 0.281 | 0.198 | 0.102 | 0.615 | 0.216 | 0.108 | 0.162 | 0.613 | 0.208 | 0.087 | 0.216 |
|  | **TCA** | 0.943 | 0.868 | 0.803 | 0.104 | 0.704 | 0.336 | 0.222 | 0.203 | 0.643 | 0.249 | 0.182 | 0.443 | 0.651 | 0.287 | 0.219 | 0.136 | 0.616 | 0.223 | 0.122 | 0.167 | 0.612 | 0.210 | 0.094 | 0.212 |
|  | **csSAM** | 0.899 | 0.703 | 0.576 | 0.062 | 0.674 | 0.244 | 0.064 | 0.124 | 0.615 | 0.192 | 0.043 | 0.370 | 0.619 | 0.211 | 0.123 | 0.105 | 0.589 | 0.167 | 0.027 | 0.093 | 0.587 | 0.158 | 0.030 | 0.165 |
|  | **CellDMC** | 0.940 | 0.865 | 0.802 | 0.097 | 0.708 | 0.352 | 0.210 | 0.154 | 0.648 | 0.268 | 0.184 | 0.304 | 0.651 | 0.292 | 0.213 | 0.108 | 0.615 | 0.224 | 0.118 | 0.161 | 0.613 | 0.215 | 0.097 | 0.215 |
|  | **CeDAR-S** | 0.978 | 0.926 | 0.822 | 0.194 | 0.884 | 0.519 | 0.331 | 0.199 | 0.801 | 0.371 | 0.241 | 0.371 | 0.804 | 0.413 | 0.272 | 0.168 | 0.782 | 0.342 | 0.191 | 0.208 | 0.772 | 0.321 | 0.168 | 0.306 |
|  | **CeDAR-M** | 0.978 | 0.926 | 0.815 | 0.211 | 0.897 | 0.548 | 0.355 | 0.214 | 0.807 | 0.401 | 0.287 | 0.382 | 0.802 | 0.425 | 0.306 | 0.223 | 0.781 | 0.353 | 0.211 | 0.248 | 0.770 | 0.321 | 0.179 | 0.336 |

“True” proportion represents using cell type compositions generating simulation data as input; “Estimated” proportion represents using estimated cell type compositions (by *ged()* function of *CellDMC* package) as input. The reported metrics (AUC-ROC, AUC-PR, MCC, and observed FDR) are average of 50 simulations. For TOAST, TCA, csSAM, and CellDMC, the MCC and observed FDR were derived by calling DE with estimated FDR < 0.05; for CeDAR-S and CeDAR-M, the MCC and observed FDR were derived by calling DE with estimated posterior probability of DE > 0.95.

Table S9: Computation time of various methods with different number of cell types and different sample sizes.

| **Methods** | **Cell type number** | **Sample size: 50** | **Sample size: 100** | **Sample size: 200** |
| --- | --- | --- | --- | --- |
| **TCA** | 4 | 531.495 | 603.709 | 751.259 |
| **csSAM** | 4 | 38.241 | 64.792 | 111.977 |
| **CellDMC** | 4 | 19.674 | 20.257 | 22.137 |
| **TOAST** | 4 | 0.161 | 0.395 | 1.353 |
| **CeDAR-M** | 4 | 3.611 | 10.369 | 33.879 |
| **TCA** | 6 | 618.769 | 679.791 | 876.800 |
| **csSAM** | 6 | 39.104 | 66.050 | 122.227 |
| **CellDMC** | 6 | 23.207 | 24.466 | 26.644 |
| **TOAST** | 6 | 0.154 | 0.409 | 1.383 |
| **CeDAR-M** | 6 | 10.877 | 36.759 | 130.927 |
| **TCA** | 8 | 870.97 | 757.761 | 989.960 |
| **csSAM** | 8 | 41.432 | 73.086 | 124.592 |
| **CellDMC** | 8 | 26.804 | 28.785 | 31.872 |
| **TOAST** | 8 | 0.176 | 0.424 | 1.405 |
| **CeDAR-M** | 8 | 50.959 | 152.417 | 524.238 |

TOAST, TCA, csSAM, CellDMC and CeDAR-M were evaluated for 12,402 genes with different number of cell types (4, 6, 8) and different sample sizes per group (50, 100, 200). Simulation was run on Linux with 2.80 GHz CPU and 8G RAM. Reported time (in seconds) is average of five simulations.

Table S10: Summary of simulated datasets used for evaluation CeDAR performance.

| **Corresponding results** | **Data type** | **Experiment design** | **cell type numbers** | **Sample size per group** | **DE state correlation** | **Input: Proportion** | **Input: Tree** | **Input: Prior prob** |
| --- | --- | --- | --- | --- | --- | --- | --- | --- |
| Figure 3, Table S1 | Gene  expression | Two group comparison | 6 | 50, 100, 200 | Correlated | True | Estimated | Estimated |
| Figure 4, S2 – S4, Table S2, S3 | Gene  expression | Two group comparison | 4 | 200 | Correlated, Independent, Partial correlated | True | Estimated | Estimated |
| Figure S5, Table S4 | Gene  expression | Two group comparison | 6 | 100 | Correlated | True | True, estimated | True, Estimated |
| Table S5, Table S6 | Gene  expression | Two group comparison | 2, 6 | 100 | Correlated | True | True, Estimated | True, Estimated |
| Figure S6, S7,  Table S7 | Gene  expression | Two group comparison | 4 | 50, 100, 200 | Correlated | True | True, Mis-specified | Estimated |
| Figure S8, S9, Table S8 | Gene  expression | Two group comparison | 6 | 50, 100, 200 | Correlated | True | True, Mis-specified | Estimated |
| Figure S10, Table S9 | Gene expression | Two group comparison | 6 | 100 | Correlated | True, Estimated | Estimated | Estimated |
| Table S10 | Gene  expression | Two group comparison | 4, 6, 8 | 50, 100, 200 | Correlated | True | Estimated | Estimated |

For the input proportion, tree, and prior prob columns, “true” means using parameters generating simulation data as input; “estimated” means using estimates following estimation procedure described in Methods section as input; “mis-specified” means arbitrarily defined incorrect input.

Table S11: Summary of real datasets used to demonstrate DE/DM state correlation between cell types

| **Dataset Name** | **Data platform** | **Data type** | **Factor to test** | **Cell types** |
| --- | --- | --- | --- | --- |
| GSE60424 | Illumina HiScanSQ | RNAseq  Counts | Treatment (before vs. after first treatment with IFN-beta of sclerosis patients) | Neutrophils, Monocytes, CD8 T-cells, CD4 T-cells, B-cells, NK cells |
| GSE149050 | Illumina HiSeq 2500 | RNAseq  Counts | Disease (SLE patients with highly expressed type I interferon-related genes vs. Healthy control) | T cells, B cells, Polymorphonuclear Neutrophils, conventional dendritic cells, plasmacytoid dendritic cells, classical monocytes |
| GSE59250 | Illumina HumanMethylation450 | DNA methylation Beta value | Disease (SLE vs. Healthy control) | CD4 T-cells, CD19 B-cells, CD14 Monocytes |
| GSE131525 | Illumina HiSeq 2500 | RNAseq  Counts | Disease (SLE vs. Healthy control) | CD4 T-cells, CD8 T-cells, B cells |
| GSE166844 | Infinium MethylationEPIC | DNA methylation Beta value | Sex (female vs. male) | Granulocytes, Monocytes, CD8 T-cells, CD4 T-cells, B-cells |

Table S12: Summary of real datasets used to evaluate CeDAR performance

| **Dataset Name** | **Data platform** | **Data type** | **Factor to test** | **Covariate to adjust** | **Validation method** | **Cell types** |
| --- | --- | --- | --- | --- | --- | --- |
| Human brain (GSE41826) | Illumina HumanMethylation450 | DNA methylation Beta value | Sex (5 female vs. 5 male) | NA | Pure cell type sample as gold standard | Glia, Neuron |
| Human whole blood (GSE166844) | Infinium MethylationEPIC | DNA methylation Beta value | Sex (18 female vs. 12 male) | NA | Pure cell type sample as gold standard | Granulocytes, Monocytes, CD8 T-cells, CD4 T-cells, B-cells |
| Human SLE (GSE118144) | Illumina HumanMethylation450 | DNA methylation Beta value | Disease (16 SLE vs. 13 Ctrl) | NA | Pure cell type sample as gold standard | Neutrophils, CD8 T-cells, CD4 T-cells, B-cells |
| Human DS (GSE74486) | Illumina HumanMethylation450 | DNA methylation Beta value | Disease (14 DS vs. 8 Ctrl) | NA | Pure cell type sample as gold standard | Glia, Neuron |
| RA EWAS Liu (GSE42861) | Illumina HumanMethylation450 | DNA methylation Beta value | Disease (354 RA vs. 332 Normal);  Smoke (686) | Sex, Age | Probes reported by Julia et al(32); Probes reported by Su et. al(31) | NA |
| Smoking EWAS Hannum (GSE40279) | Illumina HumanMethylation450 | DNA methylation Beta value | Smoke (656) | Plate, Age | Probes reported by Su et. al(31) | NA |

Table S13: Summary of tools used for evaluation of CeDAR performance in simulated and real data analysis

| **R package** | **Version** | **Purpose** | **Analysis type** | **Function used** |
| --- | --- | --- | --- | --- |
| TOAST | 1.9.5 | Cell type specific differential analysis | Simulation; Real data | cedar; csTest |
| TCA | 1.2.1 | Cell type specific differential analysis | Simulation; Real data | tca |
| CellMix | 1.6.2 | Cell type specific differential analysis; Cell type composition estimation | Simulation; Real data | CellDMC; ged |
| csSAM | 1.4 | Cell type specific differential analysis | Simulation; Real data | csSAMfit; csTopTable |
| ROCR | 1.0-11 | ROC curve and Precision - Recall curve generation; AUC-ROC and AUC-PR calculation | Simulation | prediction; performance |
| mltools | 0.3.5 | Matthews correlation coefficient calculation | Simulation | mcc |
| EpiDISH | 2.10.0 | Cell type composition estimation | Real data | epidish |
| minfi | 1.40.0 | Differential methylation analysis | Real data | dmpFinder |
| missMethyl | 1.28.0 | Enrichment analysis for methylation probes | Real data | gometh; topGSA |
| impute | 1.68.0 | Impute missing beta value for DNA methylation data | Real data | impute.knn |
