# Supplementary material for: CeDAR: incorporating cell type hierarchy improves cell type-specific differential analyses in bulk omics data
Source: Genome Biol. 2023 Feb 28;24:37. doi: 10.1186/s13059-023-02857-5 (PMC9972684; doi:10.1186/s13059-023-02857-5)
Supplement: Supplementary file 2 — Additional file 2: Review history. [file 13059_2023_2857_MOESM2_ESM.docx]

Review History

**First round of review**

**Reviewer 1**

**Were you able to assess all statistics in the manuscript, including the appropriateness of statistical tests used?** No

**Were you able to directly test the methods?** No

**Comments to author:**

Chen et al. developed a tool named CeDAR to incorporate the cell type hierarchy in cell-type-specific differential analyses in bulk data. My specific comments are as below:

1. Could the authors test more datasets to further demonstrate that CeDAR is superior to other methods?

2. A supplementary table regarding the details of simulated and real datasets used in this study may help readers better understand the results.

3. The authors mainly compared the performance of CeDAR with a few other tools, could the authors benchmark more relevant tools to show that CeDAR outperforms existing tools?

4. To help readers better understand the efficiency of CeDAR, could the authors compare the runtime of CeDAR with other tools?

5. Could the authors provide the AUC values of each subfigure in Figure 4? This may make Figure 4 more informative.

6. Figure 1 is of low quality, please revise it to a higher resolution.

7. Please provide the versions and the parameters of those tools used to test/compare their performance on both simulated and real datasets in this study.

8. More explanations about the data used to plot Figures 5 and 6 could be provided in the figure legends.

**Reviewer 2**

**Were you able to assess all statistics in the manuscript, including the appropriateness of statistical tests used?** No

**Were you able to directly test the methods?** No

**Comments to author:**

Title: CeDAR: incorporating cell type hierarchy improves cell type specific differential analysis in bulk omics data

Author: Luxiao Chen, Ziyi Li, Hao Wu

Decision: Minor revision

Summary

Traditional bulk data analysis for differential expression (DE) and differential mehtylation (DM) compare average signals among different groups. However, bulk cells consist of several cell types with different proportions. Among the several cell types, a gene or methylation loci may be differential in only some of the several cell types. Inferring this is an important research problem. Existing cell-type specific analysis from the bulk data performs well but their accuracy and power could be low especially in cell types with small proportion. Since it is known that differential cell types in a tissue form a hierarchical structure, similar cell types could exhibit similar DE / DM patterns and the Figure 1 shows that the data correlation comply with the assumption. Based on this observation, they developed a statistical method based on hierarchical Bayesian model named CeDAR to incorporate the cell type hierarchy in cell-type-specific differential analyses in bulk data. Extensive simulation and real data analyses demonstrate that this approach significantly improves the accuracy and power in detecting cell-type-specific differential signals compared with existing methods, especially in low abundance cell types.

Comment

* Suppose that we want to discover cell type specific DM, csDMCs, comparing control vs case. Assumed that cell type and cell type proportion as CeDAR given, recovering cell type specific methylation profile of case and control respectively and conduct differential analysis may be done relatively easy. Authors should define technical difficulties precisely, clarifying why incorporating factor of interest in a likelihood computation (equation 1) is reasonable and effective.

* Cell type hierarchical tree is constructed with hierarchical clustering, using distance metric defined by the authors; ½[1-corr(-log10(pvalk), - log10(pvalk)]. However, unlike similarity metric, distance metric should satisfy positivity, symmetry, identity-discerning, and triangular inequality. The authors should verify that the metric authors suggested satisfies such properties.

* According to the prior probability distribution proposed by the authors, if low proportional cell type shares a common parent with the high proportional cell type, the prior probability of low proportional cell type is deduced from the prior probability of high proportional cell type. Borrowing information across cell types is shown in Figure 3, but incorporating this concept in computable form needs more justification and clarity. For example, the authors should validate with equations, compared the joint probability P(Zg1=1,Zg2=1) vs P(Zg2=1,Zg3=1)

* In Figure 1, the authors compared the methylation and expression correlation between cell types and showed similar cell types (e.g. CD4 and CD8, which share common ancestor T cell) showed high correlation compared to the other dissimilar cell types these patterns are clear in methylation data, but much less clear in gene expression data. Since methylation event is binary event in CpG site and modeled as binary variable in the model, cell type specific patterns are more distinct among cell types and it is more credible to construct cell type hierarchical tree from methylation data while it is doubtful to use gene expression data which is continuous feature. Since all the real data are methylation profile, authors should verify or clarify whether the proposed model is applicable to expression data as well.

* In Figure 3, CeDAR-M and CeDAR showed high FDR compared to the other tools, which implies that other tools perform better in controlling type 1 error. Authors should clarify the evaluation metric and clarify the expression "However, compared to TOAST and TCA, the statistical inference of CeDAR-M and CeDAR-S are slightly more liberal in cell types with smaller proportions. (line: 198)"

* Analysis on real dataset 3 (Figure 7.) ceDAR reports much more csDMCs compared to TOAST and TCA and a large proportion of csDMCs are shared TOAST and TCA. KEGG pathway enrichment showed that these csDMCs are related to the pathways relevant to the phenotype of interest. However, since ceDAR reports too many positive csDMCs it may contain false positive csDMCs so the authors should validate the csDMCs.

* In Figure 1, the authors compared differential methylation pattern of female vs male and compared differential expression pattern of sclerosis patient before vs after IFN-g. It seems fair to compare the methylation and expression pattern in same condition.

* In Figure 2, if the figure aims to show how marginal probability is computed based on hierarchical cell type tree, the feature for the right most black circle in cell type 1,2,3 should not be marked as 1 since the marginal probability of the internal nodes is defined as a proportion of union of DEs across child cell types.

**Reviewer 3**

**Were you able to assess all statistics in the manuscript, including the appropriateness of statistical tests used?** No

**Were you able to directly test the methods?** No

**Comments to author:**

SUMMARY

The authors present CeDAR, a method for cell-type specific differential methylation/expression analysis in the context of bulk data. CeDAR differs from other methods in that it explicitly takes a cell(type) lineage tree into account and can share information across tree nodes. The authors apply CeDAR to simulated and real data and find improved performance compared to current approaches.

GENERAL COMMENTS

The manuscript is generally clear and well written, and leveraging cell(type) lineage tree information to improve differential methylation and expression analyses is compelling in general. The authors motivate their method well and perform a detailed simulation study. Further on, noticeably more precise cell-type specific inferences from bulk data would have strong potential for impact; however, there are several issues with the current manuscript.

1) No accessible software tool. The authors present a method and the major result reported is improved performance compared with current approaches. However, the manuscript does not mention a software tool, nor is one provided. If a readily usable tool is not provided, it is unclear what the community's interest in the manuscript should be.

2) Reproducibility. While the authors describe their cedar method well, details about the real data analysis are missing, making reproduction of reported results difficult at best.

3) Simulation study. The approach for simulating data seems to recapitulate CeDAR's assumptions. This would be good for a proof of concept but bias the results in CeDAR's favor. Reporting CeDAR's performance in simulations matched to the other methods would be fair.

4) Real data study. While the TDR curves are compelling, it is unclear what is learnt from the additionally recovered sites. Performance of CeDAR and TOAS on validated csDMCs seesm very similar (Figure 7); form the pathway analysis using uniquely CeDAR csDMCs it is not clear if csDMCs of say TOAST would not highlight similar pathways, just via different sites. Showing a more direct impact on biological interpretation when using CeDAR vs other methods would be helpful.

SPECIFIC COMMENTS

* The authors use two real datasets to demonstrate strong correlations of differential methylation/expression between cell types, which, in principle, is not a lot of support for assuming this holds true in general. Broader support and/or a short discussion why that makes sense in the context differentiation and development would be helpful.

* Figure 1 is convincing for the differential methylation part, but much less so for differential expression. This should be reflected in the corresponding discussion in the main text. On P 5 L 96 the authors write that results for expression are similar to those for methylation data, which does not reflect the figure 1.

* The manuscript is a bit confusing when talking about DE/DM state on P 6 L 125/126, and P 17 L 434 (i.e., that a celltype can only be DE/DM if its parent is), in combination with the statement that "interaction terms are only included for celltypes deemed DE/DM". This implies that no DE/DM can emerge during differentiation, which does not make sense biologically. Rather, what seems to be the case is that the DE/DM state encodes the potential for DE/DM, where parent celltypes must have low effect size to enable their children's noticeable DE/DM. In any case, it would be good to make this point more clearly.

* In the simulation setup, it would be good to explore how the method performs when the tree is mis-specified (or mis-inferred). That is data is simulated with one tree and inferred with a different one.

* Since the simulation setup appears very well matched to the CeDAR's assumptions, results are likely biased in CeDARs favor. It would be fair to simulate data according to TOAST and TCA and show results as well, at least for a subset of analyses.

* For performance assessment, in addition to ROC curves a performance measure more geared towards imbalanced data (like average precision (i.e., area under the precision recall curve)) or mcc should be reported.

* It is interesting that the FDR is inflated for CeDAR with small sample size in the simulation study (P8 L 200), but that this is alleviated by using estimated trees and priors. This seems counter-intuitive, it would be good to comment.

* In the section about computation performance, it would be good to be explicit on how CeDAR scales with the number of samples (linear?) and with the number of celltypes (exponential?).

* P 25 L 593 (and others). Language like "minfi was used to pre-process raw data and call gold standard csDMC" is not sufficient. Either each step that is executed has to be described in detail and parameters need to be provided, or the authors can make their code available.

**Authors Response**

We thank the editor and reviewers for their thorough review, thoughtful comments, and constructive suggestions for our manuscript “CeDAR: incorporating cell type hierarchy improves cell type specific differential analyses in bulk omics data” (GBIO-D-22-00185).

Based on the editor and reviewers’ comments, we have carefully revised the manuscript (modifications in the manuscript are highlighted in red font). In this revision, we made following major modifications:

1.	Obtain three new real datasets and conduct cell type specific differential methylation analysis to evaluate performance of CeDAR.

2.	Conduct additional simulation study to evaluate the impact of mis-specified tree structure on the cell type specific differential analysis results.

3.	Add two additional existing methods in the comparison with CeDAR (csSAM and CellDMC).

4.	Apply two metrics, area under precision and recall curve (AUC-PR) and Matthews Correlation Coefficient (MCC), in simulation results to evaluate the performance.

5.	Provide more real data results to illustrate the existence of DM/DM state correlation among cell types, including peer-reviewed research results and real data analysis on two gene expression data and one DNA methylation data.

6.	Benchmark the computational performance of CeDAR with other methods.

7.	Provide R scripts online to facilitate reproducing major results in manuscript; Provide more detailed descriptions of real data analysis process in manuscript and supplementary file.

We addressed the individual points raised by the reviewers, as detailed below. The reviewers’ comments are in italic blue, and our replies are in regular font.

**Point-by-point responses to the reviewers’ comments:**

*Chen et al. developed a tool named CeDAR to incorporate the cell type hierarchy in cell-type-specific differential analyses in bulk data. My specific comments are as below:*

*(****Comment 1****) Could the authors test more datasets to further demonstrate that CeDAR is superior to other methods?*

Thank you for the suggestion. As requested, we identified three additional real datasets and performed the following analyses:

-	Detecting Down syndrome (DS) associated cell type specific differential methylation (csDM) sites from frontal cortex grey matter samples in DNA methylation data (GSE74886^1^).

-	Detecting Systemic lupus erythematosus (SLE) associated csDM sites from whole blood samples in DNA methylation data (GSE118114^2^).

-	Detecting smoking associated csDM sites from whole blood samples in two DNA methylation datasets (GSE42861^3^ and GSE40279^4^) separately.

For the former two real datasets (GSE74486 and GSE118144), the data include both bulk and pure cell type sequencing samples. The pure cell type profiles can be used to provide gold standard for validating the identified csDM sites from the bulk data. Accuracy was evaluated by true discovery rate (TDR) among top ranked sites. The latter two datasets (GSE42861 and GSE40279) do not have purified cellular profiles. Instead, we checked whether they could identify seven cell type specific smoking associated probes that were reported previously by Su et al.^5^. The results are in the Additional file 1: Figure S10 for the DS data, Figure S11 for the SLE data, and Figure S12 for the smoking analysis.

In the analysis of detecting DS associated csDM sites (GSE74886), CeDAR-S achieves the highest accuracy in detecting both glia-specific DM sites and neuron-specific DM sites. From Figure S10 in Additional file 1, we can observe that the difference of TDR between CeDAR-S and other methods (TOAST, TCA, csSAM, and CellDMC) among top ranked 5,000 sites is more than 20% in either glia or neuron cell type.

Similar conclusion can be derived from analysis of SLE DNA methylation data (GSE118144). CeDAR-S and CeDAR-M have higher accuracy than all other methods (TOAST, TCA, csSAM, and CellDMC). From Figure S11 in Additional file 1, we see that in large proportion cell type neutrophils (Neutro), CeDAR-S and CeDAR-M have much higher TDR curve than other methods; in small proportion cell types such as CD4T, CD8T and B cells, the performances of all methods are not good, but only CeDAR-S and CeDAR-M can identify some true DM sites among top ranked 5,000 sites.

In the analysis of detecting smoking associated csDM sites (GSE42861 and GSE40279), CeDAR-S can identify more smoking associated csDM sites than TOAST, TCA, csSAM and CellDMC. From Figure S12 in Additional file 1, we observe that while TOAST, TCA, csSAM and CellDMC identify four myeloid-specific sites but zero lymphoid-specific sites, CeDAR-S can identify all myeloid-specific sites and one more lymphoid-specific site in Liu’s data (GSE42861). In Hannum’s data (GSE40279), CeDAR-S can identify four out of five myeloid-specific sites and one lymphoid-specific site, while the other methods can at most identify only one myeloid-specific site.

Collectively, these additional analyses demonstrate that our proposed method can achieve more accurate and interpretable results than other existing methods.

The details of the analysis procedure and results are included in the Additional file 1 Section S8. We also provide some discussions on these results in the main text Section Real data analysis – Other real data results.

*(****Comment 2****) A supplementary table regarding the details of simulated and real datasets used in this study may help readers better understand the results.*

Thank you for this helpful suggestion. We have provided the details of simulated and real datasets in table as following (also available in Additional file 1 Table S10, S11, S12). We also provided this information in the section of **Availability of data and materials** in the main manuscript.

Summary of simulated datasets used for evaluation CeDAR performance.

Summary of real datasets used to demonstrate DE/DM state correlation between cell types

Summary of real datasets used to evaluate CeDAR performance

*(****Comment 3****) The authors mainly compared the performance of CeDAR with a few other tools, could the authors benchmark more relevant tools to show that CeDAR outperforms existing tools?*

Thank you for this comment. We have now included two more existing methods csSAM^7^ and CellDMC^8^ into both simulation and real data analysis. csSAM is a two-stage model that separates the estimation of cell-type-specific mean profile and the statistical testing of identifying cell type specific markers. CellDMC uses a similar linear regression framework as TOAST^9^ but its primary data of interest is DNA methylation.

We have updated Figure 3 – 7 in manuscript, Figure S2 – S4, S8, S10 – S12, and Table S1 – S3, S8 in Additional file 1 to include results from both methods. As expected, CellDMC provides similar results as TOAST, since they are based on a similar linear model. The results from csSAM are the worst, which is consistent with previously published results^9^. Overall, these results demonstrate that CeDAR has better performance than existing methods in calling DM sites or DE genes. We have updated the Results section to provide related descriptions and discussions.

*(****Comment 4****) To help readers better understand the efficiency of CeDAR, could the authors compare the runtime of CeDAR with other tools?*

Thank you for the suggestion. We summarized the runtime of all methods under a simulation scenario with 12,402 genes, but varying cell type numbers (4,6, and 8) and sample sizes (50, 100, and 200). The results shown in the table below is the average run time (in seconds) of five simulations. All simulations are run on a PC with Linux system with 2.80 GHz CPU and 8G RAM.

In all scenarios, TOAST is the fastest. For example, it takes 1.405 seconds for 8 cell types and 200 samples. CellDMC is the second fastest on average. csSAM is a permutation-based method so that its runtime depends on the number of permutations. In this simulation, we used the default permutation number of 200. When there are four cell types with sample sizes 50, 100, 200 or six cell types with sample sizes 50, 100, csSAM takes longer time than CeDAR-M. Among all methods, TCA is the slowest. Overall, these results show that CeDAR provides a reasonable computational efficiency. With 200 samples and 8 cell types, CeDAR-M can complete the computation within 10 minutes, and when the cell type number decreases to 6, CeDAR-M only takes 2 minutes.

We have added additional description to the main manuscript (Section Simulation results – Computation performance) and the table to Additional file 1 (Table S9).

*(****Comment 5****) Could the authors provide the AUC values of each subfigure in Figure 4? This may make Figure 4 more informative.*

We appreciate the suggestion. We have included the AUC values of each subfigure in Figure 4 in the additional file. All summary statistics of simulation analyses are put together in Additional file 1 (Table S1 – S8). The following is the AUC of ROCR for Figure 4:

*(****Comment 6****) Figure 1 is of low quality, please revise it to a higher resolution.*

Thank you for this comment. The low quality of Figure 1 may be a compilation problem on the Genome Biology submission system. We uploaded the high-resolution figure separately in the submission (Figure 1.pdf). If the compiled file still shows low quality figure, please download the high-resolution one from the link provided on the top right of the Figure 1 page in manuscript.

*(****Comment 7****) Please provide the versions and the parameters of those tools used to test/compare their performance on both simulated and real datasets in this study.*

Thank you for this suggestion. We have listed the version of all tools in the following table (also available in Additional file 1 Table S13). The parameters used in these functions are stated in the description of simulation and real data analysis in Methods section. In addition, for reproducibility purpose, all scripts used to generate simulation and real data analysis are available in our GitHub repository (<https://github.com/luxiao10/CeDAR_reproduction>).

Summary of tools used for performance evaluation in simulated and real data analysis

*(****Comment 8****) More explanations about the data used to plot Figures 5 and 6 could be provided in the figure legends.*

Thank you for this helpful suggestion. We have added more explanation about the data used to plot Figures 5 and 6 in the figure legends as following:

*“Figure 5: Accuracy of detecting csDM in human brain methylation data. The human brain DNA methylation dataset (GEO accession number: GSE41826) contains both bulk samples from postmortem frontal cortex and matched cell type samples of Neuron and Glia purified by fluorescence activated cell sorting (FACS). The csDM sites associated with sex were identified between five healthy male and five healthy female samples with TOAST, TCA, csSAM, CellDMC and CeDAR-S. The results are evaluated by the true discovery rate (TDR) curves, which show the accuracy among different numbers of top-ranked csDM sites from each method.”*

*“Figure 6: Accuracy of detecting csDM in human blood methylation data. The human blood DNA methylation dataset (GEO accession number: GSE166844) contains both bulk samples from whole blood and pure cell type samples of granulocytes, CD8, CD4, monocytes and B cells derived by FACS. The csDM sites associated with sex were identified between eighteen females and twelve males samples using TOAST, TCA, csSAM, CellDMC, CeDAR-S, and CeDAR-M. The results are evaluated by TDR curves. The estimated proportions and estimated tree structure of cell types are shown in the last panel.”*

**Response to Reviewer 2’s comments:**

*Summary*

*Traditional bulk data analysis for differential expression (DE) and differential methylation (DM) compare average signals among different groups. However, bulk cells consist of several cell types with different proportions. Among the several cell types, a gene or methylation loci may be differential in only some of the several cell types. Inferring this is an important research problem. Existing cell-type specific analysis from the bulk data performs well but their accuracy and power could be low especially in cell types with small proportion. Since it is known that differential cell types in a tissue form a hierarchical structure, similar cell types could exhibit similar DE / DM patterns and the Figure 1 shows that the data correlation comply with the assumption. Based on this observation, they developed a statistical method based on hierarchical Bayesian model named CeDAR to incorporate the cell type hierarchy in cell-type-specific differential analyses in bulk data.*

*Extensive simulation and real data analyses demonstrate that this approach significantly improves the accuracy and power in detecting cell-type-specific differential signals compared with existing methods, especially in low abundance cell types.*

We appreciate the nice summary.

*(****Comment 1****) Suppose that we want to discover cell type specific DM, csDMCs, comparing control vs case. Assumed that cell type and cell type proportion as CeDAR given, recovering cell type specific methylation profile of case and control respectively and conduct differential analysis may be done relatively easy. Authors should define technical difficulties precisely, clarifying why incorporating factor of interest in a likelihood computation (equation 1) is reasonable and effective.*

Thank you for the comment. Given cell types and corresponding proportions, it is natural to think of a two-step approach like you described: estimating cell type specific methylation profile of different groups first and then perform statistical test on the estimated quantities. The csSAM method (published in 2010) was exactly designed in this way. However, such a two-step approach often suffers low accuracy, since the second step takes the estimation from the first step as observed data without considering their estimation variabilities. Li et al.^9^ provided a comprehensive comparison between csSAM and TOAST and reported that the two-step method has much lower accuracy. We have now included csSAM in the comparison. The new results (Figure 3 – 7 in main text; Figure S2 – S4, S8, S10 – S12; Table S1 – S3, S8 in additional file 1) show that it has worse performance compared with the other methods.

*(****Comment 2****) Cell type hierarchical tree is constructed with hierarchical clustering, using distance metric defined by the authors; ½[1-corr(-log10(pvalk), - log10(pvalk)]. However, unlike similarity metric, distance metric should satisfy positivity, symmetry, identity-discerning, and triangular inequality. The authors should verify that the metric authors suggested satisfies such properties.*

Thank you for this helpful comment. The metric we suggested is indeed a similarity metric instead of a distance metric. We have corrected our description in the manuscript consistently to reflect the correct notion. In addition, we have made *cedar()* function in TOAST package to accept any other user-defined similarity or distance metrics for estimating tree structures, which allows a more flexible application of CeDAR. The updated function can be accessed on Bioconductor (<https://www.bioconductor.org/packages/devel/bioc/html/TOAST.html>).

*(****Comment 3****) According to the prior probability distribution proposed by the authors, if low proportional cell type shares a common parent with the high proportional cell type, the prior probability of low proportional cell type is deduced from the prior probability of high proportional cell type. Borrowing information across cell types is shown in Figure 3 but incorporating this concept in computable form needs more justification and clarity. For example, the authors should validate with equations, compared the joint probability P (Zg1=1, Zg2=1) vs P (Zg2=1, Zg3=1)*

Thank you for this comment. We first want to point out that “*the prior probability of low proportional cell type is deduced from the prior probability of high proportional cell type”* is not entirely accurate. The correlations among cell types are accounted for through the cell type hierarchy. We now provide an intuitive example in the “Method overview” subsection of the Result section: “*Assuming there are three cell types forming a simple tree with one root node, one internal node, and three leaf nodes. ... If a gene shows strong DE in cell type 2, it will increase the probability for its parent node (an internal node) to be DE, which subsequently increases the prior probability for this gene to be also DE in cell type 3. Thus, the correlation between cell types 2 and 3 is passed through their parent node*”. We also provide some detailed, concrete examples of different joint and conditional probabilities, in the “Prior probabilities for the DE/DM states” subsection of the Method section. Below we reiterate some key points.

Under the CeDAR model assumption, a gene has a prior probability to be “potential DE” in target cell types (is potential DE). Then, if the gene is a “potential DE gene” in cell type , gene has a probability to be DE in one cell type ( is DE in cell type ); otherwise, gene cannot be DE in the cell type . If we explain this using a simple example, suppose we have two cell types (1 and 2), we assume the two cell types show a certain level correlation in DE state. Then by setting a parent node for the two cell types we have:

If we assume the DE state of the two cell types are independent (Implicit assumption for TOAST/CellDMC), then the joint probability for the two cell types showing DE for a gene is

In general, the two equations have different values. So, by applying the conditional probability, if two cell types show DE state correlation, they have a higher joint probability to be DE simultaneously. Such idea has been successfully applied in other works like JAMIE^10^.

In addition, cell types have closer distance on the tree (more similar with each other) should have higher joint probability to be DE simultaneously. Using the toy example shown in Figure 2, cell type 2 and 3 are more similar than cell type 2 and cell type 1. Even though cell type 3 and cell type 1 have the same marginal probability for a random gene to be DE (), their joint probabilities to be DE with cell type 2 are different:

*(Comment 4) In Figure 1, the authors compared the methylation and expression correlation between cell types and showed similar cell types (e.g., CD4 and CD8, which share common ancestor T cell) showed high correlation compared to the other dissimilar cell types these patterns are clear in methylation data, but much less clear in gene expression data. Since methylation event is binary event in CpG site and modeled as binary variable in the model, cell type specific patterns are more distinct among cell types and it is more credible to construct cell type hierarchical tree from methylation data while it is doubtful to use gene expression data which is continuous feature. Since all the real data are methylation profile, authors should verify or clarify whether the proposed model is applicable to expression data as well.*

Thank you for this comment. First of all, Figure 1 shows the correlation of DNA methylation/gene expression differences between two groups for pairs of cell types (represented by the statistical p-value), not the correlation of methylation/expression values themselves. The point in Figure 1 represents transformed p-value in two cell types, where the p-values are from differential expression or methylation analyses on each cell type.

Secondly, we want to emphasize that there are very strong correlations of cell type specific differential p-values in both DNA methylation and gene expression data, albeit the correlation is even stronger in the methylation data. In Figure 1, panel a shows the differential methylation p-values from male vs. female comparison, while panel b shows differential expression p-values before and after IFN-treatment. Clearly the sex comparison (panel a) has stronger signal, as expected. However, the differential expression results also show strong correlation among cell types. For example, the differential gene expression state between CD4 and CD8 have the highest correlation (Pearson correlation coefficient: 0.82). Other pairs of cell types also have Pearson correlation coefficients range from 0.33 to 0.75, which are very significant. If one considers the DE states only and tabulate the gene states in a 2x2 table, the odds ratios from the table are all very significant. For the gene expression data (panel b), one should pay attention to the points in the top right corners in each scatter plot, which represent genes that are differentially expressed in two cell types. It’s clear that there’s a high proportion of overlaps in those DE genes among any two cell types. Moreover, we have added a few extra examples in Additional file 1 Section S5 and Figure S1. Figure S1 a and c are from gene expression data, which show strong correlation in differential expression among cell types.

In addition, we want to clarify that we don’t model the DNA methylation as binary variable. Even though the methylation state in each single cell is binary, the methylation levels from bulk sample is an average of many (millions) cells, which is continuous but bounded between 0 and 1. For example, the DNA methylation array data measure the methylation level by beta or M value (we used beta value in real data analysis), both are continuous variable. The data modeling of DNA methylation beta values is not very different from modeling gene expression values from microarray. The binary random variable in CeDAR is used to model the DE/DM status, which has nothing to with observed data.

Another point we want to make is that cell type specific patterns in DNA methylation are not necessarily more distinct than gene expression. There are plenty cell type specific genes, as shown in many bulk and single cell gene expression analyses, thus building cell type hierarchy is also a common practice from either purified bulk or single cell gene expression^11, 12^. From this perspective, we believe it is reasonable to build cell type trees from expression data.

Overall based on our real data observations and the above reasons, we believe the proposed model is applicable to expression data.

*(Comment 5) In Figure 3, CeDAR-M and CeDAR showed high FDR compared to the other tools, which implies that other tools perform better in controlling type 1 error. Authors should clarify the evaluation metric and clarify the expression "However, compared to TOAST and TCA, the statistical inference of CeDAR-M and CeDAR-S are slightly more liberal in cell types with smaller proportions. (line: 198)"*

Thank you for this helpful suggestion. We acknowledge that TCA and TOAST perform better in controlling type 1 error than CeDAR methods in Figure 3, especially in cell types with small proportions and when sample size is small. “Liberal” is a statistical jargon which means that a statistical test tends to produce more false positives. To provide a clearer statement, we modified the result description in line 203 as following:

*“In cell types with smaller proportion, TOAST, TCA, csSAM and CellDMC have slightly better performance in controlling type I error than CeDAR. This indicates that the information borrowing across rare cell types tend to mildly inflate the false positives. But overall, all methods do not work well for cell types with small proportions, and the only solution for that is to increase the sample size.”*

*(Comment 6) Analysis on real dataset 3 (Figure 7.) ceDAR reports much more csDMCs compared to TOAST and TCA and a large proportion of csDMCs are shared TOAST and TCA. KEGG pathway enrichment showed that these csDMCs are related to the pathways relevant to the phenotype of interest. However, since ceDAR reports too many positive csDMCs it may contain false positive csDMCs so the authors should validate the csDMCs.*

Thank you for this comment. It is true that among the uniquely reported csDMCs by CeDAR methods, there could exist false positives. Since this dataset has no gold standard, we have to use csDMCs uniquely reported by CeDAR-M to check whether they are biologically meaningful. This is a common practice for majority of the method papers: one has to resort to things like pathway analysis to validate the results, even though they are not perfect. Our results show the top six enriched KEGG pathways have all been reported to be associated with RA disease and all have adjusted p-value smaller than 0.2, which implies that the CeDAR results are indeed biologically plausible.

To further strengthen the results, we identified three additional real datasets and performed the following analyses:

-	Detecting Down syndrome (DS) associated cell type specific differential methylation (csDM) sites from frontal cortex grey matter samples in DNA methylation data (GSE74886^1^).

-	Detecting Systemic lupus erythematosus (SLE) associated csDM sites from whole blood samples in DNA methylation data (GSE118114^2^).

-	Detecting smoking associated csDM sites from whole blood samples in two DNA methylation datasets (GSE42861^3^ and GSE40279^4^) separately.

For the former two real datasets (GSE74486 and GSE118144), the data include both bulk and pure cell type sequencing samples. The pure cell type profiles can be used to provide gold standard for validating the identified csDM sites from the bulk data. Accuracy was evaluated by true discovery rate (TDR) among top ranked sites. The latter two datasets (GSE42861 and GSE40279) do not have purified cellular profiles. Instead, we checked whether they could identify seven cell type specific smoking associated probes that were reported previously by Su et al.^5^. The new results are described in the main text Section Real data analysis – Other real data results and Additional file 1 Section S8. These results (Additional file 1 Figure S10 – S12) indicate that CeDAR method has better performance than other methods (TOAST, TCA, csSAM and CellDMC).

In practice, to avoid unexpected high false positive rate and find meaning DMCs/DEGs, users can use a more stringent threshold (e.g., higher posterior probability to be DE and larger effect size). In addition, in many high-throughput data analyses, the ranks of the features (genes or CpG sites) are often more important than the significance level itself. This is because when the scientists perform downstream experiments, they often pick a number of top ranked features that fit their budget. Our real data analyses show that that CeDAR can provide more accurate result (better ranks) than other methods for top ranked sites.

To summarize, we believe that even CeDAR could produce a little more false positives sometimes, its improved accuracy makes it a more desirable tool in many cases.

*(Comment 7) In Figure 1, the authors compared differential methylation pattern of female vs male and compared differential expression pattern of sclerosis patient before vs after IFN-g. It seems fairer to compare the methylation and expression pattern in same condition.*

Thank you for the suggestion. Comparing the cell type-specific DE/DM results for patients from similar conditions or diseases is not the focus of this work. We compare methylation or expression between conditions, and to show that the between-condition differences are correlated among cell types. The accuracy comparing the estimated DE/DM sites with the true sites from pure tissue profile or experimental results is our main approach for performance evaluation. We are not trying to compare different cell types in the same condition, or to compare methylation and expression in the same cell type under the same condition.

*(Comment 8) In Figure 2, if the figure aims to show how marginal probability is computed based on hierarchical cell type tree, the feature for the right most black circle in cell type 1,2,3 should not be marked as 1 since the marginal probability of the internal nodes is defined as a proportion of union of DEs across child cell types.*

Thank you for this comment. We first want to clarify that the following statement is entirely accurate: “*marginal probability of the internal nodes is defined as a proportion of union of DEs across child cell types*”. We define the DE status in all internal and leaf nodes in a probabilistic model. Thus, even if a parent node has non-zero probability to be “black”, it is still possible that all its children nodes are “non-black”.

To be specific, Figure 2 illustrates the “data generation” process of observed DE/DM state in cell types. From our hierarchical model, the DE state is determined “from top to bottom”: parent nodes determine their children nodes. So given an internal node being 1, there still exists a probability that cell type 1, cell type 2, and cell type 3 all have DE state 0.

**Response to Reviewer 3’s comments:**

*The authors present CeDAR, a method for cell-type specific differential methylation/expression analysis in the context of bulk data. CeDAR differs from other methods in that it explicitly takes a cell(type) lineage tree into account and can share information across tree nodes. The authors apply CeDAR to simulated and real data and find improved performance compared to current approaches.*

*GENERAL COMMENTS*

*The manuscript is generally clear and well written, and leveraging cell(type) lineage tree information to improve differential methylation and expression analyses is compelling in general. The authors motivate their method well and perform a detailed simulation study. Further on, noticeably more precise cell-type specific inferences from bulk data would have strong potential for impact; however, there are several issues with the current manuscript.*

We appreciate the positive comment.

*(Comment 1) No accessible software tool. The authors present a method and the major result reported is improved performance compared with current approaches. However, the manuscript does not mention a software tool, nor is one provided. If a readily usable tool is not provided, it is unclear what the community's interest in the manuscript should be.*

Thank you for this comment. Our method has been implemented in R/Bioconductor package “TOAST” (<https://www.bioconductor.org/packages/devel/bioc/html/TOAST.html>) with function *cedar()*, and a link to get access to it is provided in original submitted manuscript line 70 and line 615.

*(Comment 2) Reproducibility. While the authors describe their cedar method well, details about the real data analysis are missing, making reproduction of reported results difficult at best.*

Thank you for this helpful suggestion. We have modified the manuscript to provide more details about real data analyses. We have listed the version of all tools in the following table (also available in Additional file 1 Table S13). The parameters used in these functions are stated in the description of simulation and real data analysis in Methods section. Besides, we also provide all R scripts used to generate simulation and real data analysis results in our GitHub repository (<https://github.com/luxiao10/CeDAR_reproduction>).

Summary of tools used for performance evaluation in simulated and real data analysis

*(Comment 3) Simulation study. The approach for simulating data seems to recapitulate CeDAR's assumptions. This would be good for a proof of concept but bias the results in CeDAR's favor. Reporting CeDAR's performance in simulations matched to the other methods would be fair.*

Thank you for this comment. Our simulation process is almost identical to the simulation settings in TOAST and CellDMC, except adding correlation of DE states between cell types (all other methods assume independent DE state among cell types). Our Figure 4a shows the results when the DE states are independent among cell types. In that case, the simulations are exactly the same as in TOAST, and the results (Figure 4a) show that CeDAR methods have slightly better performances than other methods. To summarize, we have simulation settings identical to TOAST and CellDMC, and CeDAR works well in those settings.

*(Comment 4) Real data study. While the TDR curves are compelling, it is unclear what is learnt from the additionally recovered sites. Performance of CeDAR and TOAST on validated csDMCs seems very similar (Figure 7); form the pathway analysis using uniquely CeDAR csDMCs it is not clear if csDMCs of say TOAST would not highlight similar pathways, just via different sites. Showing a more direct impact on biological interpretation when using CeDAR vs other methods would be helpful.*

Thank you for this comment. As suggested, we used uniquely identified csDMC from TOAST and CellDMC (not reported by csSAM, TCA, CeDAR-S and CeDAR-M) in CD4 to do the KEGG pathway analysis. We found that there is no significantly enriched pathway from these csDMCs. This suggests that the csDMCs uniquely detected by CeDAR are more biologically meaningful. The following is the top five reported pathways from TOAST uniquely identified csDMCs, where all adjusted-pvalues are very large:

In order to provide more solid validation results from real data, we identified three additional real datasets and performed the following analyses:

-	Detecting Down syndrome (DS) associated cell type specific differential methylation (csDM) sites from frontal cortex grey matter samples in DNA methylation data (GSE74886^1^)

-	Detecting Systemic lupus erythematosus (SLE) associated cs-DM sites from whole blood samples in DNA methylation data (GSE118114^2^)

-	Detecting smoking status associated cs-DM sites from whole blood samples in two DNA methylation data (GSE42861^3^ and GSE40279^4^) separately

For the former two real datasets (GSE74486 and GSE118144), the data include both bulk and pure cell type samples, which can be used to provide gold standard for validating the identified csDM sites. Accuracy was evaluated by true discovery rate (TDR) among top ranked sites. The latter two datasets (GSE42861 and GSE40279) do not have purified cellular profiles. Instead, we checked whether they could identify seven cell type specific smoking associated probes that are reported by Su et al.^5^. The results are provided in the Additional file 1: Figure S10 for the DS data, Figure S11 for the SLE data, and Figure S12 for the smoking analysis.

In the analysis of detecting DS associated cs-DM sites (GSE74886), CeDAR-S achieves highest accuracy in detecting both glia-specific DM sites and neuron-specific DM sites. From Figure S10 in Additional file 1, we can observe that the difference of TDR between CeDAR-S and other methods (TOAST, TCA, csSAM, and CellDMC) among top ranked 5,000 sites is more than 20% in either glia or neuron cell type.

Same conclusion can be derived from analysis of detecting SLE associated csDM sites (GSE118144) that CeDAR-S and CeDAR-M have higher accuracy than all other methods (TOAST, TCA, csSAM, and CellDMC). From Figure S11 in Additional file 1, we can see that in large proportion cell type neutrophils (Neutro), CeDAR-S and CeDAR-M have higher TDR curve than other methods; in small proportion cell types, like CD4T, CD8T and B cells, the performances of all methods are not good, but only CeDAR-S and CeDAR-M can identify true DM sites among top ranked 5,000 sites.

In the analysis of detecting smoking associated cs-DM sites (GSE42861 and GSE40279), CeDAR-S can identify more smoking associated csDM sites than TOAST, TCA, csSAM and CellDMC. From Figure S12 in Additional file 1, we can observe that while TOAST, TCA, csSAM and CellDMC can identify four myeloid-specific sites but no lymphoid-specific sites, CeDAR-S can identify all myeloid-specific sites and one more lymphoid-specific site in Liu’s data (GSE42861). In Hannum’s data (GSE40279), CeDAR-S can identify four out of five myeloid-specific sites and one lymphoid-specific site, while the other four methods can at most identify only one myeloid-specific site.

Collectively, these additional analyses demonstrate that our proposed method can achieve more accurate and interpretable results than other existing methods.

The details of the analysis process and results are included in the Additional file 1 Section S8. We also provide some discussions on these results in the main text Section Real data analysis – Other real data results.

*SPECIFIC COMMENTS*

*(****Comment 5****) The authors use two real datasets to demonstrate strong correlations of differential methylation/expression between cell types, which, in principle, is not a lot of support for assuming this holds true in general. Broader support and/or a short discussion why that makes sense in the context differentiation and development would be helpful.*

Thank you for the comment. To provide more evidence and stronger support, we investigated three more datasets with gold standard to explore whether the correlation of DE/DM status among cell type exists. We generated figures similar to figure 1 and provided them in the Additional file 1 as Figure S1. Overall, these results strengthen our observation that the DE/DM status are indeed correlated among cell types, in particular similar cell types such as CD4 and CD8.

Furthermore, we want to clarify that CeDAR is designed for any study that needs to perform cell type specific differential analysis on bulk data. It is expected that CeDAR can improve power when there are correlation of DE/DM states among cell types. In practice, we believe such correlation is not limited to differentiation or development. It is determined by the cell types’ characteristics during certain biological process. For example, Mathys et.al reported that in late stage of Alzheimer’s disease (AD), genes upregulated were common across cell types and primarily involved in global stress response^13^. Tserel et al. reported that age-related methylation changes (measured by fold change) in CD4+ T cell and CD8+ T cell was in strong correlation and all top sites with the highest methylation differences between younger and older individuals shared by CD4+ and CD8+ T cells^14^. In a Graves’ disease (GD) study, Limbach et al. also reported that majority of top changed CpG sites associated with GD had differential methylation in both CD4+ and CD8+ T cells^15^.

We have added additional discussion in the main manuscript at the last paragraph of the Background section to discuss this point.

*(****Comment 6****) Figure 1 is convincing for the differential methylation part, but much less so for differential expression. This should be reflected in the corresponding discussion in the main text. On P 5 L 96 the authors write that results for expression are similar to those for methylation data, which does not reflect the figure 1.*

Thank you for this suggestion. In the manuscript, by “similar” we meant that the trends of Pearson’s correlation coefficients (PCC) and odds ratios (OR) derived from gene expression and methylation data are similar, that is, the PCCs are positive and the ORs are greater than 1. In the next sentence, we did mention that “the correlation strength appears to be weaker” in gene expression data. To avoid potential confusion to readers, we modified the statement as following:

*“These results indicate very strong correlation among cell types in their methylation difference between male and female. In gene expression data (Figure 1b), all PCCs are also significantly positive and all ORs are significantly greater than 1. The correlation strength appears to be weaker in the gene expression example than in the methylation data since the molecular differences between sexes (as considered in the methylation data) are likely to be much stronger than the treatment effects (as considered in the gene expression data).”*

*(****Comment 7****) The manuscript is a bit confusing when talking about DE/DM state on P 6 L 125/126, and P 17 L 434 (i.e., that a celltype can only be DE/DM if its parent is), in combination with the statement that "interaction terms are only included for celltypes deemed DE/DM". This implies that no DE/DM can emerge during differentiation, which does not make sense biologically. Rather, what seems to be the case is that the DE/DM state encodes the potential for DE/DM, where parent celltypes must have low effect size to enable their children's noticeable DE/DM. In any case, it would be good to make this point more clearly.*

Thank you for the comment. We agree that during differentiation, a gene may not show DE/DM in stem cell stage but appear in terminal cell types. However, in CeDAR, the concept of “parent node” of child nodes is not the same as “stem cell” for mature cells in developmental biology. In other words, even though there is a tree structure for cell types in CeDAR, the tree does not necessarily represent cell type lineage during differential or development. We introduce the tree merely as a statistical mean to capture the correlations of DE/DM states among cell types so that cell types close to each other on the tree would have more similar DE/DM states. Then by using conditional probabilities, we can introduce correlation among cell types sharing the same parent node, which would be helpful to increase power and accuracy for detecting csDE/csDM for low abundance cell types.

To avoid confusion, we have added the following sentences in the Section Methods – Prior probabilities for the DE/DM states in the main text:

“*Importantly, such cell type hierarchy is used merely as a statistical way to capture DE/DM state correlations among cell types. It does not necessarily* *represent the cell type lineage tree during differentiation or development*.”

*(****Comment 8****) In the simulation setup, it would be good to explore how the method performs when the tree is mis-specified (or mis-inferred). That is data is simulated with one tree and inferred with a different one.*

Thank you for this helpful suggestion. We have designed additional simulation studies to evaluate the impact of cell type tree misspecification. In the simulation, we applied either “correct” or “mis-specified” tree structure as input for CeDAR-M. “Correct” tree structure means applying the tree generating DE state in simulation data as input, while “mis-specified” tree means applying tree structures with cell types arbitrarily switched under nodes. Note that we did not significantly change the tree structure since we believe that even though there could be biases in tree estimation, the major structure should not change dramatically.

We performed the simulation study with six cell types (mean proportion of cell type 1 – 6: 0.63, 0.10, 0.11, 0.06, and 0.05) under different sample sizes per group (50, 100, 200). The evaluation process is similar as process described in Additional file 1 Section S1, except that all the tree structures are pre-specified without estimation. We specify the model with tree 1 as the true structure, while tree 2-5 are mis-specified trees as following.

For major cell type (cell type 1), mis-specified tree structure has some impact but overall, the difference is very small. For relatively smaller cell types, such as cell type 2, being mis-clustered with other minor cell types (cell type 4, 5, 6) will result in a decrease in accuracy. For example, in scenario with sample size 100 per group, the AUC-ROC of correct tree – “tree 1” is 0.919, while it is 0.871 for “tree 2”, in which cell type 2 is clustered with cell type 4 – 6. Similar trend can be observed for metric AUC-PR (“tree 1”: 0.630 vs. “tree 2”: 0.532), but the difference is small for metric MCC (“tree 1”: 0.413 vs. “tree 2”: 0.412). We can also observe increase of FDR (“tree 1”: 0.070 vs. “tree 2”: 0.102). Such change is because cell types with relatively small proportions (cell type 4, 5, 6) cannot provide accurate information as major cell type (cell type 1). In addition, for cell type 2, “tree 3” and “tree 4” have similar performance as “tree 1”, which indicate that when cell type 2 is correctly clustered with major cell type 1, the mis-specified tree structure in other sibling nodes have little impact on it.

Collectively, the major influence of “mis-specified” tree structure appears for minor cell types that are mis-clustered with other minor cell types. The reason might be the tree information plays a more important role in the csDE/DM detection for minor cell types compared with major cell types. We have added additional discussion in main text Section Simulation results – Evaluating the robustness of CeDAR – Robustness to cell type hierarchy estimation and added a new section to discuss the simulation in Additional file 1 Section S7. Related results are in Additional file 1 Figure S6, S7, Table S7.

*(****Comment 9****) Since the simulation setup appears very well matched to the CeDAR's assumptions, results are likely biased in CeDARs favor. It would be fair to simulate data according to TOAST and TCA and show results as well, at least for a subset of analyses.*

Thank you for this comment. We have addressed this concern in our response to your question 3. To summarize, we did have a simulation setting identical to those in TOAST and CellDMC, which is also very similar to the simulation setting of TCA. Under that scenario, we showed CeDAR still performs well.

*(****Comment 10****) For performance assessment, in addition to ROC curves a performance measure more geared towards imbalanced data (like average precision (i.e., area under the precision recall curve)) or mcc should be reported.*

Thank you for suggestion. We now have provided area under the precision recall curve and MCC in simulations in Additional file 1 Table S1 – S3, S8. The result supports the better performance of CeDAR.

*(****Comment 11****) It is interesting that the FDR is inflated for CeDAR with small sample size in the simulation study (P8 L 200), but that this is alleviated by using estimated trees and priors. This seems counter-intuitive, it would be good to comment.*

Thank you for this comment. In original manuscript P8 line 200, the reported cell type 6 FDR (0.23) is generated by using estimated tree and estimated prior for simulated data with sample size 50 per group. In Additional file 1 Figure S5, all results are derived from simulation data with sample size 100 per group (FDR of cell type 6 is 0.109). So, the alleviation of FDR value is due to increased sample size, this trend could be seen in Table S1. In order to avoid confusion of the results, we provided more detailed simulation setting information in legends of figures and tables.

A potential counter-intuitive observation in Additional file 1 Figure S5 is that using estimated prior probability has smaller observed FDR than using true prior probability. This is not always the case, and it is associated with the signal to noise ratio in the data. When noise level is relatively small, CeDAR with true prior probability has smaller FDR than CeDAR with estimated prior probability. However, when data noise is large, less DE genes/DM sites show true differential signal and more non-DE genes/non-DM sites show false differential signal in low abundant cell types. So, given the same cutoff, less DE/DM will be called in these low abundant cell types. This leads to an underestimation of the prior probability from the data, which leads to smaller FDR compared to for CeDAR with true prior probability.

To illustrate the effect of data noise on observed FDR from CeDAR, we performed additional simulation with different data noise levels (extremely low: 0.01, low: 0.1, normal: 1, high: 2). In the settings, normal level (noise level 1) is the setting we used in our reported simulations. We modify the noise level by multiplying 0.01, 0.1 or 2 to the standard deviation of both cell type specific gene expression and bulk expression.

We first performed the simulation on two cell types with proportion ratio 9:1. As can be seen from following table, we can see that when noise level is low (0.01, 0.1), the FDR of cell type 2 with true prior is still smaller with estimated prior (0.024 vs. 0.039, 0.025 vs. 0.037). But when noise level is larger (1, 2), we can observe larger FDR in cell type 2 with true prior (0.083 vs. 0.047, 0.225 vs. 0.126).

Same trend could be observed for scenario with six cell type. From following table, we can see that when data noise is small (noise level 0.01, 0.1), CeDAR with true prior probability has lower FDR than CeDAR with estimated prior probability (e.g., in cell type 2, 0.066 vs. 0.089, 0.069 vs. 0.080). When data noise is larger (noise level 1, 2), CeDAR with true prior probability has higher FDR than CeDAR with estimated prior probability (e.g., in cell type 2, 0.165 vs. 0.073, 0.345 vs. 0.206).

Overall, FDR inflation of CeDAR is alleviated with increasing sample size. The FDR difference between CeDAR with true prior probability and estimated probability is related with data noise. When data noise is large, CeDAR with estimated prior prob has smaller FDR and when data noise is small, CeDAR with true prior prob has smaller FDR. We have added a discussion in main text Section Simulation results – Evaluating the robustness of CeDAR – Robustness to cell type hierarchy estimation and added a section S6 in Additional file 1. The two tables are also in Additional file 1 as Table S5 and S6.

*(****Comment 12****) In the section about computation performance, it would be good to be explicit on how CeDAR scales with the number of samples (linear?) and with the number of celltypes (exponential?).*

Thank you for this question. CeDAR’s computational time increases quadratically with sample size and exponentially with cell type number, i.e., the computation time is.

We systematically benchmarked the computation time from different methods under a simulation scenario with 12,402 genes, but varying cell type numbers (4,6, and 8) and sample sizes (50, 100, and 200). The results shown in the table below is the average run time (in seconds) of five simulations. All simulations are run on a PC with Linux system with 2.80 GHz CPU and 8G RAM. Even though CeDAR’s computation is exponential to the number of cell types, since the number of cell types is usually not very large, CeDAR achieves good computational performances and is much faster than some competing methods such as csSAM and TCA in most cases.

*(Comment 13) P 25 L 593 (and others). Language like "minfi was used to pre-process raw data and call gold standard csDMC" is not sufficient. Either each step that is executed has to be described in detail and parameters need to be provided, or the authors can make their code available.*

Thank you for this suggestion. We have provided the details of our implementation in our manuscript and deposited our code in our Github site. Please see the reply to your question 2.

References:

1.	Mendioroz, M. et al. Trans effects of chromosome aneuploidies on DNA methylation patterns in human Down syndrome and mouse models. *Genome biology* 16, 1-26 (2015).

2.	Yeung, K.S. et al. Cell lineage-specific genome-wide DNA methylation analysis of patients with paediatric-onset systemic lupus erythematosus. *Epigenetics* 14, 341-351 (2019).

3.	Liu, Y. et al. Epigenome-wide association data implicate DNA methylation as an intermediary of genetic risk in rheumatoid arthritis. *Nat. Biotechnol.* 31, 142-147 (2013).

4.	Hannum, G. et al. Genome-wide methylation profiles reveal quantitative views of human aging rates. *Mol. Cell* 49, 359-367 (2013).

5.	Su, D. et al. Distinct epigenetic effects of tobacco smoking in whole blood and among leukocyte subtypes. *PloS one* 11, e0166486 (2016).

6.	Julià, A. et al. Epigenome-wide association study of rheumatoid arthritis identifies differentially methylated loci in B cells. *Hum. Mol. Genet.* 26, 2803-2811 (2017).

7.	Shen-Orr, S.S. et al. Cell type–specific gene expression differences in complex tissues. *Nat. Methods* 7, 287-289 (2010).

8.	Zheng, S.C., Breeze, C.E., Beck, S. & Teschendorff, A.E. Identification of differentially methylated cell types in epigenome-wide association studies. *Nat. Methods* 15, 1059-1066 (2018).

9.	Li, Z., Wu, Z., Jin, P. & Wu, H. Dissecting differential signals in high-throughput data from complex tissues. *Bioinformatics* 35, 3898-3905 (2019).

10.	Wu, H. & Ji, H. JAMIE: joint analysis of multiple ChIP-chip experiments. *Bioinformatics* 26, 1864-1870 (2010).

11.	Wu, Z. & Wu, H. Accounting for cell type hierarchy in evaluating single cell RNA-seq clustering. *Genome biology* 21, 1-14 (2020).

12.	Zheng, S.C. et al. A novel cell-type deconvolution algorithm reveals substantial contamination by immune cells in saliva, buccal and cervix. *Epigenomics* 10, 925-940 (2018).

13.	Mathys, H. et al. Single-cell transcriptomic analysis of Alzheimer’s disease. *Nature* 570, 332-337 (2019).

14.	Tserel, L. et al. Age-related profiling of DNA methylation in CD8+ T cells reveals changes in immune response and transcriptional regulator genes. *Scientific reports* 5, 1-11 (2015).

15.	Limbach, M. et al. Epigenetic profiling in CD4+ and CD8+ T cells from Graves' disease patients reveals changes in genes associated with T cell receptor signaling. *J. Autoimmun.* 67, 46-56 (2016).

**Second round of review**

**Reviewer 2**

Authors clearly addressed the issues from the previous manuscripts.

Here are a minor comment on the revised manuscript.

1. The novelty of ceDAR compared to the other studies is high performance in predicting csDE/csDM in cell types with small proportion. The title is a bit general to emphasize the novelty of this paper.

**Reviewer 3**

The authors have revised their manuscript and addressed most of my concerns. However, the comment that the authors show pathways uniquely highlighted by their cedar method, while missed by others, was not directly addressed. While the authors performed enrichment analyses on csDMCs that are uniquely found by cedar, this addresses a different question, namely if there are pathways enriched for uniquely cedar-found csDMCs. However, any such pathway could as well be also recovered by other methods (using different csDMCs). It would be valuable if the authors could clearly communicate if/which pathways are only recovered by cedar.

**Authors Response**

We thank the editor and reviewers for another round of thorough review to our manuscript “CeDAR: incorporating cell type hierarchy improves cell type specific differential analyses in bulk omics data” (GBIO-D-22-00185.R2).

Based on the editor and reviewers’ comments, we have carefully revised the manuscript. In this revision, we made following modifications:

1. The source code is available from Bioconductor package TOAST (<https://www.bioconductor.org/packages/release/bioc/html/TOAST.html>) with open-source license GPL-2.0. We have provided this information in the Availability of Data and Materials section of the manuscript.

2. We deposited the source codes used in the manuscript in a DOI-assigning repository on Zenodo with the DOI <https://doi.org/10.5281/zenodo.7272410>. We have provided this information in the Availability of Data and Materials section of the manuscript.

3. We provided formal references for datasets obtained from GEO and listed them in the reference list as with other references. We cited these newly created references in parallel to the respective papers in the Availability of Data and Materials section.

4. We provided a reference for source code in Zenodo and Bioconductor repository and a reference for the data analysis codes in Github repository, and included them in the reference list, as with other references.

5. We investigated the question about pathway analysis and presented additional details in the manuscript.

6. We used modified abstract for new manuscript.

In addition, we have made the following modifications on the formatting concerns:

1. We removed attached cover letter and response document.

2. Removed the line numbers.

3. Made figures with red/green color-blind friendly.

We addressed the individual points raised by the editor and reviewers, as detailed below. The editor/reviewers’ comments are in *italic blue*, and our replies are in regular font.

**Point-by-point responses to the reviewers’ comments:**

**Response to Reviewer 2’s comments:**

*(****Comment 1****) The novelty of CeDAR compared to the other studies is high performance in predicting csDE/csDM in cell types with small proportion. The title is a bit general to emphasize the novelty of this paper.*

Thank you for the suggestion. The main novelty of our work is to incorporate the cell type hierarchy in the cell type specific analysis in bulk omics data. Even though the performance improvement is the most dramatic in small proportion cell, we showed that CeDAR can improve the performances in cell types with all levels of abundance, including cell types with large proportions. Although we could use a title like “CeDAR: incorporating cell type hierarchy improves cell type specific differential analysis with small proportions”, it is overly specific and does not tell the whole story. Due to these reasons, we would like to keep the original title.

**Response to Reviewer 3’s comments:**

*(****Comment 1****) The authors have revised their manuscript and addressed most of my concerns. However, the comment that the authors show pathways uniquely highlighted by their CeDAR method, while missed by others, was not directly addressed. While the authors performed enrichment analyses on csDMCs that are uniquely found by CeDAR, this addresses a different question, namely if there are pathways enriched for uniquely CeDAR-found csDMCs. However, any such pathway could as well be also recovered by other methods (using different csDMCs). It would be valuable if the authors could clearly communicate if/which pathways are only recovered by CeDAR.*

Thank you for clarifying the concern about whether pathways enriched by CeDAR uniquely reported csDMCs are also recovered by other tools like TOAST.

We performed the enrichment analysis for csDMCs in CD4T called by different methods separately and checked whether the reported pathways are also called by other methods (TOAST, TCA, CellDMC, csSAM) with threshold FDR . The result is as following table:

As can be seen from above table, Sphingolipid signaling pathway is the only pathway uniquely called by CeDAR. TOAST reports five of the six pathways, while other three methods report less pathways. This result indicates that CeDAR can find unique csDMCs, leading to pathways and biological interpretations related to target phenotype that other methods cannot provide.

The table and corresponding explanation have been added in manuscript at the end of the section cell-type-specific differential methylation in rheumatoid arthritis study (Page 14-15, highlighted in red).
